# Supplementary material for: RNAthor – fast, accurate normalization, visualization and statistical analysis of RNA probing data resolved by capillary electrophoresis
Source: PLoS One. 2020 Oct 1;15(10):e0239287. doi: 10.1371/journal.pone.0239287 (PMC7529196; doi:10.1371/journal.pone.0239287)
Supplement: S1 File — (PDF) [file pone.0239287.s001.pdf]

RNAthor – fast, accurate normalization, visualization and statistical analysis of RNA probing data resolved by capillary electrophoresis

Julita Gumna[1], Tomasz Zok[2], Kacper Figurski[2], Katarzyna Pachulska-Wieczorek[1*], Marta Szachniuk[1,2*]

[1] Institute of Bioorganic Chemistry, Polish Academy of Sciences, Poznan, Poland

[2] Institute of Computing Science, Poznan University of Technology, Poznan, Poland

* Corresponding authors

Email: kasiapw@ibch.poznan.pl, mszachniuk@cs.put.poznan.pl

SHAPE_example_1_peaks.txt

seqnum	seq	RX.pos	RX.sigma	RX.area	RX.rms	BG.pos	BG.sigma	BG.area	BG.rms	(RX.area-BG.area)
6	G	9261	3.91	2569.8	96.8	9256	4.25	1054.1	19.9	1515.7
7	A	9246	4.02	7754.4	50.7	9242	4.12	2215.8	24.2	5538.7
8	A	9230	3.05	891.4	68.0	9225	5.19	20.4	42.7	871.0
9	C	9213	4.73	10583.9	145.9	9208	4.65	3532.0	53.9	7051.9
10	U	9198	4.87	20872.3	142.5	9194	5.69	7087.7	109.1	13784.6
11	U	9181	5.06	6704.5	108.3	9172	6.50	3647.2	130.7	3057.3
12	C	9165	4.92	14290.6	168.4	9161	4.65	4248.1	77.6	10042.4
13	U	9149	4.67	24957.5	201.7	9145	4.95	6180.2	67.6	18777.3
14	A	9133	3.93	9551.8	90.8	9129	3.95	1690.0	38.2	7861.9
15	G	9117	3.05	1243.3	77.9	9113	2.66	314.6	28.8	928.8
16	U	9102	3.75	5774.3	75.5	9098	4.30	1689.5	25.8	4084.9
17	A	9086	3.83	6657.7	73.6	9082	4.08	1986.1	28.0	4671.6
18	U	9068	3.12	775.8	102.8	9066	2.66	291.5	29.9	484.3
19	A	9054	4.40	7719.2	69.2	9051	5.45	2538.8	52.2	5180.4
20	U	9039	4.53	7468.8	154.2	9033	4.24	2157.1	39.6	5311.7
21	U	9023	5.20	17359.1	136.7	9018	6.26	4499.9	106.3	12859.2
22	C	9005	5.15	21565.6	197.9	9000	5.65	7083.6	169.2	14481.9
23	U	8990	4.91	25162.0	251.8	8987	5.23	8135.0	70.6	17027.0
24	G	8977	5.14	17892.4	227.9	8973	5.81	4633.3	57.4	13259.2
25	U	8961	5.30	14497.2	123.3	8958	5.56	4076.0	58.5	10421.1
26	A	8946	6.30	1522.2	93.8	8943	2.66	0.0	52.1	1522.2
27	U	8931	3.80	6411.6	138.0	8928	4.21	2103.7	30.9	4307.9
28	A	8914	3.05	3905.1	63.3	8912	2.66	494.9	34.8	3410.2
29	C	8897	3.20	2843.0	138.0	8894	3.04	375.7	37.7	2467.3
30	C	8880	4.57	16452.2	93.9	8876	5.00	4125.6	46.3	12326.6
31	U	8865	4.95	16488.1	179.1	8861	5.16	3983.0	51.4	12505.1
32	A	8849	4.90	25221.3	189.2	8846	4.93	4286.3	55.2	20935.0
33	A	8833	4.80	15751.4	138.5	8830	4.27	1525.0	39.6	14226.4
34	U	8817	4.43	17732.6	119.7	8814	4.01	3546.7	40.8	14185.9
35	A	8801	4.48	29249.7	199.9	8798	3.89	4071.5	43.7	25178.2
36	U	8785	4.60	16081.0	190.7	8781	4.08	1064.1	41.8	15016.9
37	U	8770	4.60	32748.9	189.2	8766	3.85	3303.7	48.7	29445.2
38	A	8754	4.65	17214.2	180.0	8751	3.76	3101.4	39.3	14112.9
39	U	8739	4.45	7094.5	62.1	8736	2.66	0.0	36.3	7094.5
40	A	8723	4.20	6384.9	42.7	8720	2.66	878.2	18.4	5506.6
41	G	8709	3.54	2902.8	47.6	8706	2.66	278.6	15.9	2624.2
42	C	8692	3.05	1125.9	31.4	8689	2.66	0.4	12.5	1125.5
43	C	8676	3.54	3635.5	55.9	8672	3.29	1022.4	18.0	2613.1
44	U	8660	4.60	3559.5	78.2	8656	3.11	928.8	23.7	2630.8
45	U	8644	4.60	6810.3	147.6	8641	4.71	912.8	45.2	5897.5
46	U	8630	4.52	23840.4	153.9	8627	4.50	3518.9	37.7	20321.5
47	A	8614	4.36	21748.5	95.7	8611	4.22	3203.8	35.7	18544.7
48	U	8598	4.67	8856.9	185.8	8594	4.13	1944.8	74.4	6912.1

49	C	8581	4.83	22151.5	154.0	8578	5.00	8634.9	87.1	13516.6
50	A	8566	4.82	22239.6	155.3	8563	5.35	5029.4	63.6	17210.2
51	A	8550	4.12	17923.9	247.0	8547	3.64	4353.7	85.9	13570.2
52	C	8533	3.04	7096.2	168.1	8531	2.21	738.4	30.2	6357.8
53	A	8516	3.44	10615.0	80.9	8514	1.78	5.3	7.6	10609.7
54	A	8500	3.71	7791.7	126.7	8496	1.78	107.7	13.5	7684.0
55	U	8485	4.22	13937.1	87.7	8482	3.79	2996.4	23.5	10940.7
56	G	8473	4.29	6819.9	82.8	8471	4.15	2160.3	34.5	4659.5
57	G	8458	4.32	1201.6	72.4	8456	4.64	63.3	18.5	1138.2
58	A	8443	2.82	1682.0	23.0	8442	2.05	197.4	12.3	1484.6
59	A	8428	2.82	817.2	18.6	8425	1.78	85.6	11.7	731.6
60	U	8413	2.90	1184.6	30.6	8411	1.99	40.4	9.3	1144.2
61	C	8396	3.42	4540.5	55.0	8394	2.89	1183.0	20.7	3357.5
62	C	8380	2.82	1760.1	72.4	8376	2.80	714.0	35.9	1046.1
63	C	8363	4.02	12960.5	98.9	8361	4.56	5195.9	59.3	7764.7
64	A	8347	3.82	17363.6	197.7	8345	4.75	4004.0	48.9	13359.6
65	A	8332	3.15	9307.7	139.8	8328	4.59	2635.3	46.0	6672.4
66	C	8315	3.49	13647.1	201.6	8312	4.20	5761.4	71.4	7885.7
67	A	8299	4.02	18327.0	83.8	8297	3.60	2697.7	41.6	15629.3
68	A	8283	4.02	23418.6	82.9	8281	2.97	3093.0	44.4	20325.6
69	U	8268	4.05	9086.8	125.3	8267	1.78	0.0	30.7	9086.8
70	U	8254	4.17	24737.7	115.8	8252	2.84	2106.4	36.0	22631.3
71	A	8239	4.10	14648.1	88.8	8236	1.78	467.2	34.6	14180.9
72	U	8223	3.50	5562.8	148.2	8220	1.78	52.1	18.7	5510.8
73	C	8207	3.10	4353.7	50.0	8204	2.80	1011.5	15.5	3342.2
74	U	8191	2.82	1166.7	48.4	8189	1.78	129.9	17.4	1036.8
75	C	8176	3.91	6826.3	79.3	8174	3.80	1879.6	34.5	4946.7
76	A	8161	4.25	17195.3	45.8	8159	4.20	3022.6	27.6	14172.7
77	A	8146	4.20	11439.8	51.7	8143	3.75	1575.3	30.2	9864.5
78	C	8129	3.68	4658.3	82.3	8127	3.97	1739.3	27.8	2919.0
79	A	8114	3.85	7637.7	74.3	8111	4.20	2794.8	17.1	4842.9
80	U	8099	4.00	2543.4	53.1	8097	3.98	953.1	17.9	1590.4
81	U	8084	3.05	422.7	34.4	8081	2.34	211.1	19.1	211.5
82	C	8068	3.59	3385.4	37.1	8066	3.10	874.9	17.0	2510.5
83	A	8053	3.67	7317.3	66.1	8050	2.93	1013.9	20.9	6303.4
84	C	8036	3.80	7133.1	100.9	8033	3.80	1704.0	48.4	5429.1
85	C	8019	4.25	14172.4	70.8	8016	4.40	3734.1	37.8	10438.3
86	C	8003	4.25	21301.3	67.1	8001	4.35	6293.0	38.6	15008.3
87	A	7988	4.25	18111.9	60.5	7986	3.89	2114.7	52.7	15997.3
88	A	7972	3.87	9545.0	135.9	7970	2.51	1271.2	30.8	8273.9
89	U	7958	3.56	6609.9	51.2	7956	1.55	117.3	10.4	6492.6
90	U	7942	3.48	6477.5	89.9	7939	1.55	254.4	9.9	6223.1
91	C	7926	3.05	4293.0	55.5	7924	1.95	658.9	14.2	3634.1
92	U	7910	3.05	3413.5	51.4	7907	1.79	417.3	11.9	2996.2
93	C	7894	3.58	6577.4	121.9	7891	2.21	851.1	35.1	5726.3
94	A	7877	4.00	14819.1	70.3	7874	3.51	2500.4	38.8	12318.7
95	U	7862	4.05	11733.1	110.9	7859	3.75	2731.4	27.5	9001.7
96	G	7848	4.15	3075.6	83.5	7844	2.64	429.1	20.3	2646.5

97	G	7832	3.05	1579.0	43.6	7828	1.55	170.6	19.2	1408.4
98	U	7816	3.36	5970.7	88.7	7814	3.44	2045.7	23.9	3924.9
99	A	7799	3.56	5188.6	158.7	7795	3.49	1740.0	22.1	3448.6
100	G	7785	3.36	0.0	129.0	7782	2.49	0.0	18.5	0.0
101	C	7772	3.91	4375.4	33.1	7769	3.30	953.2	22.7	3422.3
102	G	7759	3.46	3789.8	57.1	7757	2.49	186.2	18.6	3603.7
103	C	7742	3.36	4427.3	56.9	7740	2.97	1104.9	30.8	3322.4
104	C	7727	3.60	9600.2	65.1	7724	3.35	2415.0	36.9	7185.2
105	U	7711	3.36	3156.7	76.2	7708	2.49	526.3	21.8	2630.4
106	G	7697	3.36	1766.1	97.7	7693	4.39	34.3	30.9	1731.7
107	U	7682	3.98	14625.7	49.5	7679	4.06	3912.1	37.7	10713.6
108	G	7668	3.96	7173.1	40.3	7665	3.76	2087.4	29.8	5085.8
109	C	7653	3.66	7375.4	64.4	7651	3.92	2661.8	36.5	4713.6
110	U	7638	3.83	9887.9	71.1	7635	4.06	3380.0	29.8	6507.9
111	U	7623	3.91	13706.7	71.9	7620	4.15	3374.1	32.4	10332.6
112	C	7606	3.98	16937.0	117.1	7603	4.11	5473.8	30.7	11463.2
113	G	7592	4.11	6415.3	65.9	7589	3.90	1788.2	19.5	4627.1
114	G	7576	4.21	3609.3	83.6	7573	4.20	1329.8	21.8	2279.5
115	U	7561	4.11	17259.9	116.6	7559	4.39	4983.0	32.5	12277.0
116	U	7547	4.07	21696.1	89.5	7545	4.34	6474.4	43.7	15221.7
117	A	7533	4.21	734.9	66.4	7531	4.39	29.6	37.6	705.3
118	C	7518	3.39	2666.7	40.6	7516	4.06	1148.7	18.0	1518.0
119	U	7505	3.76	6554.5	45.5	7502	3.92	2362.3	26.9	4192.2
120	U	7490	3.50	2391.8	51.1	7487	2.49	480.1	15.8	1911.7
121	C	7474	3.02	1180.0	52.1	7470	2.43	55.7	15.7	1124.4
122	U	7458	3.75	8869.4	44.9	7456	4.38	2281.0	34.6	6588.4
123	A	7443	4.26	20598.4	149.0	7440	4.28	4450.3	39.7	16148.0
124	A	7427	4.14	30218.3	144.8	7425	4.00	3453.2	30.3	26765.1
125	G	7414	3.98	9654.6	75.8	7411	4.23	715.3	33.3	8939.3
126	G	7401	4.09	11188.4	91.5	7398	3.81	3266.9	45.7	7921.4
127	A	7386	3.83	4649.4	105.4	7384	2.81	888.7	22.9	3760.8
128	A	7372	3.04	1880.7	98.2	7369	4.58	226.3	35.3	1654.4
129	G	7358	4.14	11697.9	82.4	7355	4.00	3695.8	36.2	8002.1
130	U	7343	3.52	2794.7	83.9	7341	2.43	279.8	15.3	2514.9
131	C	7328	2.86	1510.8	41.5	7324	2.43	55.2	11.3	1455.6
132	C	7313	3.26	5115.4	50.6	7310	2.83	1320.1	17.5	3795.3
133	A	7297	2.86	1947.1	62.8	7296	2.43	26.3	17.7	1920.7
134	C	7281	3.16	6090.6	86.9	7279	2.58	1444.9	17.0	4645.7
135	A	7266	2.86	2596.1	55.0	7263	2.43	123.2	16.6	2472.9
136	C	7251	3.22	6191.6	102.2	7248	2.43	1090.3	15.5	5101.3
137	A	7236	3.50	8588.7	63.5	7233	2.59	757.9	36.0	7830.8
138	A	7221	4.01	18145.1	154.9	7218	3.66	3506.3	25.0	14638.7
139	A	7205	4.01	24641.0	160.3	7203	3.66	4121.4	31.7	20519.6
140	U	7190	3.48	8794.2	84.7	7187	2.43	1062.4	37.2	7731.8
141	C	7175	4.01	17326.7	109.7	7173	4.33	4880.6	54.1	12446.1
142	A	7160	4.01	33115.5	77.3	7158	4.19	7754.4	39.2	25361.1
143	A	7145	3.86	18936.0	97.3	7143	3.09	2213.6	43.5	16722.4
144	G	7131	3.18	4930.5	90.4	7128	1.95	34.0	11.3	4896.6

145	A	7115	3.27	4466.3	89.7	7111	1.95	41.3	15.4	4425.0
146	U	7100	3.45	8194.7	89.8	7098	2.95	1692.1	21.3	6502.5
147	C	7086	3.06	4719.8	50.2	7083	2.62	929.6	25.8	3790.3
148	C	7071	3.30	6736.7	34.1	7069	2.70	1500.6	28.4	5236.1
149	G	7057	3.66	4680.1	80.7	7054	3.95	592.4	46.2	4087.7
150	U	7043	3.75	18619.8	58.6	7040	3.75	3847.1	63.7	14772.6
151	U	7029	3.70	14737.4	93.6	7026	2.57	1054.5	36.1	13682.9
152	A	7014	3.42	10391.1	104.0	7012	3.56	26.9	37.8	10364.1
153	G	7001	3.75	23092.2	188.5	6998	3.30	3347.8	43.2	19744.5
154	A	6986	3.66	14053.4	223.0	6984	1.95	317.7	25.5	13735.8
155	C	6970	3.18	9833.9	61.3	6967	2.57	1816.2	32.4	8017.6
156	G	6957	2.90	3478.0	84.7	6954	1.95	213.2	17.6	3264.9
157	U	6943	2.90	4136.0	99.1	6940	1.95	0.0	13.6	4136.0
158	U	6929	3.25	8258.9	71.1	6926	2.81	1684.6	24.6	6574.3
159	U	6915	2.90	962.0	137.0	6911	4.15	166.9	51.0	795.2
160	C	6899	3.71	17279.0	115.5	6897	3.90	4947.6	42.3	12331.5
161	A	6885	3.57	12446.6	80.0	6883	3.39	2598.5	25.0	9848.1
162	G	6871	2.90	710.5	40.4	6868	1.95	22.0	14.3	688.5
163	C	6857	2.90	1271.4	26.6	6858	1.95	27.1	5.5	1244.3
164	U	6844	3.26	2590.9	35.3	6842	1.95	293.2	8.8	2297.7
165	U	6830	3.25	3065.5	36.2	6828	1.95	70.9	9.7	2994.6
166	C	6814	3.16	4028.0	49.9	6811	1.95	648.4	27.7	3379.6
167	C	6799	3.40	7238.2	36.4	6797	3.05	2069.3	31.6	5169.0
168	A	6785	3.36	6841.7	37.6	6782	2.65	1331.8	43.5	5510.0
169	A	6771	3.55	7448.2	83.3	6769	2.65	1391.8	37.7	6056.4
170	A	6756	3.75	14285.7	40.1	6753	2.92	1285.3	24.5	13000.5
171	A	6741	3.73	20219.2	47.5	6738	3.44	2356.4	33.1	17862.8
172	C	6725	3.62	14092.3	47.8	6722	3.48	3586.8	36.0	10505.5
173	A	6710	3.68	11103.5	37.5	6707	3.68	2081.6	35.0	9021.9
174	G	6697	3.73	13009.3	40.0	6694	3.88	3442.1	33.2	9567.2
175	A	6682	3.71	14431.7	74.2	6679	3.68	3140.3	48.8	11291.4
176	A	6667	3.67	15640.1	144.5	6665	3.22	2294.1	31.8	13346.0
177	G	6654	2.50	1212.2	91.9	6651	2.65	273.8	22.9	938.5
178	A	6640	2.95	6303.5	92.2	6637	3.13	2028.3	29.4	4275.2
179	A	6625	2.84	4040.5	78.6	6622	2.74	1354.2	28.9	2686.3
180	U	6610	3.08	4364.2	46.6	6607	3.13	1677.6	23.4	2686.6
181	G	6598	2.50	342.2	59.7	6593	3.91	111.5	37.7	230.7
182	U	6583	3.65	9143.7	130.7	6580	3.72	3997.7	34.6	5146.1
183	G	6570	3.71	7654.1	98.6	6568	3.68	2840.0	30.3	4814.2
184	A	6555	3.17	3704.4	46.2	6552	3.34	1448.1	33.7	2256.3
185	G	6542	3.57	8782.2	57.5	6540	3.72	3186.4	45.2	5595.8
186	A	6527	3.68	7532.7	57.2	6524	3.67	1581.0	46.2	5951.7
187	A	6512	3.57	10206.1	78.7	6510	3.44	2297.5	28.9	7908.6
188	G	6500	3.53	4621.8	49.8	6498	3.77	1894.6	29.1	2727.2
189	G	6488	2.50	1504.5	37.7	6485	2.99	543.4	26.2	961.0
190	C	6473	5.53	583.4	204.7	6471	2.99	152.3	16.4	431.1
191	U	6456	5.53	4025.7	348.2	6458	3.68	2228.8	14.3	1796.9
192	U	6445	1.98	11.9	32.8	6443	2.99	29.2	11.0	-17.3

193	C	6435	3.88	1996.8	94.1	6430	3.88	965.1	24.8	1031.7
194	C	6418	3.59	13841.6	83.1	6416	3.84	5687.6	30.4	8154.0
195	A	6403	3.55	9773.3	60.4	6401	3.93	3402.9	39.8	6370.4
196	C	6388	3.60	9291.9	62.6	6385	3.80	2983.6	58.3	6308.3
197	U	6374	3.55	10615.4	61.5	6372	3.56	3395.5	30.2	7219.9
198	A	6360	3.34	4933.0	54.5	6357	3.38	925.5	33.5	4007.5
199	A	6345	3.42	10769.5	60.2	6343	3.80	4151.8	44.9	6617.7
200	G	6333	3.55	6230.2	48.5	6330	3.80	3091.7	38.1	3138.5
201	G	6321	3.60	10036.8	44.0	6319	3.61	4692.4	34.6	5344.4
202	C	6307	1.98	23.6	41.9	6305	3.99	17.9	42.2	5.7
203	U	6294	3.60	13674.2	50.9	6291	3.80	4955.9	62.8	8718.3
204	A	6281	3.55	10440.5	51.2	6279	2.99	1315.1	42.2	9125.4
205	A	6267	3.37	13001.0	59.5	6265	2.99	1656.2	32.7	11344.8
206	C	6252	3.16	7619.7	142.9	6250	2.99	1645.3	30.8	5974.4
207	U	6239	1.98	1120.7	91.1	6236	2.99	373.5	36.5	747.2
208	C	6225	2.99	8898.5	76.5	6223	3.54	3926.7	36.2	4971.9
209	U	6212	2.81	3531.0	84.8	6209	3.61	1186.5	27.2	2344.5
210	C	6197	3.00	9560.1	129.5	6195	3.69	3316.0	21.7	6244.1
211	A	6184	2.95	9029.9	119.9	6181	3.19	2356.5	45.2	6673.5
212	A	6169	2.76	7686.0	171.1	6167	3.23	1856.0	39.4	5830.0
213	C	6155	2.80	7998.3	190.4	6152	3.29	2874.9	32.0	5123.4
214	A	6141	3.35	11551.2	77.4	6138	3.29	1953.4	32.8	9597.8
215	G	6129	3.27	17799.9	134.0	6127	3.23	3327.0	35.5	14472.9
216	A	6116	2.80	6588.6	115.4	6112	2.07	0.1	34.5	6588.5
217	C	6101	3.32	13030.0	198.4	6098	3.56	3731.7	35.0	9298.3
218	A	6087	3.50	30729.4	214.3	6085	3.47	6874.7	50.0	23854.7
219	A	6073	2.80	6484.2	120.9	6070	3.69	140.8	82.4	6343.4
220	C	6059	3.27	21334.1	169.1	6056	3.69	7514.9	79.7	13819.2
221	A	6046	3.41	23078.0	247.7	6043	3.22	4277.5	51.7	18800.6
222	A	6032	3.06	4189.5	209.9	6030	2.07	0.0	34.1	4189.5
223	C	6018	3.09	12902.3	107.5	6016	2.87	3706.4	41.8	9195.8
224	A	6004	3.09	12090.5	94.1	6001	2.07	1273.0	45.9	10817.4
225	C	5988	2.80	4604.6	157.1	5985	2.07	340.9	53.7	4263.8
226	C	5973	3.55	18525.5	138.9	5971	3.45	5791.0	69.5	12734.5
227	U	5961	3.74	20466.1	167.6	5958	3.38	4523.1	75.3	15943.1
228	G	5950	3.77	3371.2	247.5	5947	2.07	0.0	33.5	3371.2
229	C	5938	3.32	9792.3	118.4	5935	2.07	908.2	30.7	8884.1
230	U	5925	3.41	24317.2	165.8	5923	2.50	2697.9	42.7	21619.3
231	U	5913	3.29	13509.1	120.5	5910	2.07	459.5	42.8	13049.6
232	C	5898	3.09	12417.0	173.6	5896	2.07	1646.0	33.3	10771.0
233	A	5884	3.03	12868.4	183.0	5881	2.07	1018.5	34.2	11849.9
234	U	5870	3.09	12847.0	156.3	5867	2.07	740.7	38.3	12106.3
235	C	5855	2.91	11236.7	125.8	5852	2.38	2488.3	37.7	8748.4
236	A	5839	3.06	10827.0	128.5	5837	2.64	1789.7	49.9	9037.2
237	G	5826	3.09	11884.2	137.8	5824	2.88	3261.0	45.1	8623.3
238	C	5814	2.82	5927.7	112.6	5812	2.53	1756.4	34.8	4171.3
239	U	5802	2.66	1663.7	92.3	5798	2.02	1.3	16.1	1662.4
240	G	5792	2.49	2039.7	72.7	5790	2.02	303.7	14.7	1736.0

241	U	5780	2.62	4503.8	97.9	5777	2.02	793.5	17.4	3710.3
242	U	5766	2.62	4575.5	100.9	5763	2.09	465.1	29.2	4110.4
243	C	5752	2.93	10030.8	146.5	5749	2.91	2046.0	44.0	7984.8
244	C	5737	3.20	15039.2	155.8	5734	3.77	4895.6	52.1	10143.6
245	A	5724	3.39	24172.5	266.5	5720	3.57	6545.2	97.7	17627.3
246	G	5713	3.58	22463.9	170.7	5710	3.26	7086.2	57.9	15377.7
247	A	5700	3.48	7816.5	145.4	5698	3.42	2058.5	30.1	5758.0
248	G	5690	3.20	9022.0	93.0	5687	3.40	1641.5	20.4	7380.5
249	A	5678	3.29	16400.5	90.0	5675	3.04	2608.9	47.7	13791.6
250	A	5665	2.51	2240.4	78.1	5662	2.02	50.5	12.5	2189.9
251	C	5650	2.76	4526.3	69.8	5648	2.24	961.3	26.6	3565.0
252	C	5636	2.80	4146.4	76.2	5633	3.14	1829.3	33.2	2317.0
253	C	5622	3.18	8395.7	92.0	5619	3.37	3976.2	32.3	4419.5
254	C	5608	3.15	6371.2	85.3	5605	3.42	2135.2	25.4	4236.0
255	C	5594	3.05	9981.6	71.1	5591	3.22	4295.6	41.1	5686.0
256	A	5580	3.00	7034.1	86.8	5578	2.92	2541.6	31.8	4492.6
257	U	5568	2.49	1627.4	78.2	5565	2.08	441.7	34.2	1185.7
258	C	5554	2.88	7118.6	72.0	5552	2.82	2646.1	35.3	4472.5
259	A	5541	2.48	1510.8	75.0	5539	2.43	158.9	37.6	1351.9
260	U	5530	3.38	8687.2	100.8	5527	3.23	3002.9	31.2	5684.3
261	G	5518	3.48	2519.6	86.4	5517	2.43	605.0	24.7	1914.6
262	C	5504	3.23	9259.1	129.0	5502	3.37	3129.2	31.1	6130.0
263	C	5492	3.18	13496.4	94.5	5489	3.16	3405.9	45.1	10090.5
264	U	5480	2.48	699.6	63.1	5476	2.43	78.9	27.5	620.7
265	C	5466	3.18	7291.2	78.5	5464	3.12	2858.6	26.8	4432.7
266	U	5454	2.55	2804.8	73.0	5452	2.43	702.5	32.1	2102.4
267	C	5440	3.05	4558.9	99.9	5438	3.02	1730.1	45.9	2828.8
268	C	5426	3.18	14244.9	96.6	5424	3.37	5586.9	45.8	8657.9
269	U	5414	3.11	4399.8	58.3	5411	3.42	1715.3	30.7	2684.5
270	C	5401	3.34	6452.9	42.6	5398	3.37	2295.7	38.7	4157.2
271	A	5388	3.29	16870.9	80.3	5386	3.28	3399.5	41.3	13471.3
272	A	5375	3.28	20515.7	92.3	5372	2.80	2192.4	52.6	18323.4
273	C	5361	2.62	3805.9	70.8	5358	2.43	681.5	36.5	3124.3
274	C	5347	3.34	6601.1	99.0	5344	3.15	2822.5	43.6	3778.6
275	U	5335	3.23	16179.5	176.3	5333	3.24	6565.7	44.4	9613.8
276	G	5324	2.51	3442.8	93.1	5322	3.07	1312.3	40.5	2130.5
277	C	5311	2.48	3313.6	41.2	5308	2.43	1123.5	25.9	2190.1
278	U	5298	2.48	1749.3	89.1	5295	2.43	137.9	35.5	1611.4
279	U	5286	3.19	11096.3	96.6	5283	3.38	2930.2	30.3	8166.1
280	C	5272	3.29	14921.1	79.7	5269	3.24	5091.4	52.2	9829.6
281	A	5259	3.10	4679.9	65.9	5256	2.43	1270.0	36.0	3409.9
282	G	5249	3.16	3124.6	48.3	5246	2.62	706.1	24.2	2418.4
283	U	5238	3.03	7156.8	89.9	5236	2.73	2128.1	36.3	5028.7
284	A	5226	2.73	2471.9	50.2	5223	2.45	228.4	27.7	2243.6
285	C	5213	2.73	2457.5	112.9	5209	2.45	585.5	34.0	1871.9
286	C	5199	3.28	18395.7	167.8	5197	3.14	5803.5	43.7	12592.1
287	A	5187	2.73	3971.8	86.7	5185	2.45	617.5	37.8	3354.2
288	C	5173	2.73	1031.6	61.8	5170	2.45	259.2	23.7	772.4

289	C	5159	3.08	7531.6	57.5	5156	3.00	2329.7	33.2	5201.9
290	U	5147	2.92	3345.6	60.3	5144	2.45	766.6	24.6	2579.0
291	C	5135	3.28	7749.4	58.6	5132	3.48	2056.5	37.8	5692.8
292	C	5121	3.16	13219.0	89.4	5118	3.28	4757.9	62.0	8461.1
293	A	5108	3.25	8127.3	119.5	5105	3.20	2571.2	64.6	5556.1
294	C	5093	3.53	15458.9	44.2	5090	3.76	5312.7	37.8	10146.2
295	A	5080	3.48	34142.7	112.7	5077	3.62	8589.1	107.0	25553.6
296	G	5068	3.31	15120.2	165.6	5065	2.91	2309.3	52.0	12810.9
297	A	5054	3.35	23807.5	253.7	5051	3.53	3950.9	28.3	19856.6
298	A	5040	3.53	21500.9	296.0	5036	3.33	3701.5	51.6	17799.4
299	U	5028	3.68	14600.5	202.4	5025	2.67	1453.0	32.0	13147.5
300	G	5018	2.73	3733.8	106.9	5015	2.58	905.7	25.9	2828.1
301	G	5008	3.58	4600.3	153.3	5005	2.96	1605.7	77.3	2994.6
302	G	4997	3.35	17671.4	277.0	4995	3.33	6414.4	58.1	11257.0
303	C	4984	3.07	9171.8	142.3	4982	3.19	3173.3	47.7	5998.5
304	C	4972	3.09	3250.9	68.4	4969	2.45	827.1	31.3	2423.8
305	G	4962	2.88	1694.0	53.0	4959	2.03	194.2	16.9	1499.8
306	U	4950	2.68	2007.1	55.0	4947	2.03	312.7	20.1	1694.4
307	A	4936	2.68	4541.9	56.7	4933	2.03	1156.4	20.9	3385.5
308	C	4921	2.68	1707.9	53.2	4918	2.03	314.2	15.4	1393.6
309	C	4908	3.43	4440.6	95.0	4906	2.94	1629.7	64.4	2810.9
310	C	4897	3.16	12165.1	203.6	4895	3.22	5013.9	67.0	7151.2
311	A	4884	2.95	8557.0	137.0	4881	3.02	2831.2	45.9	5725.8
312	C	4871	3.30	15312.7	173.0	4868	3.22	5444.4	44.6	9868.3
313	A	4858	3.53	4591.6	77.3	4855	2.77	1182.6	39.3	3409.0
314	G	4847	2.68	963.7	95.8	4845	2.03	64.4	13.1	899.3
315	C	4836	2.68	4437.6	60.5	4833	2.47	1795.8	42.0	2641.8
316	A	4823	3.30	6291.7	144.1	4819	3.18	2499.1	47.5	3792.6
317	G	4812	3.16	17199.6	240.9	4809	3.22	5646.5	58.9	11553.1
318	U	4801	3.16	28293.6	258.3	4798	3.22	5803.7	59.1	22489.9
319	G	4789	3.30	2972.1	145.9	4786	2.13	904.6	43.1	2067.5
320	C	4776	2.68	6134.4	104.0	4773	2.78	2626.9	38.5	3507.5
321	A	4763	2.68	4864.1	74.9	4760	2.61	1787.6	26.1	3076.4
322	U	4752	3.03	5699.7	136.9	4749	3.14	2617.5	60.5	3082.2
323	G	4742	3.03	10809.2	117.8	4739	3.25	4579.8	46.7	6229.4
324	A	4731	2.68	2004.6	56.6	4727	2.15	718.1	36.2	1286.5
325	U	4720	2.98	286.3	42.1	4717	2.03	75.0	17.5	211.4
326	G	4710	2.77	3416.7	55.2	4707	3.30	1168.4	28.3	2248.3
327	A	4699	2.95	11367.0	138.4	4695	2.59	2306.3	50.0	9060.7
328	C	4685	3.01	9515.1	117.7	4682	3.14	3608.8	74.7	5906.3
329	C	4671	3.27	16663.0	168.9	4668	3.55	6786.9	45.3	9876.1
330	C	4658	3.20	20815.6	264.4	4655	3.28	7472.5	115.6	13343.2
331	A	4647	2.81	11764.2	186.7	4644	2.60	1826.5	51.2	9937.7
332	A	4636	3.05	16829.2	210.5	4632	3.00	2433.1	55.1	14396.1
333	A	4624	3.05	20657.6	188.5	4621	2.62	2379.0	38.0	18278.6
334	A	4612	2.74	9596.6	146.3	4608	2.05	747.7	35.3	8848.9
335	C	4598	2.65	4759.6	129.9	4594	2.60	1157.7	46.7	3602.0
336	C	4586	3.08	16237.2	176.2	4583	3.00	5852.5	47.5	10384.7

337	A	4574	3.05	18841.9	219.3	4571	2.77	3216.6	60.9	15625.2
338	A	4562	2.99	12332.3	159.6	4559	2.05	1264.6	38.2	11067.7
339	G	4551	2.89	6704.5	116.3	4547	2.05	0.2	38.6	6704.3
340	C	4539	2.65	7708.4	81.5	4535	2.53	2642.4	49.4	5066.0
341	C	4527	2.65	6254.9	83.8	4523	2.25	1748.1	38.5	4506.9
342	A	4515	2.65	5671.7	86.4	4512	2.05	1013.9	31.4	4657.9
343	A	4502	2.65	5952.9	102.5	4498	2.32	914.3	33.3	5038.6
344	U	4491	2.65	9506.9	113.4	4487	2.45	1474.0	33.2	8032.9
345	C	4480	2.65	2090.8	154.9	4475	3.15	687.6	56.2	1403.2
346	C	4466	2.95	16255.7	160.2	4463	2.99	4659.5	61.9	11596.3
347	A	4454	2.79	9316.9	118.6	4451	2.43	1576.8	40.2	7740.1
348	U	4442	2.65	6039.2	145.5	4438	2.05	553.2	46.0	5485.9
349	C	4428	3.08	11549.5	189.1	4424	3.04	3723.3	50.6	7826.2
350	U	4416	2.95	21152.9	222.8	4413	3.04	6201.0	48.2	14951.9
351	G	4406	2.55	1541.0	93.9	4402	2.24	397.1	27.2	1143.9
352	G	4396	2.55	883.5	63.6	4392	3.04	258.1	26.4	625.4
353	U	4385	2.77	6918.8	84.7	4381	2.67	2073.4	53.4	4845.4
354	U	4373	3.07	5777.4	86.3	4371	2.46	1622.8	94.2	4154.5
355	G	4363	3.74	8253.2	281.0	4361	3.32	6440.0	142.8	1813.3
356	G	4354	3.15	47385.6	495.9	4351	3.64	19071.1	131.1	28314.4
357	U	4344	2.76	15672.9	231.3	4339	3.39	5132.5	184.1	10540.3
358	C	4331	3.25	15944.5	251.1	4327	3.21	5533.2	131.8	10411.2
359	A	4319	3.17	27989.4	304.7	4315	3.24	6754.3	96.5	21235.0
360	U	4309	3.03	22598.2	231.5	4305	2.91	5784.0	62.6	16814.2
361	U	4298	3.15	3082.3	86.6	4294	2.24	596.7	47.0	2485.6
362	U	4286	2.95	7471.0	102.3	4283	2.24	673.3	25.8	6797.7
363	U	4275	2.67	4362.6	67.5	4271	2.24	662.6	16.8	3699.9
364	A	4263	2.57	5165.7	73.2	4259	2.29	1243.3	31.6	3922.4
365	C	4253	4.20	1073.6	238.3	4249	4.63	568.3	86.8	505.3
366	G	4243	3.93	6792.0	72.5	4239	4.04	3042.5	34.7	3749.4
367	G	4233	2.88	4037.5	172.8	4229	2.60	979.5	52.5	3058.1
368	A	4223	2.55	1548.1	87.6	4219	2.24	47.3	23.6	1500.8
369	C	4211	2.79	5381.3	75.4	4208	2.44	1733.9	19.0	3647.4
370	A	4199	2.55	6530.5	75.4	4196	2.24	147.8	22.7	6382.7
371	C	4185	2.55	6104.6	78.0	4181	2.24	1574.8	28.6	4529.9
372	C	4173	2.55	2304.7	91.0	4168	2.36	495.9	28.2	1808.8
373	C	4161	2.77	9716.8	187.0	4157	2.67	3645.0	48.8	6071.9
374	A	4150	3.07	16862.9	121.5	4147	2.85	3598.6	58.1	13264.4
375	U	4139	2.99	10769.8	147.9	4136	3.47	2645.4	50.4	8124.5
376	C	4126	3.01	13670.1	186.9	4122	3.23	6564.8	85.9	7105.3
377	U	4115	3.25	26897.8	134.1	4112	3.52	11310.9	144.9	15586.9
378	A	4103	3.12	23513.1	278.7	4099	3.28	8738.9	239.4	14774.1
379	U	4093	3.26	32309.4	426.0	4090	3.32	14192.1	220.2	18117.3
380	G	4083	3.37	14099.8	517.7	4080	3.91	3404.7	138.2	10695.1
381	A	4073	2.45	7033.4	158.3	4069	2.37	1754.9	57.4	5278.5
382	U	4061	2.69	6063.0	89.0	4058	2.37	615.5	26.0	5447.5
383	U	4051	2.45	3219.3	117.3	4047	3.21	1410.8	39.6	1808.4
384	C	4039	2.69	6867.7	248.0	4034	3.35	4885.1	33.7	1982.6

385	C	4029	4.50	436.2	270.5	4029	4.52	1088.2	158.5	-651.9
386	G	4020	4.25	17294.5	243.7	4018	4.37	9822.8	108.0	7471.7
387	U	4009	2.45	3234.7	141.1	4006	4.57	1155.1	90.5	2079.7
388	A	3998	3.15	9314.4	133.0	3994	3.05	3145.7	77.6	6168.7
389	U	3989	3.21	11624.4	138.3	3986	2.80	2434.5	33.4	9189.9
390	A	3978	2.45	2553.2	120.0	3975	2.37	0.0	29.5	2553.2
391	C	3966	2.93	5867.1	117.0	3963	2.91	3097.1	38.1	2770.0
392	A	3956	2.81	12424.8	181.5	3953	2.72	2484.4	46.2	9940.4
393	C	3943	2.45	2627.6	85.3	3939	2.37	1201.3	36.6	1426.2
394	C	3931	2.60	4934.9	152.0	3927	3.07	2267.6	39.5	2667.3
395	U	3921	3.25	6114.7	86.4	3917	2.73	1420.1	39.5	4694.5
396	U	3911	2.76	14033.7	226.1	3908	3.31	4323.3	64.1	9710.4
397	A	3900	3.07	16811.1	226.9	3896	3.25	4268.1	45.4	12543.0
398	U	3889	3.09	11313.6	150.5	3885	3.21	3088.6	38.5	8225.1
399	C	3877	3.00	10881.8	134.5	3873	3.21	3961.3	43.2	6920.5
400	A	3866	3.28	10403.0	132.1	3862	2.99	2368.9	54.2	8034.1
401	A	3854	3.09	18026.3	282.3	3851	3.31	6037.5	55.7	11988.9
402	A	3844	2.99	6526.4	240.5	3839	3.34	1480.4	28.2	5046.0
403	U	3832	3.47	7297.0	140.5	3828	3.29	2218.6	65.8	5078.4
404	G	3824	2.37	3252.8	107.8	3823	5.06	2156.1	44.9	1096.7
405	U	3817	2.59	2639.0	79.3	3812	2.94	1027.1	29.3	1611.9
406	C	3803	3.91	8366.0	133.8	3799	4.16	5121.3	60.1	3244.7
407	G	3792	3.46	1983.9	132.6	3788	3.22	1440.2	77.0	543.7
408	C	3781	1.67	328.2	19.2	3777	2.11	368.4	25.2	-40.3
409	C	3770	2.57	1009.0	61.8	3765	2.39	1079.0	37.4	-70.0
410	U	3759	3.22	6799.5	81.9	3754	4.13	4262.8	63.0	2536.7
411	A	3748	3.22	8273.1	86.6	3748	2.11	175.6	33.7	8097.5
412	U	3739	4.12	2010.6	101.8	3743	4.11	4347.9	43.1	-2337.3
413	G	3730	3.47	3427.7	92.6	3727	3.91	1778.8	46.5	1648.9
414	U	3722	2.94	4908.4	57.8	3717	3.11	2034.1	39.6	2874.3
415	A	3712	3.07	6335.8	78.8	3707	2.86	1581.5	27.8	4754.2
416	C	3702	3.47	2287.4	96.3	3702	4.85	423.9	47.1	1863.5
417	U	3691	3.43	5359.6	95.3	3690	5.06	997.2	74.6	4362.4
418	U	3681	3.43	10907.3	69.1	3676	4.03	3808.5	78.7	7098.7
419	U	3671	3.47	4930.4	58.9	3667	2.55	1562.3	42.6	3368.1
420	C	3660	3.37	1199.3	63.0	3657	3.21	40.4	33.9	1158.9
421	C	3650	3.40	4661.4	62.2	3645	2.83	3293.9	75.4	1367.5

SHAPE_example_2_peaks.txt

seqnum	seq	RX.pos	RX.sigma	RX.area	RX.rms	BG.pos	BG.sigma	BG.area	BG.rms	(RX.area-BG.area)
6	G	9290	4.54	3856.3	46.7	9285	5.33	1903.1	50.2	1953.2
7	A	9275	3.98	8520.5	65.0	9272	4.45	2603.8	30.5	5916.7
8	A	9261	3.91	1311.8	57.4	9256	4.10	115.6	34.0	1196.2
9	C	9243	4.41	8506.0	90.1	9239	5.19	2714.0	61.2	5792.0
10	U	9228	4.10	16193.6	111.4	9223	4.10	4288.0	77.8	11905.6
11	U	9210	3.05	677.0	118.7	9206	4.10	311.4	68.4	365.6
12	C	9194	4.96	16211.7	174.6	9189	5.05	4573.8	50.5	11637.9
13	U	9179	4.86	29677.4	228.0	9175	4.91	7655.7	59.5	22021.8
14	A	9163	4.91	11461.0	198.4	9159	4.95	2021.1	56.8	9439.9
15	G	9148	3.42	2660.4	93.0	9143	4.10	718.7	31.6	1941.7
16	U	9132	4.46	6787.3	72.8	9127	4.50	2119.0	31.9	4668.3
17	A	9116	4.22	7594.6	88.9	9113	4.35	2224.5	27.4	5370.1
18	U	9101	3.05	425.5	68.7	9095	4.10	184.1	25.2	241.3
19	A	9084	4.31	6322.5	73.2	9079	4.10	1387.7	27.5	4934.8
20	U	9068	4.63	6079.5	95.0	9063	4.42	1179.4	38.5	4900.1
21	U	9053	4.72	13538.2	128.0	9048	5.19	2492.1	44.9	11046.1
22	C	9036	5.06	18345.2	209.3	9032	5.12	4924.1	63.9	13421.1
23	U	9020	5.22	24162.1	224.0	9017	5.60	8255.3	105.2	15906.8
24	G	9006	5.12	17806.8	91.6	9001	5.09	5516.9	91.6	12289.9
25	U	8993	5.51	11167.2	138.7	8990	5.14	3659.2	82.4	7508.0
26	A	8977	5.25	5290.3	224.7	8974	6.55	1476.6	81.2	3813.7
27	U	8961	4.80	8825.4	72.4	8958	4.52	2459.0	26.5	6366.4
28	A	8945	3.99	6192.9	129.3	8942	4.10	1021.3	36.3	5171.6
29	C	8928	3.05	2024.6	96.8	8925	4.10	176.6	29.3	1847.9
30	C	8910	3.05	8050.5	88.7	8907	4.56	3326.5	26.2	4724.0
31	U	8895	3.77	8080.7	148.5	8891	4.81	2816.1	41.0	5264.6
32	A	8879	4.05	18482.7	74.7	8875	4.60	3521.1	44.8	14961.7
33	A	8863	3.65	11562.1	92.6	8859	4.60	2205.1	42.4	9357.0
34	U	8848	4.28	14791.8	184.8	8844	4.85	4501.7	46.3	10290.1
35	A	8831	4.60	27127.0	149.1	8828	4.78	5091.0	47.5	22036.1
36	U	8815	4.60	14956.7	160.1	8812	4.75	2385.6	40.2	12571.1
37	U	8800	4.48	30571.4	147.2	8797	4.64	4298.5	36.8	26272.9
38	A	8784	4.60	15265.7	124.4	8781	4.53	3838.3	36.3	11427.4
39	U	8769	4.16	5981.5	46.6	8764	2.35	285.2	22.3	5696.3
40	A	8753	4.05	5708.0	36.4	8750	3.92	1530.5	14.3	4177.5
41	G	8739	3.50	2662.4	45.7	8736	3.43	702.1	16.4	1960.3
42	C	8722	3.05	1248.8	37.9	8719	2.35	40.0	8.6	1208.8
43	C	8706	3.05	2811.7	37.8	8703	2.35	371.1	11.5	2440.6
44	U	8690	3.05	2440.0	71.5	8687	2.35	419.8	18.6	2020.2
45	U	8675	4.65	6541.5	120.6	8670	2.98	675.4	23.1	5866.1
46	U	8660	4.65	23857.6	131.3	8657	4.22	3237.9	34.1	20619.7
47	A	8644	4.70	22202.6	143.1	8640	4.40	2806.3	31.5	19396.3
48	U	8627	4.58	10252.7	151.7	8622	3.78	2244.6	61.6	8008.0

49	C	8611	4.95	26781.1	274.8	8607	5.80	7991.7	97.5	18789.4
50	A	8595	4.85	24293.1	357.8	8591	4.80	5115.5	116.3	19177.6
51	A	8580	4.70	20850.2	206.4	8577	4.19	4610.5	27.9	16239.8
52	C	8562	4.65	11063.5	225.3	8560	3.52	1579.6	48.0	9483.8
53	A	8546	4.33	14858.9	96.8	8544	2.76	1417.9	27.1	13441.1
54	A	8529	4.28	10847.5	98.2	8526	4.14	1412.1	37.4	9435.4
55	U	8515	4.24	15478.6	77.1	8512	4.15	4096.1	31.3	11382.5
56	G	8503	4.38	7947.4	80.8	8501	4.24	2795.9	41.9	5151.5
57	G	8488	4.60	1305.5	73.6	8486	4.74	61.1	16.5	1244.3
58	A	8473	3.00	1464.1	25.5	8471	1.64	120.9	3.5	1343.2
59	A	8457	3.00	1025.4	31.9	8456	1.64	88.1	3.7	937.4
60	U	8442	3.16	1403.7	37.9	8440	1.64	142.5	12.5	1261.2
61	C	8426	3.30	4449.1	44.3	8424	3.11	1429.4	17.7	3019.7
62	C	8409	3.10	2376.0	106.2	8405	3.97	706.3	34.3	1669.7
63	C	8393	4.20	14577.0	71.2	8391	4.19	4772.4	28.9	9804.6
64	A	8378	4.25	20411.9	91.0	8376	4.24	3601.1	26.3	16810.8
65	A	8362	4.28	15230.3	99.8	8359	4.29	2284.9	27.0	12945.4
66	C	8345	4.20	18186.1	104.9	8343	4.38	5735.5	32.2	12450.6
67	A	8330	4.25	21046.7	102.2	8327	4.29	3681.2	39.3	17365.5
68	A	8314	4.23	24715.5	72.5	8312	4.02	5125.8	35.2	19589.7
69	U	8299	4.15	8941.2	64.4	8296	4.14	762.0	41.9	8179.2
70	U	8285	4.00	23388.1	102.9	8282	3.68	3606.9	78.1	19781.2
71	A	8270	3.76	12591.8	169.8	8268	2.24	1105.9	31.7	11485.9
72	U	8254	3.00	3689.6	131.5	8251	1.64	53.3	16.3	3636.3
73	C	8238	3.00	4040.9	49.3	8236	2.79	1096.9	27.8	2944.1
74	U	8222	3.00	836.3	50.1	8220	1.92	319.0	19.9	517.4
75	C	8207	3.94	6604.1	85.2	8205	3.97	1704.3	30.0	4899.8
76	A	8192	4.23	17071.2	41.3	8190	4.06	2819.5	26.0	14251.7
77	A	8177	4.25	11480.1	46.0	8175	3.68	1479.6	24.2	10000.5
78	C	8160	3.89	4566.4	57.1	8158	4.21	1535.9	29.7	3030.5
79	A	8145	3.90	7102.6	46.2	8142	3.89	2611.5	31.0	4491.1
80	U	8130	3.63	2588.5	45.4	8128	3.63	1062.4	13.6	1526.0
81	U	8114	3.15	482.7	33.8	8111	2.58	97.7	16.3	385.1
82	C	8100	3.49	3711.8	55.1	8098	2.76	579.1	14.8	3132.7
83	A	8084	3.95	8051.8	55.1	8082	2.80	1028.3	19.0	7023.5
84	C	8067	3.87	7222.3	62.1	8065	3.41	1440.7	45.9	5781.6
85	C	8051	4.20	14710.4	74.0	8048	4.21	3399.2	35.9	11311.2
86	C	8035	4.29	21704.0	81.0	8033	4.21	5886.6	36.3	15817.4
87	A	8020	4.40	19018.3	93.1	8018	3.92	2023.3	43.3	16995.0
88	A	8004	4.35	10738.6	86.4	8002	2.67	1293.6	31.5	9445.0
89	U	7989	4.00	7982.0	83.7	7987	1.76	334.6	14.4	7647.4
90	U	7974	3.70	7347.0	60.9	7972	1.76	375.6	13.2	6971.3
91	C	7958	3.31	4869.8	49.1	7955	1.76	641.1	16.9	4228.7
92	U	7942	3.15	3165.7	49.3	7939	1.76	356.2	16.9	2809.5
93	C	7926	3.63	6362.9	129.3	7923	1.99	603.5	28.8	5759.5
94	A	7909	3.96	13900.1	94.9	7907	3.56	2204.5	33.2	11695.5
95	U	7894	3.96	11143.5	99.4	7891	3.80	2930.5	21.6	8213.0
96	G	7880	4.15	2907.5	83.8	7878	2.87	582.1	19.2	2325.4

97	G	7864	3.15	1443.0	43.7	7863	1.96	199.1	19.1	1243.9
98	U	7849	3.35	5639.2	86.1	7846	2.95	1767.0	26.9	3872.1
99	A	7831	3.70	5386.1	139.9	7828	2.76	1333.7	19.8	4052.5
100	G	7817	4.25	2.7	125.2	7815	1.76	0.0	17.2	2.7
101	C	7805	3.82	4481.8	47.6	7802	3.19	1112.8	16.8	3368.9
102	G	7792	3.61	4083.4	71.2	7789	2.62	397.2	17.7	3686.2
103	C	7775	3.61	4579.7	70.4	7772	3.30	1088.7	19.8	3491.0
104	C	7759	3.85	10681.5	42.2	7757	3.72	2671.6	21.4	8009.9
105	U	7743	3.61	4502.7	50.7	7741	3.17	1053.5	25.5	3449.1
106	G	7729	3.80	2763.2	91.7	7726	3.57	523.8	30.3	2239.4
107	U	7714	4.04	14174.4	71.3	7712	4.17	3860.0	18.8	10314.4
108	G	7700	3.68	7542.1	79.7	7698	4.02	2093.3	21.7	5448.8
109	C	7686	3.77	7114.9	72.4	7683	3.66	2284.9	29.2	4830.0
110	U	7670	3.96	10365.3	54.9	7668	3.79	3282.0	34.5	7083.4
111	U	7655	3.85	12793.0	64.4	7652	3.97	2682.6	46.9	10110.4
112	C	7638	3.85	15765.9	65.8	7635	3.74	4243.0	65.3	11522.9
113	G	7624	3.61	4821.8	99.0	7622	3.17	790.4	29.8	4031.4
114	G	7609	3.61	2416.9	124.3	7605	3.17	407.3	47.4	2009.6
115	U	7593	4.31	16638.5	142.8	7590	4.26	4195.0	47.4	12443.5
116	U	7578	4.03	21065.0	239.0	7576	4.02	5922.8	53.6	15142.2
117	A	7565	3.61	515.1	59.4	7562	3.17	0.0	13.4	515.1
118	C	7551	3.61	2782.2	49.2	7548	4.26	1125.1	23.3	1657.1
119	U	7537	3.76	5985.2	35.4	7534	4.02	2457.1	33.5	3528.1
120	U	7522	3.61	2358.3	43.1	7519	3.17	783.4	20.9	1574.9
121	C	7506	3.61	1204.3	67.5	7503	3.17	198.3	24.7	1006.0
122	U	7490	3.91	8284.2	64.5	7488	3.88	2301.1	20.8	5983.2
123	A	7475	4.23	19393.3	143.5	7473	4.27	4317.6	31.0	15075.7
124	A	7459	4.18	29999.1	191.7	7457	4.15	3461.0	43.5	26538.2
125	G	7446	3.75	9293.3	44.1	7444	3.17	881.2	35.7	8412.1
126	G	7433	4.12	10474.1	63.2	7431	4.10	3315.9	33.6	7158.2
127	A	7418	3.55	4338.7	93.7	7416	2.90	901.6	35.0	3437.1
128	A	7404	2.97	1626.3	104.2	7401	4.25	229.2	42.0	1397.1
129	G	7390	4.01	10968.1	66.9	7387	4.04	4166.9	47.9	6801.2
130	U	7375	3.37	2551.7	72.3	7373	2.49	476.8	17.6	2074.9
131	C	7360	2.97	1454.4	45.3	7357	2.49	140.3	10.9	1314.1
132	C	7345	3.17	5302.7	63.4	7343	2.84	1323.4	22.9	3979.3
133	A	7330	2.97	1711.1	64.6	7327	2.49	511.0	25.9	1200.2
134	C	7314	3.27	6083.9	62.7	7311	3.24	2343.1	33.1	3740.7
135	A	7298	2.99	3148.0	55.6	7296	3.01	916.2	31.1	2231.8
136	C	7283	3.53	6828.6	78.9	7280	3.29	1993.2	30.0	4835.5
137	A	7268	3.82	9550.7	42.9	7266	3.33	1408.6	40.7	8142.1
138	A	7253	3.82	17704.4	58.7	7251	3.89	3749.1	25.8	13955.3
139	A	7238	3.92	24612.0	59.9	7236	3.84	4533.3	25.3	20078.7
140	U	7223	3.92	9657.1	85.3	7220	3.99	1507.7	33.1	8149.4
141	C	7208	3.87	17712.3	102.5	7205	4.04	4642.5	48.7	13069.9
142	A	7193	3.97	33974.4	102.5	7191	3.99	7322.8	52.1	26651.6
143	A	7178	3.97	19664.4	108.0	7176	3.45	2349.0	46.3	17315.4
144	G	7164	3.25	5033.1	83.7	7160	2.49	24.6	12.9	5008.5

145	A	7147	3.01	4203.5	62.7	7144	2.49	120.2	14.3	4083.3
146	U	7133	3.27	7510.3	71.2	7131	3.34	2010.9	23.9	5499.4
147	C	7119	3.09	4354.8	49.6	7116	3.09	1419.7	26.0	2935.2
148	C	7104	3.35	6900.6	28.7	7102	3.49	2093.2	34.6	4807.5
149	G	7090	3.57	5084.0	61.9	7086	3.66	1464.0	27.2	3620.0
150	U	7076	3.82	18465.1	68.5	7074	4.24	4870.2	47.0	13595.0
151	U	7062	3.90	15937.6	111.7	7059	3.65	2325.8	50.2	13611.8
152	A	7046	3.95	12554.6	126.4	7045	4.11	1031.1	61.5	11523.5
153	G	7033	3.80	23671.8	160.7	7031	4.05	4763.2	45.0	18908.6
154	A	7019	3.59	15074.8	80.8	7016	3.25	1597.3	44.2	13477.5
155	C	7003	3.50	10651.1	52.5	7001	3.75	3384.6	53.6	7266.5
156	G	6990	3.40	4759.9	47.7	6987	4.05	1939.2	24.4	2820.7
157	U	6976	3.50	5175.3	45.7	6973	3.88	1826.6	17.0	3348.7
158	U	6962	3.40	8537.3	66.1	6960	3.77	2781.3	25.9	5756.0
159	U	6948	2.95	1004.1	99.5	6945	4.05	528.4	37.0	475.7
160	C	6932	3.70	16997.2	79.9	6930	3.89	4720.5	42.3	12276.7
161	A	6918	3.66	12600.6	49.2	6916	3.59	2541.3	22.6	10059.2
162	G	6903	2.95	608.7	39.5	6902	1.45	1.6	13.3	607.2
163	C	6890	2.95	1003.5	24.9	6889	1.45	14.5	5.0	989.0
164	U	6877	3.30	2700.5	39.1	6875	2.00	334.2	10.0	2366.3
165	U	6863	3.19	2903.7	50.4	6860	1.59	166.2	15.1	2737.6
166	C	6847	2.95	3653.8	57.9	6845	1.45	164.1	13.7	3489.7
167	C	6832	2.95	6391.2	61.5	6830	2.00	746.7	17.4	5644.5
168	A	6818	2.95	5545.8	60.7	6815	1.45	98.4	17.8	5447.5
169	A	6803	2.95	5730.6	99.3	6801	1.45	379.3	8.9	5351.2
170	A	6789	3.35	12488.1	136.8	6787	1.45	263.0	18.6	12225.1
171	A	6774	3.50	18406.2	115.7	6771	2.23	1601.6	43.5	16804.6
172	C	6758	3.40	12687.8	122.7	6755	2.75	2509.1	47.7	10178.7
173	A	6743	3.65	10734.8	127.4	6740	3.18	1616.0	55.8	9118.7
174	G	6729	3.52	11888.1	150.4	6727	3.60	3092.1	21.6	8796.0
175	A	6715	3.53	12925.9	141.3	6712	3.50	2783.1	30.2	10142.8
176	A	6700	3.65	15686.9	98.2	6698	3.15	2405.5	27.7	13281.4
177	G	6687	2.85	1154.3	66.7	6684	2.75	430.7	27.8	723.6
178	A	6672	2.85	5850.2	65.3	6670	3.12	1960.2	24.6	3890.0
179	A	6657	2.85	3972.0	67.0	6655	3.12	1486.9	26.0	2485.0
180	U	6642	2.85	4301.7	58.9	6640	3.67	2070.0	17.5	2231.7
181	G	6629	2.85	211.0	64.3	6626	4.19	634.3	32.0	-423.4
182	U	6615	3.57	8162.2	60.0	6612	4.07	4233.3	47.0	3928.9
183	G	6602	3.55	6649.0	69.1	6600	3.76	3206.2	18.7	3442.8
184	A	6587	3.28	3693.0	57.8	6585	3.77	1856.4	23.1	1836.6
185	G	6574	3.45	8596.0	57.1	6572	3.87	3654.2	22.9	4941.8
186	A	6560	3.41	7116.6	60.5	6557	3.71	1721.5	31.2	5395.1
187	A	6545	3.66	10814.7	55.7	6542	3.72	2630.8	30.5	8183.9
188	G	6532	3.80	4779.9	41.5	6530	3.72	2052.4	37.1	2727.5
189	G	6520	2.89	1492.0	45.4	6517	4.27	572.2	27.1	919.7
190	C	6506	2.85	464.7	37.5	6503	2.75	165.1	22.8	299.6
191	U	6493	3.47	4958.7	48.1	6490	3.87	2347.5	12.4	2611.2
192	U	6479	3.60	0.4	45.0	6476	2.75	103.7	8.5	-103.3

193	C	6465	3.03	2494.9	48.0	6462	3.96	1080.9	16.1	1414.0
194	C	6450	3.65	13816.4	41.7	6448	3.92	5927.6	26.2	7888.8
195	A	6435	3.60	10071.5	50.9	6433	3.87	3592.6	27.6	6478.9
196	C	6420	3.55	9611.9	76.8	6418	3.76	3220.7	24.1	6391.2
197	U	6406	3.48	11075.4	77.1	6404	3.87	3907.5	21.6	7167.9
198	A	6392	3.28	4893.0	44.6	6389	3.55	1212.2	28.8	3680.8
199	A	6377	3.40	9986.5	47.4	6375	3.86	4116.4	39.2	5870.1
200	G	6365	3.48	6231.8	52.9	6362	3.87	3386.5	42.6	2845.3
201	G	6353	3.54	9721.3	56.7	6351	3.76	4778.1	34.0	4943.2
202	C	6339	3.11	28.8	61.5	6337	4.11	139.1	42.0	-110.3
203	U	6326	3.58	13611.4	90.2	6323	3.89	5208.8	59.2	8402.6
204	A	6313	3.54	10915.8	108.8	6311	3.20	1374.8	33.1	9540.9
205	A	6299	3.51	13858.5	86.0	6296	2.90	1818.4	34.8	12040.0
206	C	6284	3.13	8215.8	130.1	6282	2.66	1585.7	23.1	6630.0
207	U	6271	3.11	1449.9	118.6	6267	3.46	411.2	38.8	1038.7
208	C	6256	3.11	8413.8	108.4	6254	3.36	4108.9	44.2	4304.9
209	U	6243	3.11	4338.7	99.3	6241	3.09	1114.7	41.6	3224.0
210	C	6229	3.35	10996.8	58.1	6227	3.28	3112.9	33.4	7883.9
211	A	6215	3.11	10731.0	101.6	6213	2.94	2145.9	23.7	8585.1
212	A	6201	3.11	10045.6	89.6	6198	2.90	1964.3	29.5	8081.3
213	C	6186	3.35	10696.1	99.9	6184	3.36	2914.3	40.5	7781.8
214	A	6172	3.58	13588.0	109.1	6169	3.51	2251.6	30.2	11336.4
215	G	6160	3.41	19112.4	189.5	6158	3.41	4136.8	26.4	14975.6
216	A	6147	3.11	8073.5	100.3	6145	2.66	759.1	20.4	7314.4
217	C	6132	3.56	13765.3	178.1	6130	3.90	4360.9	52.9	9404.4
218	A	6118	3.46	31212.5	246.0	6116	3.71	7117.9	67.1	24094.5
219	A	6104	2.79	7256.3	158.2	6101	3.70	781.8	70.3	6474.5
220	C	6090	3.46	21716.6	207.9	6087	3.70	7357.8	71.6	14358.8
221	A	6076	3.46	24172.1	184.4	6074	3.32	4551.1	36.3	19621.0
222	A	6063	2.53	3723.4	171.6	6061	1.59	7.8	42.2	3715.6
223	C	6049	3.10	12946.6	162.5	6047	2.81	3558.7	48.0	9387.9
224	A	6034	3.16	12686.2	149.1	6032	1.90	1407.8	45.5	11278.4
225	C	6019	2.58	5050.1	149.9	6016	1.59	244.6	48.1	4805.5
226	C	6004	3.72	19247.2	202.8	6001	3.39	5432.9	39.1	13814.3
227	U	5991	3.91	21146.7	104.3	5989	3.35	4961.6	35.1	16185.1
228	G	5980	2.69	3094.4	217.0	5978	2.63	0.0	53.4	3094.4
229	C	5968	3.63	10165.2	138.0	5966	2.09	1055.2	39.3	9110.0
230	U	5955	3.48	24610.1	231.1	5953	2.59	2578.4	35.8	22031.7
231	U	5943	3.33	14397.8	135.2	5940	1.59	368.4	33.7	14029.4
232	C	5928	3.23	13678.8	161.8	5926	1.59	863.0	11.5	12815.8
233	A	5914	3.13	14467.9	145.3	5911	1.59	24.7	6.0	14443.2
234	U	5900	3.23	14595.3	104.8	5897	1.59	0.0	9.9	14595.3
235	C	5885	3.08	12365.7	101.0	5882	1.71	1036.5	23.4	11329.2
236	A	5869	3.15	11967.3	119.6	5866	1.75	884.8	40.2	11082.5
237	G	5856	3.08	11544.8	129.7	5854	2.59	2303.8	53.3	9241.0
238	C	5844	2.75	7053.6	141.7	5842	2.45	1813.2	36.6	5240.5
239	U	5832	2.53	1810.1	119.5	5827	1.59	223.7	15.3	1586.4
240	G	5822	2.53	2018.2	91.0	5820	2.06	527.5	20.4	1490.7

241	U	5810	2.53	4401.4	71.4	5807	2.29	959.3	21.8	3442.1
242	U	5797	2.69	4807.7	101.2	5794	2.29	591.3	26.1	4216.4
243	C	5782	2.97	10578.1	125.0	5779	2.78	1961.7	46.6	8616.4
244	C	5767	3.19	16492.6	167.4	5764	3.67	4567.5	65.4	11925.1
245	A	5754	3.38	23707.9	260.2	5750	3.48	5788.0	95.1	17919.9
246	G	5743	3.53	20807.0	184.8	5740	3.29	6785.1	47.6	14021.9
247	A	5730	3.39	7867.6	171.0	5728	3.26	2402.2	41.1	5465.4
248	G	5720	3.16	9329.6	130.8	5718	3.49	1983.7	48.9	7345.9
249	A	5708	3.33	17483.1	130.6	5705	3.27	2787.9	54.5	14695.2
250	A	5694	2.53	2183.4	118.9	5691	2.29	164.4	20.8	2019.0
251	C	5680	2.72	4568.6	67.5	5678	2.33	881.5	19.3	3687.1
252	C	5666	2.98	5900.8	109.8	5664	2.98	1788.8	46.2	4112.0
253	C	5651	3.23	9514.1	98.5	5649	3.52	4020.7	25.1	5493.3
254	C	5638	3.15	7545.4	85.9	5635	3.46	2528.2	30.0	5017.2
255	C	5624	3.23	10939.1	74.8	5621	3.36	4157.1	37.6	6782.0
256	A	5610	3.16	8005.6	82.7	5607	3.17	2888.7	48.3	5116.9
257	U	5598	2.67	2175.0	68.6	5595	2.88	863.0	42.0	1312.0
258	C	5584	3.23	8316.9	41.7	5582	3.32	3302.8	32.6	5014.1
259	A	5571	2.53	2317.4	77.2	5569	3.41	715.0	48.1	1602.4
260	U	5560	3.33	8089.3	112.9	5557	3.37	3359.2	46.4	4730.1
261	G	5548	3.48	2752.5	86.0	5547	3.15	1169.6	34.1	1582.9
262	C	5534	3.15	9546.3	140.3	5532	3.57	3219.6	23.5	6326.7
263	C	5522	3.23	14303.0	154.7	5519	3.03	3459.4	32.0	10843.6
264	U	5509	3.43	694.4	88.5	5506	2.43	0.0	30.7	694.4
265	C	5496	3.03	7750.9	60.9	5493	3.04	3240.6	43.9	4510.4
266	U	5484	2.54	2562.3	68.4	5481	2.43	1085.1	37.0	1477.1
267	C	5470	2.67	4241.6	103.8	5467	3.57	2245.8	44.1	1995.8
268	C	5456	3.15	14773.2	107.5	5453	3.32	7135.8	72.4	7637.4
269	U	5443	3.09	4198.8	84.2	5441	2.84	1877.0	41.4	2321.8
270	C	5431	3.06	6973.3	123.4	5428	3.32	2168.9	19.2	4804.4
271	A	5418	3.23	17745.6	170.6	5415	3.04	3394.5	40.9	14351.1
272	A	5405	3.28	21376.0	170.0	5402	2.80	2106.5	37.5	19269.5
273	C	5391	2.75	4359.4	108.2	5388	2.43	802.1	31.5	3557.4
274	C	5377	3.15	7009.5	65.0	5374	2.95	2067.6	44.3	4941.9
275	U	5365	3.10	12534.5	80.7	5363	3.18	5451.1	61.7	7083.3
276	G	5354	2.72	3767.3	71.2	5352	3.23	1558.3	55.2	2209.1
277	C	5340	2.54	3336.0	63.4	5338	2.43	1117.6	26.3	2218.4
278	U	5327	2.54	1894.0	109.5	5324	2.43	347.1	27.7	1546.9
279	U	5315	3.25	11008.3	82.4	5313	3.47	3118.3	49.0	7890.0
280	C	5301	3.24	15791.8	116.9	5299	3.43	5346.7	43.2	10445.1
281	A	5289	2.99	5362.7	105.4	5285	3.13	1585.5	48.1	3777.1
282	G	5279	3.02	3252.2	97.0	5276	2.77	1215.2	39.9	2037.0
283	U	5268	2.97	7112.4	83.9	5265	3.03	2948.5	31.6	4163.9
284	A	5255	2.54	2919.0	87.4	5253	2.43	914.8	26.6	2004.2
285	C	5242	3.54	3018.4	144.4	5239	3.63	923.2	44.3	2095.2
286	C	5228	3.16	20876.3	238.4	5226	3.29	6273.4	51.7	14602.9
287	A	5216	2.66	4502.5	90.2	5213	2.80	1359.6	26.0	3142.9
288	C	5202	2.59	1248.3	72.8	5199	1.95	172.0	13.1	1076.3

289	C	5188	2.79	7987.4	95.3	5186	2.50	1756.6	23.8	6230.8
290	U	5176	2.59	2108.8	67.4	5173	2.09	422.7	25.7	1686.1
291	C	5164	2.71	6940.8	119.6	5161	2.83	1569.0	33.4	5371.8
292	C	5150	2.94	13837.2	132.5	5147	2.83	3476.4	58.6	10360.8
293	A	5137	2.88	7751.7	148.8	5134	2.84	2351.8	68.2	5399.8
294	C	5122	3.39	15724.5	95.8	5119	3.59	4651.2	52.2	11073.4
295	A	5109	3.34	35517.8	164.3	5106	3.40	8013.9	113.4	27504.0
296	G	5097	3.35	15773.2	208.4	5094	2.66	1895.0	54.2	13878.2
297	A	5082	3.39	25141.4	198.7	5080	3.26	3694.3	37.4	21447.1
298	A	5068	3.39	21946.0	220.0	5065	3.23	3439.0	42.6	18507.0
299	U	5056	3.44	14333.0	265.0	5054	2.77	1451.2	30.5	12881.8
300	G	5046	2.59	3336.1	87.9	5045	2.17	854.2	33.3	2481.8
301	G	5036	3.33	4448.5	112.9	5035	3.95	1618.8	71.9	2829.7
302	G	5026	3.20	14518.5	116.2	5023	3.33	6339.7	117.3	8178.8
303	C	5013	3.15	10737.7	97.8	5010	2.80	2575.7	56.8	8162.0
304	C	5000	3.20	5324.6	49.4	4998	1.95	580.8	28.0	4743.8
305	G	4990	2.78	2739.6	50.3	4988	1.95	109.3	16.6	2630.2
306	U	4979	2.77	2450.1	37.2	4976	1.95	275.5	17.3	2174.7
307	A	4965	2.93	5144.6	58.4	4962	2.19	1119.4	15.2	4025.2
308	C	4949	2.59	2348.8	57.4	4946	1.95	215.1	18.0	2133.7
309	C	4936	3.68	5907.5	152.6	4934	3.00	1316.4	48.8	4591.2
310	C	4925	3.33	14161.3	338.7	4923	3.14	4342.2	57.8	9819.1
311	A	4912	2.96	8454.5	206.1	4909	3.09	2726.2	43.7	5728.3
312	C	4899	3.28	16593.2	124.3	4896	3.20	5724.4	31.4	10868.8
313	A	4886	3.38	5317.3	64.3	4884	3.15	1467.3	21.9	3849.9
314	G	4875	2.53	925.8	99.0	4873	2.25	310.3	27.1	615.5
315	C	4864	2.59	5176.2	54.8	4861	2.75	2188.4	39.0	2987.9
316	A	4851	3.23	7075.1	157.2	4847	3.15	2891.0	44.8	4184.0
317	G	4840	3.08	14984.8	266.7	4837	3.19	6207.6	55.5	8777.3
318	U	4829	3.15	30028.5	300.6	4826	3.19	6395.8	46.7	23632.7
319	G	4817	3.33	2830.4	164.9	4814	3.04	1322.5	38.6	1507.9
320	C	4804	2.65	6280.2	91.7	4801	2.80	2624.4	37.8	3655.8
321	A	4791	2.64	5044.2	83.3	4788	2.61	1717.1	25.7	3327.1
322	U	4779	3.15	5572.4	151.6	4777	3.15	2514.2	63.4	3058.1
323	G	4769	3.01	10352.9	243.7	4767	3.20	4866.7	46.6	5486.2
324	A	4758	2.53	2109.1	41.0	4755	2.00	913.4	39.7	1195.7
325	U	4748	2.53	497.8	50.9	4746	2.00	25.5	10.0	472.3
326	G	4738	2.94	4281.3	74.2	4735	3.06	892.4	38.7	3388.9
327	A	4726	2.96	11866.5	95.3	4723	2.82	2349.1	49.1	9517.4
328	C	4712	2.83	8367.7	151.9	4709	2.95	2858.5	61.5	5509.2
329	C	4699	3.16	18056.6	193.1	4695	3.11	5538.9	65.6	12517.7
330	C	4686	3.13	23447.7	201.0	4683	3.06	5427.4	66.3	18020.3
331	A	4675	3.08	14904.7	203.0	4671	2.28	1205.5	58.3	13699.2
332	A	4663	3.11	18703.5	170.1	4660	2.32	1953.1	37.9	16750.3
333	A	4651	2.98	21927.8	194.5	4648	2.14	1694.9	39.5	20232.9
334	A	4639	2.76	10341.7	164.5	4635	1.72	211.1	26.8	10130.6
335	C	4625	2.49	5415.8	151.7	4621	1.72	628.4	40.5	4787.4
336	C	4613	3.02	18374.8	216.7	4610	2.87	5041.7	54.8	13333.1

337	A	4601	2.98	20562.9	263.9	4598	2.48	2648.6	57.6	17914.3
338	A	4589	2.90	12434.6	167.1	4586	2.16	1169.1	30.9	11265.5
339	G	4578	2.83	7101.4	125.1	4574	1.72	96.6	28.3	7004.9
340	C	4566	2.60	7335.4	75.4	4563	2.34	2465.7	35.5	4869.7
341	C	4554	2.64	6920.2	89.5	4550	1.99	1404.7	45.6	5515.5
342	A	4542	2.52	5967.3	95.3	4538	1.72	483.1	25.2	5484.2
343	A	4529	2.56	5963.6	84.5	4525	1.88	656.7	24.7	5306.9
344	U	4518	2.76	9527.9	101.0	4515	2.27	1446.7	36.1	8081.2
345	C	4506	2.35	2646.7	145.8	4502	3.07	615.9	38.3	2030.8
346	C	4493	2.96	17628.3	170.7	4490	2.95	4216.9	37.9	13411.5
347	A	4481	2.85	9688.1	123.9	4477	2.54	1563.6	48.0	8124.4
348	U	4469	2.53	6650.2	129.5	4465	1.79	626.4	30.9	6023.8
349	C	4455	2.90	11586.9	168.7	4452	3.02	3513.3	67.0	8073.5
350	U	4443	2.98	20464.2	178.1	4440	3.14	5921.2	46.5	14543.0
351	G	4433	2.35	2036.9	112.1	4429	2.82	1161.3	25.4	875.6
352	G	4423	2.35	1082.6	70.8	4419	2.94	943.4	23.0	139.2
353	U	4411	2.65	5137.9	83.8	4408	2.77	2325.9	47.7	2812.0
354	U	4400	2.90	5061.0	108.5	4398	3.34	1900.8	111.0	3160.2
355	G	4390	2.35	5620.0	256.7	4387	3.55	5472.8	128.3	147.1
356	G	4381	3.30	34011.8	187.4	4377	3.59	18093.6	169.0	15918.2
357	U	4370	3.16	13968.9	242.6	4365	3.32	5060.6	210.8	8908.3
358	C	4357	2.82	13231.4	205.7	4353	2.94	4195.1	95.1	9036.3
359	A	4346	3.16	26584.2	326.1	4342	3.19	5575.6	51.0	21008.5
360	U	4335	3.30	21421.0	216.9	4331	2.96	4911.1	72.6	16509.8
361	U	4323	2.35	1813.7	143.6	4319	1.79	399.4	28.0	1414.3
362	U	4313	2.70	6061.6	103.7	4309	2.03	563.9	17.5	5497.6
363	U	4301	2.50	3929.6	115.0	4297	1.79	420.4	20.4	3509.2
364	A	4289	2.35	4805.2	81.1	4285	1.79	923.0	20.9	3882.2
365	C	4279	4.50	1493.2	264.0	4275	4.64	285.1	63.5	1208.0
366	G	4269	4.18	6230.1	130.2	4265	3.94	2665.3	28.8	3564.8
367	G	4260	2.97	5191.5	105.4	4255	2.50	924.4	57.6	4267.1
368	A	4249	2.37	1685.3	77.9	4245	1.79	31.9	22.9	1653.4
369	C	4237	2.83	6899.6	85.5	4234	2.39	1552.8	22.9	5346.8
370	A	4225	2.70	6657.6	85.3	4221	1.79	311.8	31.2	6345.8
371	C	4211	2.75	6444.7	112.4	4206	2.39	1170.5	30.3	5274.3
372	C	4199	2.69	3017.0	112.0	4194	1.79	218.6	46.5	2798.4
373	C	4187	2.82	9526.2	201.1	4182	2.84	2741.3	55.3	6784.8
374	A	4176	3.10	17504.7	130.1	4172	2.79	2623.9	40.1	14880.8
375	U	4165	2.90	11442.9	161.6	4161	3.21	2171.8	53.4	9271.1
376	C	4152	3.00	14569.7	204.4	4148	3.21	5717.7	71.7	8852.1
377	U	4141	3.18	26232.5	132.2	4138	3.41	9254.0	108.3	16978.5
378	A	4129	2.84	23559.9	282.3	4125	2.58	7008.7	181.9	16551.3
379	U	4119	3.22	30774.5	446.3	4116	3.48	11351.9	182.9	19422.6
380	G	4109	3.34	14233.9	497.9	4106	3.67	2513.4	129.0	11720.4
381	A	4099	2.37	6410.9	162.7	4095	2.08	1315.1	47.1	5095.8
382	U	4086	2.40	5522.0	124.5	4082	1.91	334.8	24.7	5187.2
383	U	4076	2.37	1811.7	117.3	4073	2.02	679.7	46.4	1132.0
384	C	4063	2.37	6031.4	214.7	4060	2.88	4055.9	56.1	1975.4

385	C	4052	2.37	3854.3	148.6	4052	2.43	1497.1	70.7	2357.3
386	G	4048	4.57	17098.6	329.2	4044	4.31	8413.3	113.9	8685.4
387	U	4035	4.57	2864.4	356.9	4032	4.31	957.5	128.3	1906.9
388	A	4024	2.61	9028.8	122.7	4020	2.80	2785.4	40.8	6243.4
389	U	4015	3.07	14542.3	203.2	4012	2.90	2325.8	48.3	12216.5
390	A	4004	3.12	2864.8	152.5	4001	1.74	0.0	43.6	2864.8
391	C	3992	2.68	5064.9	93.8	3989	2.76	2439.5	48.7	2625.4
392	A	3982	2.84	12547.3	155.3	3979	2.71	2342.8	61.7	10204.5
393	C	3969	2.37	3047.2	180.5	3965	2.17	1440.4	50.1	1606.9
394	C	3957	2.39	4068.9	190.1	3952	2.95	1750.0	28.5	2318.9
395	U	3947	2.97	5551.6	116.8	3942	2.95	886.1	31.2	4665.5
396	U	3937	2.99	12808.1	109.6	3933	2.71	2242.8	45.5	10565.3
397	A	3925	2.97	16068.4	183.9	3922	2.66	2193.3	42.6	13875.1
398	U	3915	2.95	9650.7	172.0	3910	2.11	1269.0	63.3	8381.7
399	C	3902	2.94	11339.6	168.2	3899	1.91	1229.6	42.5	10110.0
400	A	3892	3.03	10926.5	208.1	3888	1.74	0.0	31.3	10926.5
401	A	3880	3.16	16291.7	104.7	3876	1.74	1843.2	41.7	14448.5
402	A	3869	3.20	8447.6	80.5	3869	1.40	0.0	11.7	8447.6
403	U	3858	3.61	6631.7	192.8	3862	1.40	0.0	4.9	6631.7
404	G	3848	4.45	3658.0	98.9	3848	1.40	165.9	5.5	3492.1
405	U	3838	3.49	1326.1	132.3	3836	1.40	0.0	25.5	1326.1
406	C	3828	3.85	6428.3	64.9	3824	3.75	2918.4	75.9	3509.9
407	G	3818	2.85	2332.7	112.7	3813	4.44	971.5	75.1	1361.2
408	C	3807	2.00	348.6	38.8	3802	1.71	418.0	28.1	-69.4
409	C	3794	2.28	1200.6	35.5	3789	3.20	999.2	27.0	201.3
410	U	3784	3.23	4420.4	51.2	3779	3.06	2558.9	49.9	1861.5
411	A	3773	3.05	6937.3	65.9	3769	3.15	2987.5	50.1	3949.8
412	U	3765	3.35	2235.5	123.4	3760	3.75	951.1	38.1	1284.5
413	G	3756	3.28	3320.7	103.0	3753	3.60	1515.0	38.1	1805.7
414	U	3748	3.32	5707.5	93.7	3743	3.44	2121.0	26.5	3586.5
415	A	3737	3.42	6556.3	84.5	3732	3.55	2435.8	21.0	4120.5
416	C	3727	2.87	2125.2	87.1	3727	1.40	88.0	26.9	2037.1
417	U	3717	3.38	5483.9	108.2	3720	5.20	1815.2	70.5	3668.7
418	U	3707	3.42	11097.0	96.9	3704	5.20	3628.1	106.2	7468.9
419	U	3697	3.60	4987.7	62.2	3693	4.45	2110.7	74.9	2877.1
420	C	3686	2.93	1332.8	64.0	3682	3.64	1026.5	29.1	306.3
421	C	3676	3.30	5053.7	34.7	3671	3.73	3742.9	41.2	1310.8
422	A	3664	3.12	1194.3	45.4	3659	2.94	827.7	39.9	366.5
423	C	3651	2.00	280.1	13.4	3646	2.82	305.6	24.4	-25.5
424	C	3640	2.06	649.7	19.7	3640	1.40	3.3	22.6	646.4
425	U	3630	5.05	810.0	21.6	3633	4.80	938.0	61.4	-128.0
426	G	3618	4.27	802.6	58.5	3621	5.42	1237.6	62.8	-435.0

SHAPE_example_3_peaks.txt

seqnum	seq	RX.pos	RX.sigma	RX.area	RX.rms	BG.pos	BG.sigma	BG.area	BG.rms	(RX.area-BG.area)
6	G	9336	4.12	1832.5	54.8	9332	2.80	0.0	0.0	1832.5
7	A	9323	4.30	6934.4	70.2	9319	4.30	2598.7	37.7	4335.7
8	A	9306	3.95	482.2	87.4	9304	4.90	204.9	29.7	277.4
9	C	9289	4.70	7237.2	126.9	9285	4.75	2534.1	32.5	4703.1
10	U	9274	4.44	12909.8	153.2	9271	5.29	4937.0	65.1	7972.8
11	U	9256	3.95	130.2	89.4	9250	5.09	5.7	40.2	124.5
12	C	9240	4.90	11496.2	144.4	9235	4.22	2940.2	53.3	8555.9
13	U	9225	4.97	25924.5	238.6	9221	5.69	7175.6	84.6	18748.9
14	A	9209	4.95	13952.9	199.8	9204	4.94	2138.3	100.4	11814.6
15	G	9194	4.75	3354.0	126.5	9188	2.05	8.5	30.4	3345.5
16	U	9178	4.50	5542.7	75.1	9172	3.81	1466.2	35.6	4076.5
17	A	9162	4.34	5546.4	67.0	9158	4.30	2027.9	50.4	3518.5
18	U	9144	3.95	243.2	60.5	9138	5.25	187.6	40.8	55.7
19	A	9130	3.99	3250.4	74.9	9125	2.25	706.8	31.3	2543.6
20	U	9112	3.95	3260.3	89.3	9109	2.05	0.0	6.8	3260.3
21	U	9097	5.09	14482.9	300.7	9093	2.05	0.0	29.3	14482.9
22	C	9079	5.20	17757.9	192.4	9076	5.80	3460.7	162.1	14297.1
23	U	9064	5.11	21631.2	195.2	9060	6.70	7861.3	145.6	13769.9
24	G	9051	5.21	19856.4	176.7	9046	5.90	6799.5	59.6	13056.8
25	U	9036	5.30	10668.4	175.3	9032	6.70	5384.3	128.2	5284.1
26	A	9020	6.70	2656.4	125.9	9016	6.70	1965.9	94.0	690.6
27	U	9004	4.80	7726.0	163.6	9000	6.70	7262.1	104.3	464.0
28	A	8990	3.89	4727.7	83.6	8983	6.60	5273.7	143.6	-546.0
29	C	8972	2.70	43.6	81.2	8965	5.01	2622.3	77.7	-2578.6
30	C	8954	4.36	13167.6	99.2	8951	5.00	4660.6	48.5	8507.0
31	U	8938	4.80	10147.0	177.5	8934	4.35	1919.6	53.1	8227.3
32	A	8922	4.80	24476.4	188.4	8919	3.93	2234.0	44.9	22242.4
33	A	8906	4.85	13780.1	166.1	8901	2.55	452.2	35.6	13328.0
34	U	8890	4.95	15965.1	198.2	8887	3.36	2639.8	61.0	13325.3
35	A	8874	4.94	32994.4	241.1	8872	2.62	1515.0	47.5	31479.4
36	U	8858	4.95	19503.8	231.7	8856	2.55	0.0	35.6	19503.8
37	U	8843	4.99	38628.0	354.1	8839	2.55	963.6	30.3	37664.4
38	A	8826	4.90	16919.6	276.7	8822	2.70	1467.0	22.6	15452.6
39	U	8811	3.96	7099.4	86.9	8808	2.55	0.0	17.2	7099.4
40	A	8796	4.25	4265.8	76.9	8793	3.62	1195.9	27.7	3069.9
41	G	8780	3.19	1164.4	70.2	8777	4.16	612.2	17.4	552.2
42	C	8765	2.70	705.5	49.5	8759	2.55	199.1	19.2	506.4
43	C	8748	3.32	1873.8	50.5	8744	2.95	691.1	22.8	1182.8
44	U	8733	2.70	740.7	54.8	8732	2.95	94.7	13.5	646.0
45	U	8717	5.00	5113.1	220.7	8716	4.75	72.0	23.2	5041.0
46	U	8702	4.90	28767.7	244.0	8699	4.50	3508.8	37.8	25258.9
47	A	8686	4.89	23868.9	244.7	8683	4.41	3168.2	66.6	20700.8
48	U	8667	5.25	23519.3	288.2	8665	6.29	3925.6	214.8	19593.7

49	C	8651	5.45	45741.1	424.9	8647	6.08	14666.0	174.0	31075.0
50	A	8637	5.38	35217.9	408.6	8634	4.73	7460.3	117.1	27757.6
51	A	8621	5.09	26353.7	331.0	8618	5.45	6082.3	134.2	20271.4
52	C	8604	4.53	12641.4	131.4	8600	3.88	2345.3	55.3	10296.1
53	A	8587	4.90	18419.6	142.6	8584	3.91	2077.6	32.4	16342.1
54	A	8570	4.85	11250.0	152.3	8568	5.15	1668.7	65.0	9581.3
55	U	8555	4.87	14746.8	119.5	8552	5.58	3394.1	78.2	11352.7
56	G	8542	4.95	7478.5	89.0	8542	6.00	3056.3	72.6	4422.2
57	G	8528	4.41	2326.6	75.6	8526	6.40	968.3	48.7	1358.3
58	A	8511	5.60	1162.7	93.9	8512	4.25	663.2	19.4	499.4
59	A	8495	2.45	138.9	34.1	8495	1.80	333.6	17.1	-194.7
60	U	8479	4.05	379.9	50.6	8479	4.75	415.4	28.9	-35.4
61	C	8465	3.41	3571.3	99.7	8463	4.49	2137.6	25.1	1433.6
62	C	8447	3.35	2364.4	129.1	8447	4.16	1689.1	45.1	675.3
63	C	8433	4.90	13916.8	213.3	8431	5.40	6527.6	107.4	7389.3
64	A	8417	4.59	25200.7	257.3	8414	4.86	4703.8	109.1	20496.9
65	A	8402	4.44	17398.6	181.5	8399	4.18	3341.7	56.7	14056.9
66	C	8385	4.90	15975.7	203.2	8383	4.95	6707.7	76.9	9268.0
67	A	8369	4.90	27224.8	232.2	8366	5.15	4952.7	87.7	22272.1
68	A	8353	4.75	29680.0	180.4	8351	4.62	5761.4	44.1	23918.6
69	U	8338	4.65	13134.0	110.1	8336	5.10	1473.4	72.1	11660.6
70	U	8324	4.80	30900.0	205.5	8321	5.26	5641.7	79.3	25258.4
71	A	8308	4.88	14255.8	207.6	8306	3.61	1840.0	28.8	12415.8
72	U	8293	3.83	6310.1	73.9	8290	3.89	666.7	25.4	5643.4
73	C	8277	3.37	2870.8	77.7	8274	3.19	1293.7	21.0	1577.1
74	U	8261	2.63	1398.6	93.7	8258	2.19	410.5	26.0	988.1
75	C	8244	4.43	7066.5	96.7	8243	4.95	2143.4	52.5	4923.0
76	A	8230	4.48	19132.5	90.1	8227	4.58	2941.4	63.2	16191.1
77	A	8215	4.48	11891.5	69.8	8212	3.09	1226.9	28.5	10664.6
78	C	8198	3.70	4355.0	90.1	8196	4.14	1985.9	29.1	2369.2
79	A	8183	3.82	5448.6	67.3	8180	4.34	2399.8	26.8	3048.8
80	U	8166	4.01	1379.8	62.7	8165	3.89	733.5	29.0	646.3
81	U	8153	2.63	493.2	62.6	8150	2.19	58.2	17.6	435.0
82	C	8137	3.72	3687.5	55.4	8135	2.34	450.9	12.6	3236.6
83	A	8122	3.91	7522.1	85.1	8119	2.19	71.7	15.8	7450.4
84	C	8104	4.29	6386.9	116.3	8101	2.52	810.3	37.3	5576.6
85	C	8088	4.43	13938.7	137.5	8085	4.39	3233.7	46.6	10705.0
86	C	8072	4.43	23096.8	105.7	8070	4.58	6014.9	63.4	17081.9
87	A	8057	4.43	22943.7	124.3	8054	4.51	2243.9	73.7	20699.8
88	A	8042	4.15	7969.1	104.3	8039	2.82	1135.7	28.4	6833.4
89	U	8026	4.01	5571.7	108.0	8021	2.99	332.5	32.5	5239.3
90	U	8010	3.44	5561.5	147.1	8009	3.08	922.0	33.5	4639.4
91	C	7994	2.63	2126.2	68.9	7992	3.74	1614.0	20.7	512.2
92	U	7978	2.73	2048.7	60.9	7976	3.42	1228.8	32.5	819.8
93	C	7963	3.57	5417.4	145.4	7961	3.54	1479.9	40.4	3937.5
94	A	7946	4.28	14324.2	79.1	7943	4.24	2690.6	24.1	11633.6
95	U	7930	4.35	9721.5	82.4	7928	4.34	2900.2	29.6	6821.3
96	G	7915	3.15	1605.0	75.5	7914	3.57	752.1	25.8	853.0

97	G	7899	3.15	830.4	69.4	7897	3.60	342.9	22.6	487.5
98	U	7884	3.58	5071.9	87.1	7882	3.89	2452.4	27.5	2619.4
99	A	7867	3.55	3344.2	117.8	7864	3.91	1686.6	34.0	1657.5
100	G	7853	4.00	3.4	90.5	7851	3.29	0.0	27.4	3.4
101	C	7842	4.15	3970.6	74.4	7837	4.04	1008.3	20.7	2962.3
102	G	7828	3.91	3422.5	74.9	7823	3.29	323.4	27.0	3099.2
103	C	7810	4.00	4010.8	83.5	7808	3.93	1319.3	34.4	2691.5
104	C	7794	3.77	8454.5	124.9	7792	3.83	2288.0	37.7	6166.5
105	U	7778	3.15	2117.1	71.3	7777	3.29	868.0	31.4	1249.1
106	G	7764	3.15	1404.4	97.7	7764	4.54	309.9	38.1	1094.4
107	U	7750	3.96	12595.8	84.5	7747	4.14	3084.6	39.2	9511.2
108	G	7737	3.90	6023.7	87.0	7734	4.29	2128.2	38.2	3895.5
109	C	7721	3.35	4625.9	105.2	7718	4.00	2434.3	45.5	2191.6
110	U	7706	3.52	4086.9	104.4	7703	3.96	2073.3	49.8	2013.6
111	U	7691	3.90	11399.1	113.7	7687	4.10	2121.2	40.6	9278.0
112	C	7674	3.96	11825.0	150.1	7671	3.99	3216.2	33.0	8608.8
113	G	7660	4.00	3939.6	110.5	7657	3.58	1296.8	45.4	2642.8
114	G	7643	4.35	1514.1	135.0	7640	3.29	767.7	55.7	746.3
115	U	7628	4.15	16869.0	146.2	7625	4.57	3736.2	41.7	13132.8
116	U	7614	4.05	19381.0	91.6	7611	4.28	5453.1	46.1	13927.9
117	A	7600	3.15	397.5	78.5	7595	3.29	266.1	26.5	131.4
118	C	7586	3.15	1261.7	60.2	7583	4.14	1123.5	23.4	138.2
119	U	7573	3.45	3842.9	66.2	7570	4.24	2323.7	20.5	1519.2
120	U	7557	2.90	1336.8	60.3	7555	3.82	934.1	19.2	402.7
121	C	7541	2.90	240.9	48.4	7536	4.76	385.6	33.9	-144.7
122	U	7526	4.66	8007.5	220.9	7522	4.63	2566.1	39.9	5441.4
123	A	7509	4.45	22171.4	280.9	7507	4.48	4515.9	30.0	17655.6
124	A	7494	4.13	40854.9	237.8	7492	4.18	3377.7	36.6	37477.3
125	G	7481	3.98	11633.8	110.1	7478	4.43	912.6	44.4	10721.2
126	G	7468	4.13	12670.2	118.6	7465	4.53	3721.5	36.0	8948.7
127	A	7454	4.28	3773.5	92.3	7450	3.33	1133.9	39.8	2639.7
128	A	7439	4.30	948.4	73.9	7436	3.33	228.3	36.3	720.1
129	G	7424	4.00	6360.3	56.2	7422	3.95	2220.4	25.4	4139.9
130	U	7410	3.40	1817.0	63.9	7409	3.33	236.8	22.0	1580.2
131	C	7395	2.90	1425.6	55.4	7390	3.33	380.4	16.6	1045.2
132	C	7379	3.14	4352.7	58.3	7377	3.33	1992.6	23.5	2360.1
133	A	7364	2.90	921.8	68.8	7360	3.33	365.8	26.2	556.0
134	C	7348	3.20	5299.3	76.3	7345	3.88	3057.2	22.9	2242.2
135	A	7332	3.26	3136.3	56.5	7329	3.73	1570.5	20.7	1565.8
136	C	7316	3.44	6639.6	58.7	7314	3.90	2833.0	25.5	3806.6
137	A	7302	3.66	8039.0	120.2	7298	3.99	1735.0	36.0	6304.0
138	A	7287	4.13	19936.2	194.4	7284	4.09	4236.3	42.8	15699.8
139	A	7271	4.13	28646.6	191.7	7269	4.18	4583.3	36.9	24063.3
140	U	7256	3.63	7519.9	75.2	7252	4.33	1301.8	41.4	6218.1
141	C	7241	4.05	18962.1	101.7	7238	4.58	5551.1	53.6	13411.0
142	A	7226	4.08	41188.9	140.0	7223	4.00	8574.8	89.8	32614.1
143	A	7211	4.08	23661.5	150.3	7209	3.49	2231.1	28.4	21430.4
144	G	7197	3.24	4841.5	124.1	7196	3.58	192.0	20.3	4649.5

145	A	7181	2.70	3102.5	90.6	7176	2.16	164.0	18.1	2938.4
146	U	7166	2.88	5572.4	89.2	7163	2.36	969.5	24.0	4602.9
147	C	7152	2.70	2322.5	88.2	7149	2.16	531.5	36.0	1791.1
148	C	7137	3.13	5246.5	75.4	7134	2.91	1686.8	28.7	3559.7
149	G	7122	2.70	3460.1	78.0	7120	3.91	474.3	57.4	2985.8
150	U	7109	4.11	22843.9	207.8	7106	3.92	4121.8	41.7	18722.1
151	U	7094	4.01	18671.5	197.2	7092	3.24	1053.8	28.5	17617.8
152	A	7079	3.59	12671.6	75.4	7077	4.51	169.7	36.2	12501.9
153	G	7066	4.01	32406.1	212.3	7063	3.77	4005.4	54.2	28400.7
154	A	7051	4.13	17253.9	187.4	7049	2.22	834.1	33.8	16419.9
155	C	7035	3.62	10718.9	113.5	7033	3.96	2855.2	35.8	7863.7
156	G	7022	3.53	4703.9	55.0	7019	3.96	1434.4	25.8	3269.5
157	U	7008	3.45	5237.9	60.7	7005	3.91	1169.4	25.9	4068.5
158	U	6995	3.78	8841.2	126.5	6992	3.82	2217.6	27.0	6623.7
159	U	6979	4.18	1824.0	88.2	6977	4.25	160.3	39.6	1663.7
160	C	6964	3.96	22989.9	103.9	6962	3.96	5357.3	51.5	17632.5
161	A	6950	3.87	14097.4	71.1	6949	3.73	2337.8	40.5	11759.6
162	G	6935	2.70	1315.7	66.3	6935	4.31	48.6	18.1	1267.1
163	C	6923	2.82	781.3	53.4	6919	2.16	98.6	11.4	682.7
164	U	6909	2.70	1294.5	60.4	6905	2.16	472.4	25.6	822.1
165	U	6894	2.70	2240.2	56.0	6891	3.11	816.4	28.5	1423.8
166	C	6879	3.20	3772.1	76.6	6876	3.92	1896.2	35.6	1875.9
167	C	6864	3.48	7594.4	89.3	6861	3.91	3623.0	31.6	3971.4
168	A	6850	3.58	7244.0	80.5	6847	3.70	2331.3	25.8	4912.7
169	A	6835	3.93	7151.8	101.5	6832	3.86	1715.4	25.7	5436.4
170	A	6820	3.98	17288.2	110.6	6817	3.49	1583.1	37.4	15705.1
171	A	6805	3.81	27270.7	144.2	6802	3.45	2207.8	37.2	25062.8
172	C	6789	3.77	16173.1	109.4	6787	3.73	4303.5	43.8	11869.6
173	A	6774	3.77	11421.5	89.5	6772	3.96	2405.3	47.8	9016.2
174	G	6761	3.81	14787.8	92.1	6758	3.92	4056.0	61.2	10731.8
175	A	6746	3.78	16305.1	135.9	6744	3.77	3330.5	45.9	12974.6
176	A	6732	3.73	18426.7	128.6	6730	3.78	2006.9	34.7	16419.8
177	G	6718	2.58	898.4	84.2	6714	3.11	183.9	28.8	714.5
178	A	6703	3.01	5424.4	78.5	6701	3.11	2256.8	27.5	3167.5
179	A	6688	2.58	2882.8	71.4	6686	3.11	1011.8	36.2	1871.0
180	U	6674	2.58	2910.2	82.9	6670	3.35	1471.8	38.7	1438.4
181	G	6661	2.58	267.3	48.4	6657	3.11	213.7	38.6	53.6
182	U	6646	3.77	5451.9	69.1	6644	4.19	2843.3	43.3	2608.6
183	G	6633	3.67	6872.7	84.5	6631	4.15	3437.1	36.9	3435.7
184	A	6618	3.43	2600.0	58.2	6615	3.59	1356.4	29.3	1243.7
185	G	6605	3.77	10190.6	63.7	6603	3.91	3827.3	40.1	6363.3
186	A	6590	3.73	8086.7	98.8	6588	3.86	1773.4	33.1	6313.3
187	A	6576	3.68	10173.0	104.8	6573	3.67	1837.3	23.4	8335.7
188	G	6564	3.88	3705.0	72.2	6561	4.19	1847.9	28.3	1857.1
189	G	6552	3.06	1627.2	60.1	6547	4.83	545.6	32.2	1081.6
190	C	6537	2.95	379.8	44.3	6534	2.79	165.1	24.6	214.7
191	U	6524	3.45	4086.9	64.3	6520	3.90	2031.9	36.0	2055.0
192	U	6511	3.55	323.6	61.6	6506	2.79	81.1	23.5	242.5

193	C	6496	2.95	1747.2	70.1	6492	4.16	1040.9	23.1	706.3
194	C	6481	3.88	11259.0	123.5	6478	4.11	5149.5	39.0	6109.5
195	A	6465	3.67	8675.0	161.2	6463	4.14	3165.9	31.0	5509.1
196	C	6451	3.54	9171.9	134.8	6448	3.98	2954.1	33.0	6217.8
197	U	6436	3.75	10045.6	107.3	6434	3.79	3357.3	30.6	6688.3
198	A	6422	3.10	4642.9	94.8	6421	3.89	1073.2	37.5	3569.7
199	A	6407	3.50	7666.2	103.9	6405	4.03	3526.6	37.3	4139.6
200	G	6394	3.33	4318.9	105.2	6392	3.93	2983.9	41.2	1335.0
201	G	6383	3.12	5764.3	63.4	6381	3.79	3498.7	34.3	2265.6
202	C	6369	2.95	4.5	55.3	6367	4.27	83.0	38.6	-78.5
203	U	6356	3.67	14012.2	90.0	6353	4.19	5452.6	42.9	8559.6
204	A	6343	3.70	12076.2	124.8	6340	2.99	1178.0	54.2	10898.3
205	A	6329	3.70	15315.1	112.9	6326	2.79	1142.5	25.3	14172.6
206	C	6314	3.20	6843.5	112.3	6312	2.94	1445.9	26.0	5397.6
207	U	6301	2.95	1049.1	71.5	6298	3.60	277.3	32.5	771.7
208	C	6287	3.19	5952.3	77.0	6284	3.60	3005.6	27.9	2946.7
209	U	6273	3.18	3355.6	58.6	6270	3.89	1455.2	41.0	1900.4
210	C	6259	3.50	11376.7	56.2	6256	3.94	3936.4	45.5	7440.2
211	A	6245	3.25	11439.2	108.9	6242	3.10	2695.0	47.3	8744.2
212	A	6231	3.30	11313.3	118.6	6228	3.79	2038.0	61.0	9275.3
213	C	6216	3.50	11670.4	132.8	6213	4.34	3899.4	57.6	7771.0
214	A	6202	3.62	14097.6	88.3	6197	3.88	2147.1	105.8	11950.5
215	G	6190	3.58	23606.0	91.0	6188	3.69	4416.4	62.4	19189.6
216	A	6176	3.06	5954.9	181.7	6173	2.89	817.9	35.4	5136.9
217	C	6162	3.30	13840.5	206.3	6159	4.09	5493.2	47.8	8347.3
218	A	6148	3.58	38850.8	145.4	6145	3.79	7743.8	89.2	31107.0
219	A	6134	3.06	5985.0	164.9	6130	3.82	979.9	87.0	5005.1
220	C	6119	3.41	22983.7	138.1	6116	3.94	8286.1	84.4	14697.6
221	A	6106	3.46	28075.6	217.4	6103	3.45	5540.5	40.1	22535.0
222	A	6093	3.06	2828.9	241.9	6089	3.74	239.4	59.9	2589.5
223	C	6078	3.06	13292.4	155.4	6075	3.49	4691.9	77.6	8600.5
224	A	6063	3.06	10704.7	174.6	6061	2.59	1857.6	42.8	8847.1
225	C	6048	3.06	2164.7	179.7	6045	2.46	994.8	62.5	1169.9
226	C	6033	3.55	12300.5	126.2	6030	3.94	5783.9	53.9	6516.6
227	U	6021	3.86	15865.9	196.4	6018	3.94	4933.2	52.1	10932.8
228	G	6009	4.00	2556.2	209.8	6006	4.09	330.3	58.8	2225.9
229	C	5997	4.01	7285.8	163.1	5994	3.00	1752.3	31.7	5533.5
230	U	5984	3.68	29547.3	365.8	5982	3.74	4210.8	38.0	25336.5
231	U	5972	3.42	15668.6	167.0	5968	3.58	1593.1	57.5	14075.5
232	C	5957	3.37	13562.6	175.4	5955	3.15	3394.3	44.1	10168.3
233	A	5943	3.24	13131.3	127.0	5940	2.63	1889.8	48.7	11241.5
234	U	5929	3.36	14613.7	100.7	5925	2.46	1199.3	50.4	13414.4
235	C	5914	3.41	13766.5	112.4	5911	3.19	3658.4	41.6	10108.1
236	A	5898	3.36	10949.4	100.2	5895	2.90	2420.1	44.4	8529.3
237	G	5885	3.22	9208.7	81.3	5883	3.09	2336.8	47.4	6871.9
238	C	5873	2.88	4717.3	92.6	5870	2.95	1644.3	42.8	3073.0
239	U	5860	2.54	1109.8	79.2	5856	2.29	242.5	15.8	867.4
240	G	5850	2.91	946.0	75.2	5848	2.09	334.1	19.4	612.0

241	U	5838	2.65	4676.4	84.7	5837	2.29	689.8	27.9	3986.5
242	U	5825	3.18	4779.4	135.5	5823	2.36	592.4	27.0	4187.0
243	C	5810	3.27	11905.6	133.1	5807	3.44	2002.7	25.8	9902.8
244	C	5796	3.55	17800.0	215.6	5793	3.70	5608.4	49.3	12191.6
245	A	5782	3.61	27462.6	137.0	5779	3.55	5828.7	77.6	21633.9
246	G	5771	3.62	23104.8	103.2	5769	3.79	6706.8	103.4	16398.0
247	A	5758	3.61	7510.9	113.5	5756	3.99	2535.1	58.8	4975.8
248	G	5748	3.46	9942.5	92.4	5745	3.19	1992.4	44.5	7950.1
249	A	5736	3.42	20116.8	107.0	5734	3.23	2851.7	42.4	17265.2
250	A	5722	2.42	1408.8	113.2	5720	2.09	199.2	35.7	1209.6
251	C	5708	2.42	3569.3	67.8	5706	3.07	1228.1	33.1	2341.2
252	C	5694	2.67	3973.4	105.5	5691	3.80	2923.6	39.0	1049.7
253	C	5679	3.12	8042.0	72.0	5676	3.84	4876.1	53.2	3165.9
254	C	5665	3.18	4729.8	100.0	5662	3.64	2734.8	44.3	1995.0
255	C	5651	3.04	9809.9	143.1	5649	3.61	5284.0	38.5	4525.9
256	A	5638	2.78	4589.3	73.0	5635	3.48	2879.0	52.3	1710.3
257	U	5625	2.42	507.7	75.8	5623	2.09	413.6	51.7	94.1
258	C	5611	2.91	7492.8	110.9	5609	3.44	4012.0	35.2	3480.8
259	A	5599	2.67	960.6	56.6	5594	3.25	906.2	30.0	54.5
260	U	5587	3.27	4983.9	76.8	5584	3.12	2664.0	30.4	2319.9
261	G	5575	2.67	1586.6	88.8	5573	2.77	963.0	37.0	623.6
262	C	5561	3.24	8867.7	95.6	5559	3.59	3137.8	35.0	5729.9
263	C	5549	3.29	13765.6	95.8	5546	3.50	3309.6	30.6	10456.0
264	U	5536	3.42	451.8	70.9	5533	3.45	230.0	30.7	221.7
265	C	5523	2.87	4786.9	54.3	5520	3.24	2442.8	39.4	2344.1
266	U	5511	2.67	1468.2	59.4	5508	2.77	631.4	22.7	836.8
267	C	5497	2.67	2457.1	68.5	5494	3.20	1492.8	32.2	964.3
268	C	5483	3.29	9181.5	113.8	5480	3.59	4273.8	41.7	4907.8
269	U	5469	3.39	3501.4	80.4	5466	3.69	1925.1	28.3	1576.3
270	C	5457	3.48	5549.9	82.0	5454	3.43	2653.2	39.2	2896.7
271	A	5444	3.48	23180.1	73.1	5442	3.52	3914.4	46.8	19265.7
272	A	5431	3.42	28473.1	119.2	5428	3.22	2099.0	55.5	26374.0
273	C	5417	2.79	4502.3	83.6	5414	2.84	1257.9	44.2	3244.4
274	C	5403	3.29	6794.2	85.2	5400	3.24	3394.6	48.8	3399.6
275	U	5391	3.14	9451.3	112.2	5389	3.37	4847.6	66.0	4603.7
276	G	5380	2.67	2566.5	67.0	5377	3.47	1516.7	49.4	1049.8
277	C	5366	2.67	2660.3	54.9	5364	2.77	1351.1	25.5	1309.2
278	U	5353	2.67	1082.3	75.4	5351	2.77	403.6	32.0	678.6
279	U	5341	3.47	10475.2	47.9	5338	3.77	2794.8	37.4	7680.4
280	C	5327	3.36	16534.5	85.8	5324	3.39	6369.7	62.8	10164.8
281	A	5315	3.27	4505.9	85.0	5311	3.32	1928.5	32.1	2577.4
282	G	5304	2.82	1796.9	77.8	5300	2.95	974.1	40.4	822.8
283	U	5294	3.17	6278.1	97.2	5291	2.94	2841.5	38.9	3436.7
284	A	5281	2.82	1925.3	96.6	5278	2.72	751.9	27.5	1173.4
285	C	5267	3.01	2313.7	85.9	5264	3.87	1327.9	45.8	985.8
286	C	5254	3.37	21540.5	104.4	5251	3.47	8082.5	121.0	13458.0
287	A	5242	2.88	4915.4	77.4	5239	2.72	1638.2	45.9	3277.2
288	C	5227	2.82	1291.4	76.0	5225	2.72	421.0	26.1	870.5

289	C	5214	2.82	7064.7	83.5	5211	2.72	2425.9	36.0	4638.7
290	U	5202	2.82	1527.0	79.0	5198	2.72	687.3	42.2	839.7
291	C	5189	2.90	6397.8	131.4	5186	3.03	1706.4	50.9	4691.4
292	C	5175	3.12	12968.8	176.4	5173	3.20	5422.1	47.8	7546.7
293	A	5162	2.82	5404.9	202.9	5159	3.47	2369.9	53.6	3035.1
294	C	5147	3.67	16583.4	144.0	5144	3.53	5236.4	59.7	11347.0
295	A	5134	3.52	42229.7	348.9	5132	3.57	8348.4	88.3	33881.3
296	G	5122	3.37	16956.3	281.7	5119	3.72	2115.7	53.3	14840.6
297	A	5107	3.57	32926.5	202.9	5104	3.53	4069.3	50.3	28857.2
298	A	5093	3.39	26319.5	161.0	5090	3.42	3145.9	33.7	23173.7
299	U	5082	3.37	15639.4	102.4	5078	3.27	1384.7	39.2	14254.7
300	G	5072	3.37	3166.1	77.4	5068	2.72	1315.8	34.1	1850.3
301	G	5061	3.37	2566.5	55.9	5058	2.87	1696.3	46.7	870.2
302	G	5051	3.25	7947.9	73.1	5048	3.34	4350.7	29.6	3597.1
303	C	5038	3.24	10176.3	83.5	5035	3.47	3592.4	37.3	6583.9
304	C	5025	3.42	4821.4	66.4	5021	3.23	2078.8	54.4	2742.6
305	G	5015	3.07	2685.4	48.5	5013	2.61	1076.4	38.9	1609.0
306	U	5003	2.56	1865.0	55.6	5000	2.40	658.5	34.9	1206.5
307	A	4989	2.31	2772.0	60.2	4986	2.42	1072.0	21.0	1700.0
308	C	4974	2.07	1340.5	48.4	4970	2.12	191.6	30.1	1148.9
309	C	4962	2.83	3977.1	166.2	4959	2.79	2142.3	102.4	1834.8
310	C	4951	3.27	13652.5	119.1	4948	3.52	6114.7	84.8	7537.8
311	A	4937	3.19	7925.6	99.7	4933	3.24	2364.4	39.2	5561.1
312	C	4923	3.26	15830.6	125.3	4920	3.32	5816.1	49.9	10014.5
313	A	4911	2.90	2916.2	106.0	4908	2.71	1509.3	41.0	1406.9
314	G	4900	2.07	728.0	43.3	4898	2.12	342.4	26.9	385.6
315	C	4889	2.50	3814.3	74.5	4885	2.74	2019.4	33.6	1795.0
316	A	4876	2.93	4543.3	137.3	4872	2.97	2649.4	51.7	1894.0
317	G	4865	3.47	11939.6	75.4	4861	3.22	3236.9	37.5	8702.6
318	U	4853	3.30	33194.7	191.4	4850	3.12	4890.0	49.0	28304.7
319	G	4841	2.07	1749.3	198.1	4838	2.30	736.5	29.9	1012.8
320	C	4829	2.70	5487.0	119.7	4826	3.14	3637.6	63.2	1849.3
321	A	4816	2.55	4021.6	113.0	4813	2.87	2121.0	74.5	1900.6
322	U	4805	3.19	3244.4	159.0	4802	3.54	2250.9	81.5	993.5
323	G	4794	3.20	9648.9	169.8	4791	3.59	4736.9	50.2	4912.0
324	A	4783	2.07	1315.1	69.4	4779	2.40	1000.2	35.8	314.9
325	U	4773	2.07	0.0	47.4	4767	2.12	279.4	21.3	-279.4
326	G	4762	3.00	2685.3	39.8	4759	2.70	1109.4	32.1	1575.9
327	A	4750	2.73	8895.9	139.9	4747	3.01	2016.4	36.4	6879.5
328	C	4736	2.66	5286.3	188.9	4733	3.52	3643.0	70.7	1643.4
329	C	4723	3.16	16750.2	144.3	4719	3.44	7723.9	90.9	9026.3
330	C	4710	3.16	22707.2	128.6	4707	3.28	8731.1	63.9	13976.1
331	A	4699	3.16	12947.2	127.8	4695	3.24	2505.3	55.4	10441.9
332	A	4687	3.16	18339.6	116.5	4683	2.86	2472.7	47.2	15866.8
333	A	4675	3.10	25614.5	225.6	4672	2.47	2355.9	40.1	23258.6
334	A	4663	2.56	9728.4	178.2	4659	2.24	675.3	34.5	9053.2
335	C	4649	2.30	3659.8	153.8	4646	2.89	1301.4	65.5	2358.4
336	C	4637	3.11	17499.7	157.9	4634	3.34	6993.8	73.3	10505.8

337	A	4625	3.05	22609.2	222.6	4622	3.24	4305.1	59.2	18304.0
338	A	4613	2.92	13430.3	195.1	4609	2.54	1835.6	50.5	11594.7
339	G	4602	2.60	5270.4	161.5	4596	2.24	455.3	40.6	4815.1
340	C	4590	2.52	5457.2	104.6	4586	3.05	3211.4	36.1	2245.7
341	C	4577	2.45	5064.0	113.9	4574	3.05	2734.3	48.4	2329.7
342	A	4566	2.30	4773.2	93.7	4562	2.64	1777.9	35.6	2995.3
343	A	4553	2.66	6293.1	166.3	4549	2.62	823.3	30.3	5469.9
344	U	4541	2.78	11781.1	197.6	4538	2.64	1325.9	30.1	10455.1
345	C	4530	2.30	1768.5	270.7	4525	3.44	671.0	51.4	1097.5
346	C	4516	3.05	21574.9	289.6	4513	3.05	5428.7	91.6	16146.1
347	A	4504	2.71	8459.7	174.0	4501	2.35	1666.4	39.3	6793.3
348	U	4492	2.42	5788.1	204.0	4489	2.24	244.8	48.5	5543.3
349	C	4478	3.19	11932.6	183.1	4474	2.96	3120.9	55.3	8811.6
350	U	4466	3.10	18887.1	251.4	4463	2.96	4417.6	50.9	14469.6
351	G	4456	2.44	1274.7	52.1	4453	2.50	484.0	30.6	790.6
352	G	4445	2.44	510.1	41.1	4443	2.15	294.9	24.5	215.2
353	U	4435	2.82	3921.1	60.8	4430	2.15	1249.7	38.6	2671.4
354	U	4423	3.04	3546.8	47.7	4420	2.62	1105.4	81.0	2441.4
355	G	4413	2.82	3163.0	89.3	4410	3.50	3466.6	42.9	-303.6
356	G	4404	3.20	9912.5	112.4	4400	3.25	6448.0	79.1	3464.4
357	U	4393	2.89	7779.5	145.5	4390	3.15	2220.6	70.0	5558.9
358	C	4380	3.44	16696.7	219.7	4376	3.30	5533.3	64.3	11163.4
359	A	4368	3.25	31787.6	416.7	4365	3.30	5344.3	66.7	26443.2
360	U	4358	2.92	16952.1	225.4	4354	3.34	4121.0	47.9	12831.1
361	U	4346	3.06	1173.2	83.0	4343	2.15	198.8	34.1	974.4
362	U	4335	2.92	6206.6	88.5	4331	2.15	451.1	14.7	5755.6
363	U	4324	2.61	4012.0	73.5	4320	2.51	948.8	23.0	3063.3
364	A	4312	2.44	2966.1	59.8	4307	2.64	1671.8	43.3	1294.3
365	C	4301	4.64	834.9	170.0	4297	2.85	1102.5	79.9	-267.6
366	G	4291	3.63	5109.7	112.7	4288	4.50	3815.4	56.3	1294.4
367	G	4282	2.44	3145.7	114.6	4277	2.80	1547.5	85.5	1598.2
368	A	4272	2.44	258.0	103.0	4267	2.15	188.1	43.1	69.9
369	C	4259	2.68	4863.0	117.5	4256	2.77	2217.2	47.9	2645.8
370	A	4248	2.44	6194.4	112.5	4243	2.15	161.8	26.0	6032.6
371	C	4234	2.78	6095.7	199.9	4229	2.37	1561.4	29.7	4534.3
372	C	4222	2.44	2467.5	147.4	4217	2.58	805.4	45.8	1662.1
373	C	4209	3.08	11102.5	125.9	4205	3.09	4742.5	43.8	6360.0
374	A	4198	3.18	18742.1	125.9	4194	3.17	3329.4	35.3	15412.7
375	U	4187	2.95	9860.6	145.8	4182	3.42	2208.5	54.1	7652.1
376	C	4174	3.18	14382.7	146.8	4169	3.22	6866.6	101.2	7516.1
377	U	4163	3.33	26723.9	137.7	4159	3.23	11090.0	111.8	15633.9
378	A	4151	3.18	25005.0	272.4	4147	3.10	6873.8	156.8	18131.2
379	U	4141	3.33	28728.5	360.4	4138	3.39	11490.8	198.3	17237.7
380	G	4131	3.37	17536.5	423.3	4128	3.56	3385.3	116.0	14151.3
381	A	4121	2.30	5288.3	149.5	4117	2.42	1137.5	21.3	4150.8
382	U	4108	2.93	6338.0	98.7	4105	2.42	160.2	15.3	6177.8
383	U	4098	2.23	2260.6	57.8	4094	2.42	526.1	25.4	1734.5
384	C	4085	2.38	2772.9	65.0	4082	3.02	2308.9	52.8	464.1

385	C	4076	2.23	1433.5	130.2	4073	4.04	1544.0	51.4	-110.5
386	G	4069	4.08	8536.9	93.0	4066	4.37	6161.0	66.2	2375.9
387	U	4056	3.63	3503.2	165.9	4052	4.57	1342.9	93.7	2160.4
388	A	4045	3.09	11216.8	123.8	4041	2.82	2441.1	67.1	8775.7
389	U	4036	3.09	15421.9	138.6	4033	2.77	2339.3	45.1	13082.6
390	A	4025	2.23	1583.3	127.5	4022	2.42	0.0	32.4	1583.3
391	C	4013	3.58	5242.5	193.8	4010	3.02	3451.1	45.9	1791.4
392	A	4002	3.04	12449.6	427.7	4000	3.02	2085.7	44.5	10364.0
393	C	3989	2.23	1324.7	98.2	3985	2.94	1459.8	34.5	-135.1
394	C	3977	2.66	4158.8	82.6	3973	3.04	2306.5	33.6	1852.3
395	U	3967	3.51	4444.6	170.0	3963	2.76	1294.6	41.0	3150.0
396	U	3957	3.15	13962.6	312.6	3954	3.22	3362.2	33.8	10600.4
397	A	3946	2.94	15477.7	228.9	3942	3.21	3511.0	46.1	11966.8
398	U	3936	3.01	11693.5	199.8	3931	3.18	2993.4	46.8	8700.1
399	C	3923	3.31	11952.7	74.3	3919	3.26	3991.8	40.7	7960.9
400	A	3912	3.34	12372.6	136.1	3908	2.92	2034.2	40.3	10338.4
401	A	3900	3.19	19093.5	262.5	3897	3.28	4763.6	53.6	14330.0
402	A	3890	2.80	6178.7	134.0	3886	3.26	1048.4	56.7	5130.3
403	U	3879	3.57	7110.6	152.7	3877	3.19	1147.0	41.9	5963.7
404	G	3869	4.21	2999.4	95.9	3868	3.81	1750.7	31.0	1248.7
405	U	3858	3.31	623.7	89.2	3856	2.66	593.1	34.5	30.6
406	C	3848	3.72	6001.9	72.1	3844	4.28	5482.2	67.4	519.7
407	G	3838	2.73	1578.2	93.5	3833	3.14	1319.1	72.5	259.1
408	C	3827	1.96	499.0	31.3	3821	2.66	445.8	34.5	53.2
409	C	3815	2.80	1323.3	53.9	3809	2.79	1344.7	37.2	-21.4
410	U	3804	3.36	4053.0	47.9	3799	3.21	2888.9	38.6	1164.1
411	A	3793	3.43	5856.7	64.8	3788	3.61	3135.9	43.6	2720.8
412	U	3780	4.30	2640.3	115.5	3772	3.89	1688.4	37.8	951.9
413	G	3767	3.91	5671.4	139.6	3762	3.18	2025.9	39.2	3645.5
414	U	3756	3.17	5575.3	78.2	3756	2.66	678.9	29.3	4896.4
415	A	3746	3.16	1814.9	85.0	3750	3.02	1759.7	36.5	55.2
416	C	3735	3.53	5880.7	63.7	3738	5.05	1575.7	82.7	4305.0
417	U	3725	3.38	12469.1	95.4	3721	4.46	3597.5	88.0	8871.5
418	U	3715	3.11	3292.9	88.8	3711	3.03	2163.7	37.9	1129.2
419	U	3705	3.06	1103.2	48.4	3700	3.66	1262.8	56.2	-159.6
420	C	3695	3.42	5001.8	56.0	3689	4.11	5271.3	94.1	-269.5

DMS_example_1_peaks.txt

seqnum	seq	RX.pos	RX.sigma	RX.area	RX.rms	BG.pos	BG.sigma	BG.area	BG.rms	(RX.area-BG.area)
7	A	9611	5.21	31148.4	254.3	9607	4.32	1782.2	44.7	29366.2
8	A	9595	5.02	36816.6	342.6	9589	5.81	2.4	65.8	36814.2
9	C	9578	5.25	41562.2	344.4	9571	5.27	3445.1	72.5	38117.1
10	U	9561	5.26	8503.4	320.3	9557	4.77	3268.8	33.6	5234.6
11	U	9543	3.40	421.1	177.1	9539	3.52	116.4	41.7	304.7
12	C	9526	5.34	27302.7	264.2	9521	5.00	4592.8	61.2	22710.0
13	U	9508	5.30	14934.0	166.5	9505	5.30	5620.0	54.0	9314.0
14	A	9491	5.02	31481.2	347.5	9487	5.70	2968.6	72.8	28512.5
15	G	9476	3.40	1985.4	82.9	9467	5.42	621.5	59.1	1363.9
16	U	9460	4.78	3640.6	75.4	9455	4.60	1793.0	35.6	1847.6
17	A	9443	4.48	7155.8	90.2	9438	4.29	1891.2	30.6	5264.7
18	U	9426	3.40	707.3	78.1	9423	3.50	160.9	21.3	546.4
19	A	9409	3.95	5609.6	90.1	9405	4.35	1985.5	36.8	3624.1
20	U	9393	3.40	642.3	46.3	9389	3.50	574.0	41.6	68.2
21	U	9375	3.67	3033.4	107.8	9372	3.50	631.6	40.8	2401.9
22	C	9358	4.45	16979.6	131.0	9353	4.70	3220.5	40.2	13759.1
23	U	9342	4.65	8152.2	109.2	9338	4.54	4704.0	40.2	3448.2
24	G	9327	4.55	4530.3	109.3	9325	5.20	2609.0	93.4	1921.3
25	U	9311	3.40	2110.4	65.9	9307	6.40	1770.4	65.3	340.1
26	A	9296	3.40	539.5	63.8	9291	3.50	56.8	34.2	482.7
27	U	9279	4.13	2172.5	76.3	9275	4.35	1587.5	40.1	585.0
28	A	9262	3.78	5596.5	106.5	9258	3.50	1013.4	35.0	4583.1
29	C	9242	4.18	1786.1	136.7	9237	4.70	1038.9	45.8	747.1
30	C	9226	4.55	13741.9	95.9	9222	4.50	3422.6	48.4	10319.3
31	U	9210	4.28	6279.1	94.3	9207	4.35	2616.8	41.1	3662.3
32	A	9194	4.83	30477.0	305.1	9190	4.05	2932.7	51.2	27544.3
33	A	9176	4.87	19918.6	309.3	9172	3.50	1047.1	50.8	18871.5
34	U	9160	4.28	9634.1	123.8	9155	3.50	2182.3	56.0	7451.8
35	A	9143	4.60	34636.9	149.9	9139	3.50	3487.4	44.3	31149.5
36	U	9126	3.40	2450.0	190.2	9122	3.50	503.6	35.0	1946.4
37	U	9110	3.95	7661.3	201.5	9106	4.04	3251.7	47.8	4409.6
38	A	9093	4.46	30216.8	187.6	9088	3.96	2508.3	56.6	27708.5
39	U	9077	3.40	62.6	117.7	9074	3.50	327.6	40.4	-265.1
40	A	9061	4.01	8828.7	108.2	9058	4.32	3169.9	28.3	5658.8
41	G	9045	3.40	1087.7	54.4	9044	4.36	997.5	23.3	90.2
42	C	9027	3.40	1330.6	57.8	9025	4.61	659.9	25.1	670.6
43	C	9011	3.59	3034.3	60.4	9008	4.58	1918.2	31.0	1116.2
44	U	8994	3.46	1995.2	66.6	8992	5.16	1638.6	37.6	356.6
45	U	8979	3.46	2118.8	79.0	8975	4.84	1522.9	39.0	595.9
46	U	8963	4.76	7587.0	190.7	8961	4.66	3629.4	37.9	3957.7
47	A	8946	4.51	31240.4	235.0	8943	4.14	2832.7	45.7	28407.8
48	U	8928	3.46	698.6	79.7	8925	3.41	283.1	50.1	415.5
49	C	8913	4.84	16690.4	258.1	8909	4.74	4200.1	49.8	12490.4

50	A	8895	4.71	37354.2	417.7	8892	4.76	4339.0	46.9	33015.1
51	A	8879	4.51	30352.9	222.0	8875	4.25	3267.1	47.2	27085.8
52	C	8860	4.76	8537.6	258.0	8857	3.70	1434.1	30.7	7103.5
53	A	8843	4.71	29619.6	238.3	8840	3.44	1476.2	31.8	28143.4
54	A	8826	4.71	41094.1	246.2	8823	4.41	2029.0	46.3	39065.1
55	U	8810	4.81	8140.6	210.2	8807	4.46	3600.2	36.9	4540.4
56	G	8797	3.89	4128.6	47.7	8795	4.26	2217.5	37.1	1911.1
57	G	8782	3.46	0.0	49.1	8779	4.88	13.5	28.0	-13.5
58	A	8766	3.76	3511.3	70.9	8763	3.65	938.0	21.2	2573.3
59	A	8750	3.46	2528.4	52.0	8747	3.41	542.0	20.7	1986.5
60	U	8734	3.46	0.0	35.9	8736	5.21	139.4	22.8	-139.4
61	C	8717	3.88	6965.2	63.5	8712	4.45	1486.3	30.0	5478.9
62	C	8698	4.80	55.6	142.1	8695	4.97	603.3	46.8	-547.8
63	C	8681	4.59	18612.4	187.2	8678	4.80	3913.8	49.3	14698.7
64	A	8665	4.69	37583.3	263.2	8662	4.59	3940.7	46.3	33642.7
65	A	8648	4.69	31305.7	265.0	8645	4.64	2722.1	50.3	28583.6
66	C	8631	4.43	36580.9	206.4	8628	4.87	4805.8	61.3	31775.1
67	A	8615	4.75	39415.5	311.1	8611	5.05	3462.2	56.9	35953.3
68	A	8598	4.82	31909.1	258.3	8595	4.60	5041.1	52.8	26868.0
69	U	8581	4.70	2355.7	135.2	8580	4.55	867.8	43.4	1487.9
70	U	8567	4.48	9378.1	108.1	8565	4.75	3990.7	32.8	5387.4
71	A	8551	4.48	37097.7	154.4	8548	4.26	2883.7	50.7	34214.0
72	U	8535	4.50	4009.0	126.7	8534	3.16	1042.4	28.5	2966.6
73	C	8518	4.06	6139.3	68.9	8516	3.85	1939.8	26.7	4199.5
74	U	8502	3.24	723.4	68.5	8501	2.36	135.6	25.5	587.8
75	C	8485	3.93	4817.6	82.2	8483	4.38	2266.1	30.5	2551.5
76	A	8469	4.50	17628.5	122.8	8467	4.65	2783.5	27.5	14845.0
77	A	8453	4.65	11399.9	92.2	8450	4.55	1645.2	36.4	9754.7
78	C	8435	4.44	4283.4	73.2	8433	4.49	1942.1	39.6	2341.2
79	A	8419	4.34	7951.3	68.3	8417	4.35	3143.3	29.9	4808.0
80	U	8403	4.39	1697.1	50.1	8402	3.81	967.7	21.2	729.4
81	U	8388	3.24	356.3	46.0	8389	2.36	69.5	20.1	286.8
82	C	8372	4.48	3483.7	131.6	8369	3.11	908.2	24.7	2575.5
83	A	8355	4.39	32095.4	144.4	8354	3.11	1288.6	30.9	30806.8
84	C	8338	4.50	18392.1	170.3	8336	3.44	1082.9	30.6	17309.2
85	C	8320	4.70	14992.9	229.7	8318	4.45	2394.0	35.8	12598.9
86	C	8303	4.33	42265.4	278.9	8301	4.21	4658.1	52.2	37607.3
87	A	8288	4.44	33048.7	308.8	8285	3.68	1822.8	46.4	31225.9
88	A	8271	4.57	26788.6	236.0	8269	2.91	1454.9	25.8	25333.7
89	U	8255	3.24	2970.3	100.6	8253	2.36	271.4	21.1	2698.9
90	U	8239	3.24	2694.2	61.0	8238	2.36	548.3	26.3	2145.9
91	C	8223	3.44	4880.6	64.2	8220	3.11	1909.7	20.4	2970.9
92	U	8206	3.24	492.5	72.1	8203	2.36	285.6	26.0	206.9
93	C	8190	4.62	4639.4	248.6	8186	3.52	1429.9	41.1	3209.4
94	A	8171	4.49	44136.8	293.0	8169	4.36	3582.2	44.2	40554.7
95	U	8155	3.87	6525.9	81.7	8153	4.46	3149.4	28.8	3376.5
96	G	8140	3.53	2129.7	82.4	8138	4.06	1069.3	27.2	1060.4
97	G	8124	3.24	506.4	58.0	8121	3.76	184.5	25.0	321.8

98	U	8106	3.89	3667.5	64.5	8105	3.90	2029.6	29.1	1637.9
99	A	8088	4.34	7497.7	117.5	8085	3.91	2440.2	29.7	5057.5
100	G	8073	4.57	3.9	65.8	8072	2.36	0.0	25.2	3.9
101	C	8060	3.99	3696.1	49.3	8058	4.21	1791.3	26.8	1904.8
102	G	8046	3.24	1476.4	64.4	8043	3.11	300.1	30.2	1176.3
103	C	8029	4.00	6990.5	85.2	8027	3.97	1397.8	30.7	5592.7
104	C	8012	3.99	11155.3	105.5	8010	3.86	2617.9	42.7	8537.4
105	U	7996	3.24	1888.2	57.0	7995	2.94	1036.6	28.1	851.6
106	G	7982	3.24	415.6	69.0	7977	2.36	379.3	25.6	36.4
107	U	7965	3.59	8000.3	63.1	7963	3.93	2887.2	27.3	5113.0
108	G	7951	3.54	3281.9	65.7	7948	3.96	1787.0	31.6	1495.0
109	C	7935	3.38	3270.3	61.7	7933	3.40	1582.9	24.1	1687.4
110	U	7918	3.51	3998.8	61.4	7917	3.64	2330.1	34.0	1668.7
111	U	7902	3.79	6010.4	77.5	7900	3.62	1908.5	42.2	4101.9
112	C	7885	4.00	12245.7	130.7	7883	3.95	4358.6	66.5	7887.1
113	G	7870	4.37	2662.9	91.0	7868	4.38	1196.5	42.5	1466.4
114	G	7854	3.44	1147.5	66.0	7851	2.38	431.6	29.7	715.9
115	U	7837	4.04	6206.5	63.7	7835	3.95	2891.7	29.4	3314.8
116	U	7822	4.04	8660.3	55.9	7820	3.93	4349.1	33.4	4311.3
117	A	7807	3.92	4920.7	48.4	7805	2.38	0.0	26.9	4920.7
118	C	7792	4.02	4063.7	44.3	7790	3.99	1826.8	27.3	2236.9
119	U	7777	4.09	4319.4	40.3	7775	3.78	2090.0	42.7	2229.5
120	U	7761	3.82	1556.0	49.0	7759	2.38	715.2	27.4	840.8
121	C	7746	3.55	801.8	39.1	7743	2.38	178.4	15.6	623.4
122	U	7728	3.59	3442.0	57.8	7727	3.03	998.1	30.1	2443.9
123	A	7712	4.42	31979.5	196.4	7710	4.23	3957.9	48.0	28021.6
124	A	7695	4.37	34549.0	187.2	7693	4.09	3596.2	45.1	30952.8
125	G	7681	4.27	2336.2	70.4	7678	3.95	107.6	40.3	2228.6
126	G	7667	4.23	6063.1	71.7	7664	3.78	2665.7	55.2	3397.4
127	A	7652	4.37	4917.7	77.5	7649	3.33	1273.6	26.0	3644.1
128	A	7634	4.56	2721.8	59.0	7632	3.78	643.6	25.5	2078.2
129	G	7620	4.10	4899.3	63.1	7619	3.61	1764.3	29.4	3135.1
130	U	7606	3.67	1375.7	45.5	7602	2.38	392.2	24.2	983.4
131	C	7590	3.44	1023.6	48.9	7588	2.48	366.7	22.5	657.0
132	C	7573	3.89	4224.6	62.2	7572	3.48	1918.8	24.6	2305.8
133	A	7558	4.04	24558.9	120.7	7554	2.38	432.8	33.5	24126.0
134	C	7541	3.98	10112.3	104.1	7539	3.33	2440.2	32.2	7672.1
135	A	7525	3.44	4753.9	69.9	7522	2.83	796.4	29.8	3957.5
136	C	7508	3.21	4565.4	42.0	7506	3.57	2388.1	28.0	2177.3
137	A	7493	3.51	10287.9	118.2	7490	3.57	1190.5	34.4	9097.4
138	A	7477	4.21	27877.3	259.6	7475	3.78	3499.4	56.0	24378.0
139	A	7460	3.99	36664.9	364.7	7459	3.92	4079.7	49.6	32585.1
140	U	7445	3.21	619.3	74.7	7442	2.17	355.4	39.9	263.9
141	C	7429	3.85	16562.3	150.0	7426	3.97	3607.5	40.9	12954.8
142	A	7413	4.02	38379.3	234.2	7411	3.97	6294.9	38.6	32084.4
143	A	7397	4.16	18228.2	149.4	7394	2.91	1297.1	46.4	16931.1
144	G	7382	3.21	1208.0	85.3	7381	2.17	98.2	13.4	1109.8
145	A	7365	3.21	4310.9	69.0	7361	2.17	302.8	18.2	4008.1

146	U	7350	3.57	4282.0	89.8	7348	3.43	1815.9	35.1	2466.1
147	C	7334	3.91	6571.0	40.7	7331	3.74	2013.8	30.6	4557.2
148	C	7318	3.66	4357.0	54.5	7316	3.74	2562.0	26.8	1795.0
149	G	7304	3.83	2176.4	56.8	7301	3.93	1198.0	34.6	978.5
150	U	7288	3.91	6970.4	42.5	7286	3.77	3223.8	45.6	3746.5
151	U	7273	3.77	2838.8	63.4	7271	3.06	1441.9	31.4	1396.9
152	A	7257	4.02	18017.5	70.6	7254	3.72	1230.6	33.7	16786.9
153	G	7242	3.88	8997.7	84.1	7241	3.78	3825.1	40.6	5172.6
154	A	7228	4.01	28935.1	142.2	7225	3.74	1444.9	39.8	27490.1
155	C	7211	4.02	8789.8	112.4	7209	3.78	3751.3	42.0	5038.5
156	G	7196	3.78	3229.2	86.8	7195	3.97	1768.6	37.9	1460.6
157	U	7182	3.21	2483.8	44.1	7180	3.57	1516.2	28.2	967.6
158	U	7168	3.41	3255.3	36.7	7165	3.36	2332.4	25.9	922.9
159	U	7154	2.79	90.2	29.8	7152	2.46	100.7	31.4	-10.6
160	C	7136	4.09	9366.7	85.9	7134	4.12	4709.4	43.4	4657.3
161	A	7120	3.98	20052.4	125.5	7118	3.95	2972.7	47.9	17079.6
162	G	7105	2.79	886.8	44.6	7103	1.89	98.8	17.7	787.9
163	C	7091	2.79	830.7	36.7	7088	1.91	209.7	16.1	621.1
164	U	7077	3.13	1629.2	39.4	7075	2.25	664.8	19.5	964.4
165	U	7062	2.83	1125.8	46.1	7059	1.65	267.9	16.7	857.9
166	C	7044	2.79	2399.2	60.2	7043	1.49	781.1	14.3	1618.1
167	C	7029	2.79	3223.2	74.2	7027	1.46	490.4	12.6	2732.8
168	A	7014	3.16	5973.6	92.7	7012	1.25	0.0	7.6	5973.6
169	A	6999	3.79	15693.8	132.7	6997	1.25	432.7	11.2	15261.1
170	A	6983	3.94	24073.8	75.7	6980	1.25	160.5	28.5	23913.3
171	A	6967	3.89	36733.5	188.0	6965	2.40	1437.9	45.7	35295.5
172	C	6951	3.89	25660.6	237.6	6948	2.85	2579.1	42.9	23081.4
173	A	6935	3.88	9789.1	150.7	6932	2.86	1940.8	40.0	7848.4
174	G	6921	3.62	4920.5	79.3	6919	3.05	2638.1	50.6	2282.4
175	A	6905	3.74	7844.8	70.6	6903	2.87	1972.6	46.9	5872.1
176	A	6889	3.64	9054.5	69.5	6887	2.86	1822.3	35.5	7232.2
177	G	6874	2.79	575.4	65.2	6872	1.60	261.2	31.8	314.2
178	A	6860	3.41	7813.8	73.5	6858	3.49	2644.9	45.9	5169.0
179	A	6844	3.45	6133.0	62.1	6842	3.54	1989.6	37.5	4143.4
180	U	6829	3.31	3104.3	47.4	6827	3.53	1965.4	23.1	1138.9
181	G	6814	2.83	0.0	39.8	6812	3.96	339.5	42.6	-339.5
182	U	6799	3.59	4104.9	49.0	6797	3.70	2968.1	50.8	1136.8
183	G	6786	3.47	3422.1	40.3	6784	3.57	2681.2	32.4	740.8
184	A	6770	3.51	4057.2	42.7	6768	3.90	1744.8	27.5	2312.5
185	G	6756	3.88	4758.4	79.2	6754	3.80	2576.4	29.0	2182.1
186	A	6740	3.78	9608.3	105.1	6738	3.26	1617.2	29.7	7991.1
187	A	6725	3.83	27358.3	122.0	6723	3.71	2598.3	39.7	24760.1
188	G	6711	3.93	3763.7	62.0	6710	3.62	1961.6	34.4	1802.1
189	G	6698	2.96	1427.9	43.1	6697	3.26	420.1	18.7	1007.8
190	C	6684	3.43	786.7	42.0	6682	3.26	52.1	23.5	734.6
191	U	6669	3.32	2701.0	37.7	6668	4.06	2173.8	26.3	527.2
192	U	6655	2.83	0.0	29.8	6652	4.36	319.2	18.9	-319.2
193	C	6640	2.94	1727.0	59.8	6637	4.16	1791.6	30.9	-64.6

194	C	6623	3.78	11739.0	101.9	6622	4.06	3800.9	33.2	7938.0
195	A	6608	3.64	12729.1	84.0	6606	4.02	2759.8	27.7	9969.3
196	C	6592	3.25	3349.8	58.6	6591	4.06	2360.4	26.6	989.5
197	U	6578	3.54	4077.7	66.7	6576	3.89	2925.8	27.1	1151.9
198	A	6563	3.78	5238.3	47.9	6561	4.06	953.4	29.1	4285.0
199	A	6547	3.64	9871.5	60.3	6545	3.97	4448.5	33.3	5423.0
200	G	6534	3.53	3298.4	46.5	6532	3.92	2606.2	33.6	692.1
201	G	6521	3.78	3483.0	45.1	6518	3.90	2124.3	29.0	1358.7
202	C	6507	3.83	107.7	35.8	6504	3.91	85.0	29.9	22.7
203	U	6492	3.58	6083.3	52.8	6490	3.46	3483.3	56.1	2600.0
204	A	6479	3.64	9877.8	92.0	6477	3.26	1465.7	44.6	8412.1
205	A	6464	3.74	23769.1	145.0	6461	3.46	1430.9	31.8	22338.2
206	C	6448	3.26	4380.0	96.9	6446	3.19	1417.9	26.0	2962.2
207	U	6433	3.01	448.7	53.3	6432	3.10	313.6	25.2	135.0
208	C	6419	3.36	6206.8	72.8	6417	3.44	2699.2	30.5	3507.6
209	U	6404	3.01	440.0	52.8	6403	3.10	399.1	35.4	41.0
210	C	6389	3.48	4757.6	55.5	6387	3.35	2717.5	47.7	2040.2
211	A	6375	3.48	17779.5	63.9	6373	3.10	1888.9	38.1	15890.5
212	A	6360	3.35	10782.3	90.7	6357	3.34	1199.9	33.0	9582.4
213	C	6344	3.01	4110.3	84.4	6342	3.57	2700.2	31.0	1410.1
214	A	6330	3.36	11082.2	115.4	6327	3.50	2383.7	34.0	8698.5
215	G	6316	3.41	5966.5	92.5	6315	3.48	3159.1	33.2	2807.4
216	A	6302	3.01	8178.0	88.4	6298	3.10	607.8	29.5	7570.2
217	C	6287	3.21	10256.1	188.0	6285	4.15	3008.4	52.3	7247.7
218	A	6272	3.76	33717.8	94.8	6270	3.86	5861.0	73.5	27856.7
219	A	6257	3.71	10726.3	183.0	6255	3.64	510.5	65.4	10215.9
220	C	6242	3.44	30222.9	170.9	6240	3.91	4908.2	50.5	25314.7
221	A	6228	3.26	15893.4	112.1	6226	3.85	3615.4	35.9	12278.0
222	A	6214	3.01	2478.9	141.2	6210	3.85	271.0	31.4	2207.9
223	C	6199	3.15	13025.5	182.3	6197	3.57	2546.9	32.2	10478.6
224	A	6183	3.36	15516.0	174.4	6181	3.10	1426.5	37.9	14089.5
225	C	6167	3.01	2298.9	88.6	6165	3.10	434.3	34.4	1864.6
226	C	6152	3.33	9245.6	145.8	6149	3.86	2638.0	36.5	6607.6
227	U	6139	3.71	6001.9	67.9	6136	3.59	2928.8	31.5	3073.1
228	G	6126	3.61	84.7	58.9	6124	2.04	0.0	25.8	84.7
229	C	6113	3.21	7361.1	74.4	6111	2.68	1302.3	29.2	6058.8
230	U	6100	3.21	7377.0	71.9	6098	3.05	2990.0	40.9	4387.0
231	U	6085	2.81	1802.2	75.4	6085	2.49	734.9	41.2	1067.3
232	C	6071	3.27	9213.9	91.6	6069	2.71	2472.4	42.8	6741.5
233	A	6056	3.32	15129.2	88.3	6054	2.52	1341.1	32.5	13788.1
234	U	6041	2.96	2346.8	63.9	6038	2.18	572.2	26.6	1774.5
235	C	6025	3.36	11153.3	71.5	6023	2.65	1660.7	30.7	9492.6
236	A	6009	3.36	11943.0	66.5	6007	2.90	2502.0	35.3	9441.0
237	G	5996	3.46	9329.2	58.2	5993	3.04	2481.1	26.9	6848.1
238	C	5982	3.31	4683.8	67.4	5980	2.55	1004.0	24.6	3679.8
239	U	5969	2.98	658.6	49.3	5965	2.04	109.6	17.2	549.0
240	G	5958	2.81	511.6	42.9	5956	2.04	117.9	12.4	393.6
241	U	5945	2.51	1448.9	31.6	5943	2.04	440.2	13.6	1008.7

242	U	5931	2.51	293.1	37.8	5929	2.17	284.0	20.0	9.1
243	C	5915	3.06	5927.0	95.8	5913	2.94	1595.7	23.6	4331.3
244	C	5900	3.32	14987.7	133.8	5898	3.19	2564.7	43.9	12423.0
245	A	5886	3.46	21704.3	167.6	5883	3.44	4293.9	35.8	17410.4
246	G	5874	3.51	9296.5	97.9	5872	3.39	4659.2	27.7	4637.3
247	A	5861	3.51	4436.9	67.4	5858	2.54	849.8	40.8	3587.1
248	G	5850	3.51	3700.8	70.5	5848	3.28	795.5	39.1	2905.3
249	A	5837	3.51	31475.4	138.5	5834	3.23	1749.9	42.5	29725.5
250	A	5822	2.51	2008.0	129.5	5820	2.49	0.0	17.5	2008.0
251	C	5808	2.91	4298.8	69.1	5805	2.54	323.1	12.2	3975.7
252	C	5793	3.17	6438.1	89.1	5791	2.28	322.0	25.5	6116.1
253	C	5778	3.46	18389.0	120.5	5775	2.89	1631.1	29.5	16758.0
254	C	5764	3.53	1428.8	64.4	5761	2.47	618.8	40.8	810.0
255	C	5748	2.91	4426.3	57.7	5745	3.09	2377.9	59.2	2048.5
256	A	5734	2.91	5731.2	67.3	5732	3.14	2576.8	51.8	3154.4
257	U	5721	2.91	655.4	56.0	5719	3.54	344.1	23.3	311.2
258	C	5706	3.11	4343.0	39.2	5704	3.38	2869.6	29.3	1473.3
259	A	5692	3.21	2186.8	44.9	5691	3.54	629.4	47.4	1557.5
260	U	5679	3.46	3923.0	53.5	5678	3.74	2696.3	34.3	1226.8
261	G	5666	3.49	245.8	49.7	5665	3.75	665.7	23.2	-420.0
262	C	5653	3.23	5194.9	54.7	5651	3.43	2728.6	35.4	2466.3
263	C	5640	3.29	6592.9	52.2	5638	3.40	2341.8	24.9	4251.1
264	U	5626	3.56	80.6	28.5	5624	2.20	0.0	20.9	80.6
265	C	5612	3.37	6114.3	56.7	5610	3.39	3059.8	49.8	3054.5
266	U	5599	2.91	941.4	42.7	5599	2.20	259.6	26.7	681.8
267	C	5584	3.16	2169.9	41.7	5583	2.60	908.3	28.6	1261.6
268	C	5570	3.18	5584.9	40.5	5568	2.80	2796.8	28.7	2788.1
269	U	5556	2.91	1256.4	47.3	5552	2.20	75.6	21.0	1180.8
270	C	5543	2.91	1297.5	92.1	5542	2.20	106.9	25.3	1190.6
271	A	5530	3.46	25862.6	199.7	5527	2.95	2131.4	45.4	23731.2
272	A	5516	3.54	30114.8	243.1	5513	2.71	1490.0	44.1	28624.8
273	C	5502	3.31	3904.1	109.7	5499	2.20	294.0	20.5	3610.1
274	C	5487	3.17	3955.2	37.0	5484	3.04	1527.7	31.0	2427.5
275	U	5474	3.16	5745.6	38.8	5472	3.20	3531.7	37.2	2213.9
276	G	5461	2.60	1442.9	48.5	5460	2.20	578.1	31.7	864.8
277	C	5448	2.54	1606.7	28.4	5445	2.20	766.6	18.5	840.1
278	U	5434	3.73	1335.0	79.9	5431	2.20	317.9	30.8	1017.1
279	U	5419	3.12	12591.2	326.2	5418	4.50	1812.5	80.2	10778.7
280	C	5407	3.54	65704.1	620.0	5404	3.95	4217.2	139.2	61486.9
281	A	5393	3.74	12353.9	178.8	5390	2.20	695.2	35.1	11658.7
282	G	5382	2.54	2377.8	55.6	5381	2.20	452.7	33.6	1925.1
283	U	5371	3.35	4159.9	53.4	5368	2.44	1524.9	28.2	2635.0
284	A	5357	3.02	2187.2	52.9	5355	2.20	1.4	14.3	2185.8
285	C	5343	3.78	1543.8	76.4	5340	3.20	55.9	14.5	1487.8
286	C	5329	3.61	22242.3	200.8	5327	3.08	3257.7	40.3	18984.6
287	A	5316	3.34	26341.5	221.9	5313	2.20	534.8	27.1	25806.8
288	C	5302	2.54	515.4	123.4	5298	3.23	182.0	18.6	333.4
289	C	5287	3.08	4501.5	43.1	5285	3.27	1678.1	28.8	2823.4

290	U	5274	2.56	782.6	41.2	5271	2.37	666.6	39.0	116.1
291	C	5261	2.88	3606.8	60.8	5259	3.28	1644.3	35.6	1962.5
292	C	5247	3.32	11212.8	60.7	5245	3.27	3830.4	33.5	7382.5
293	A	5233	3.41	20234.2	80.1	5230	3.33	1982.0	48.0	18252.2
294	C	5218	3.41	19962.1	153.1	5215	3.88	4171.3	75.3	15790.9
295	A	5204	3.46	37193.2	176.1	5201	3.51	6779.5	124.5	30413.7
296	G	5191	3.46	4625.0	67.7	5189	2.74	2138.0	67.2	2486.9
297	A	5176	3.46	30079.6	66.9	5173	3.33	3112.8	70.8	26966.8
298	A	5161	3.34	27842.5	96.3	5159	3.13	3364.1	63.6	24478.4
299	U	5149	2.50	2167.9	88.1	5146	2.30	1093.3	44.5	1074.6
300	G	5137	2.11	52.3	22.7	5136	1.57	306.8	26.6	-254.5
301	G	5127	3.49	1485.9	51.2	5124	3.04	816.2	33.4	669.7
302	G	5115	3.04	7220.6	169.0	5114	3.14	2171.7	50.9	5048.9
303	C	5103	3.46	24513.8	283.0	5100	3.33	3823.3	47.0	20690.4
304	C	5091	3.72	644.0	50.5	5088	3.57	28.1	11.5	615.9
305	G	5078	2.66	382.0	17.5	5076	1.53	75.1	11.8	306.9
306	U	5066	2.46	717.0	37.7	5064	1.91	337.6	20.5	379.4
307	A	5051	2.92	4782.9	51.6	5049	2.73	1469.5	36.5	3313.3
308	C	5036	2.83	2003.4	51.7	5032	2.40	1124.1	33.3	879.3
309	C	5023	2.22	899.5	85.8	5020	2.47	342.9	39.9	556.6
310	C	5011	3.49	11862.6	128.5	5009	3.33	3058.2	63.7	8804.4
311	A	4996	3.52	35641.7	174.8	4994	3.62	2082.8	54.2	33558.8
312	C	4982	3.47	30937.2	243.0	4980	3.53	4722.0	50.3	26215.3
313	A	4969	2.94	5853.1	161.7	4967	2.58	1191.6	33.6	4661.4
314	G	4958	2.31	490.5	35.0	4955	1.53	111.4	30.2	379.1
315	C	4946	2.97	3850.3	65.0	4943	2.76	2357.3	41.7	1493.0
316	A	4932	3.02	5805.5	58.2	4929	3.14	2490.1	48.9	3315.3
317	G	4921	3.02	3984.2	85.6	4917	3.29	2658.3	30.9	1325.9
318	U	4909	3.16	8041.5	94.8	4906	3.28	5415.3	28.0	2626.2
319	G	4897	2.77	502.0	47.2	4893	2.45	489.6	29.8	12.5
320	C	4882	3.07	6250.2	80.2	4880	3.23	4184.0	33.4	2066.2
321	A	4869	3.00	5214.8	82.6	4866	3.18	2694.7	43.2	2520.1
322	U	4857	3.10	3947.1	71.4	4854	3.23	2803.1	51.3	1144.1
323	G	4846	3.15	5430.8	56.9	4844	3.18	3880.4	36.9	1550.3
324	A	4834	2.77	1705.7	35.4	4831	2.36	815.9	26.9	889.8
325	U	4823	3.68	0.2	63.6	4820	2.29	0.0	28.2	0.2
326	G	4811	3.34	2352.5	84.4	4809	2.78	1003.1	47.6	1349.4
327	A	4800	3.36	32607.7	74.1	4798	3.02	2452.5	72.8	30155.2
328	C	4786	3.42	28850.6	98.6	4784	3.61	3202.8	68.6	25647.8
329	C	4772	3.34	19504.2	157.7	4769	3.58	6166.9	61.7	13337.3
330	C	4759	3.29	20163.4	169.5	4756	3.33	4771.6	39.3	15391.8
331	A	4747	3.22	14578.0	229.9	4743	2.88	975.6	41.7	13602.4
332	A	4735	3.34	30050.5	259.3	4731	3.42	3083.3	41.9	26967.3
333	A	4722	3.42	37674.6	135.2	4719	3.23	4534.9	67.7	33139.8
334	A	4709	3.24	20971.2	154.1	4706	2.29	999.5	50.8	19971.6
335	C	4695	2.77	2851.5	139.0	4691	2.90	810.0	47.1	2041.5
336	C	4683	3.32	15273.7	172.2	4680	2.89	4371.7	52.1	10902.0
337	A	4670	3.32	26119.5	230.7	4668	3.04	2812.2	58.6	23307.2

338	A	4657	3.13	24439.9	295.9	4654	2.70	2648.0	81.5	21791.9
339	G	4645	2.77	1310.2	100.0	4641	2.29	238.8	57.9	1071.4
340	C	4633	2.77	6070.3	83.3	4630	2.69	2383.9	54.2	3686.3
341	C	4620	3.20	1801.4	131.5	4617	2.29	829.6	40.3	971.8
342	A	4608	2.97	15992.8	202.2	4605	2.29	1326.5	33.9	14666.2
343	A	4595	2.92	11111.7	123.9	4592	2.45	1432.9	47.5	9678.9
344	U	4583	2.70	4075.1	98.2	4580	2.69	2464.0	41.4	1611.0
345	C	4570	1.88	153.6	96.6	4568	2.29	307.9	48.0	-154.3
346	C	4558	3.15	22546.5	261.5	4555	3.14	4709.6	55.0	17836.9
347	A	4545	3.27	18829.6	160.0	4542	2.55	2105.6	48.2	16723.9
348	U	4532	1.94	1526.9	145.6	4528	3.64	632.9	65.6	894.0
349	C	4518	3.22	31523.7	140.0	4515	3.49	5510.7	51.4	26013.1
350	U	4505	3.11	9188.9	136.0	4502	3.14	5887.0	64.8	3301.9
351	G	4494	1.88	1474.4	48.1	4492	2.81	1544.9	44.6	-70.5
352	G	4483	2.03	901.0	35.7	4480	2.89	1172.3	43.7	-271.3
353	U	4472	2.58	3507.9	62.2	4469	3.10	3294.8	56.7	213.1
354	U	4459	2.73	3863.1	66.2	4456	3.04	2994.1	59.3	869.1
355	G	4449	2.62	3042.4	64.3	4447	3.19	2980.1	80.0	62.3
356	G	4439	3.27	8480.8	107.3	4436	3.44	3862.8	57.9	4618.0
357	U	4427	3.47	11061.9	134.9	4425	3.25	2887.1	66.5	8174.8
358	C	4414	3.11	29163.4	383.0	4412	3.44	7461.7	92.2	21701.7
359	A	4403	3.07	26005.4	298.3	4400	3.49	8404.8	87.3	17600.5
360	U	4392	3.11	9722.9	116.9	4388	3.17	6219.3	105.5	3503.7
361	U	4379	1.88	120.2	53.1	4376	2.29	707.2	38.0	-587.0
362	U	4368	1.88	545.7	27.8	4365	2.29	1066.0	36.6	-520.3
363	U	4356	1.92	1265.9	66.7	4353	3.00	1656.6	42.7	-390.7
364	A	4344	2.69	8134.9	115.6	4340	2.99	2912.7	57.0	5222.3
365	C	4332	2.90	2285.2	197.0	4327	3.01	2719.0	57.2	-433.7
366	G	4323	3.63	3335.5	84.0	4319	3.84	2879.4	68.3	456.1
367	G	4313	3.04	2904.3	68.4	4308	3.74	1369.4	93.3	1534.9
368	A	4301	3.13	1524.4	87.8	4297	2.34	0.0	26.1	1524.4
369	C	4289	2.99	11785.4	159.7	4286	2.58	2732.7	46.1	9052.7
370	A	4277	3.33	15405.9	236.6	4273	2.34	385.5	47.6	15020.4
371	C	4262	3.33	26580.8	238.7	4258	2.96	3202.3	31.7	23378.5
372	C	4250	1.33	189.4	145.4	4245	2.48	30.7	47.9	158.6
373	C	4237	3.48	13795.9	158.9	4233	3.34	5337.1	62.3	8458.8
374	A	4225	3.42	37690.3	281.7	4222	3.10	5266.1	78.8	32424.3
375	U	4213	3.04	10028.6	226.2	4211	3.09	2835.0	61.3	7193.6
376	C	4201	3.13	14835.5	124.7	4197	3.44	4713.5	54.4	10122.0
377	U	4189	3.37	13935.8	156.0	4185	3.20	7074.3	89.2	6861.5
378	A	4177	3.09	36425.5	427.3	4174	3.15	6570.5	91.5	29855.1
379	U	4167	3.13	10028.5	315.1	4163	3.54	7360.3	64.8	2668.2
380	G	4156	3.53	5523.7	198.0	4153	3.14	3252.9	138.1	2270.8
381	A	4145	2.46	4247.1	83.9	4143	2.34	1712.3	49.2	2534.8
382	U	4132	1.45	567.6	37.3	4130	2.34	1176.1	36.4	-608.6
383	U	4121	1.33	549.4	24.2	4118	2.77	1076.1	34.3	-526.7
384	C	4110	2.49	2090.2	149.0	4106	2.65	2414.0	43.6	-323.8
385	C	4100	4.77	924.9	243.9	4100	2.34	1155.4	55.0	-230.5

386	G	4090	4.56	10796.8	198.0	4090	4.50	9664.9	159.6	1131.9
387	U	4078	2.55	2544.2	123.8	4074	4.50	1470.5	201.5	1073.7
388	A	4067	2.98	10483.8	137.6	4064	2.95	5243.4	77.5	5240.4
389	U	4058	3.18	6075.1	150.4	4055	2.70	3258.6	62.8	2816.5
390	A	4047	3.46	11989.3	106.5	4042	3.09	73.9	74.7	11915.4
391	C	4034	3.22	34167.7	287.9	4030	3.05	4377.4	98.9	29790.3
392	A	4023	3.46	43794.8	559.9	4021	2.76	3171.9	60.4	40622.8
393	C	4010	3.69	9487.5	234.3	4006	2.56	1714.4	52.5	7773.0
394	C	3997	3.10	11202.7	94.9	3994	2.76	3245.8	51.3	7956.9
395	U	3987	2.79	3945.7	114.2	3983	2.95	2048.7	66.3	1897.0
396	U	3977	3.18	13978.5	189.1	3973	2.95	6779.5	86.7	7199.0
397	A	3965	3.32	38246.0	196.8	3962	2.95	7608.6	79.2	30637.4
398	U	3954	3.46	9061.0	138.8	3950	2.95	3921.9	80.9	5139.1
399	C	3941	3.22	25338.1	212.6	3938	2.85	6754.1	96.1	18584.0
400	A	3930	3.46	26341.8	331.6	3924	2.46	2942.1	98.8	23399.7
401	A	3918	3.41	50283.2	376.3	3915	2.71	7897.0	98.1	42386.1
402	A	3907	3.06	23421.4	260.6	3904	1.90	0.0	55.1	23421.4
403	U	3897	3.16	4834.2	191.8	3892	1.90	805.2	31.0	4029.0
404	G	3886	3.63	1962.2	126.6	3881	1.90	0.0	65.2	1962.2
405	U	3876	2.46	1254.4	281.4	3870	1.90	4213.3	123.2	-2958.9
406	C	3865	3.55	69795.2	511.3	3861	3.47	4969.0	130.1	64826.1
407	G	3854	3.69	3415.3	142.0	3850	3.07	1039.2	105.6	2376.0
408	C	3842	2.46	1591.3	56.0	3839	2.31	1220.1	52.2	371.2
409	C	3829	2.94	2423.6	56.7	3825	3.13	2140.3	40.5	283.3
410	U	3818	2.47	3385.0	74.0	3814	3.10	3242.9	56.0	142.1
411	A	3808	3.35	12151.6	167.2	3804	3.00	6989.1	67.0	5162.5
412	U	3795	3.78	2693.0	145.6	3795	2.97	1501.2	71.3	1191.8
413	G	3782	3.97	6230.2	165.0	3786	4.50	2592.8	318.5	3637.4
414	U	3769	3.78	27293.9	289.8	3765	4.50	3563.9	298.8	23730.0
415	A	3758	2.45	3781.6	109.3	3753	2.27	1802.0	68.3	1979.6
416	C	3748	3.40	3681.9	76.5	3743	3.07	1977.7	72.0	1704.2
417	U	3737	3.44	12641.1	65.4	3733	3.30	4614.0	48.7	8027.1
418	U	3726	2.90	3550.3	132.6	3723	2.94	3133.3	65.7	417.0
419	U	3716	2.21	3.9	92.9	3712	1.99	465.9	72.7	-462.0
420	C	3705	3.41	28821.6	75.6	3701	3.12	5810.9	48.1	23010.8
421	C	3693	2.69	3440.7	128.6	3689	1.55	124.0	40.6	3316.7
422	A	3680	2.21	1481.3	27.6	3676	2.05	1025.0	28.9	456.4
423	C	3668	2.45	2156.1	41.4	3664	1.55	597.6	26.1	1558.5
424	C	3657	3.07	1692.4	43.1	3652	1.55	264.4	19.4	1428.0
425	U	3644	3.41	2518.9	23.7	3639	1.55	363.9	18.2	2155.0
426	G	3634	2.21	20.2	28.9	3629	1.55	0.0	18.4	20.2
427	G	3623	3.31	4527.6	43.0	3619	2.22	1889.1	60.0	2638.5
428	G	3611	3.41	14825.3	48.4	3606	2.36	3346.5	59.6	11478.8
429	C	3600	3.41	6468.8	51.0	3594	1.55	566.5	39.9	5902.3
430	C	3589	3.61	1736.0	87.9	3584	3.35	508.8	61.4	1227.3
431	A	3578	3.56	20617.2	45.7	3573	2.83	2892.4	83.3	17724.8
432	C	3567	3.56	15672.5	43.7	3563	1.55	133.3	29.7	15539.2
433	A	3556	3.40	3341.7	75.7	3552	1.98	532.0	31.7	2809.7

434	A	3545	2.43	6348.3	141.2	3540	2.38	2577.1	94.6	3771.1
435	U	3534	3.86	28543.9	286.9	3528	3.10	4732.3	89.6	23811.6
436	C	3520	3.84	11923.8	344.2	3520	3.19	130.8	97.8	11793.0

DMS_example_2_peaks.txt

seqnum	seq	RX.pos	RX.sigma	RX.area	RX.rms	BG.pos	BG.sigma	BG.area	BG.rms	(RX.area-BG.area)
6	G	9494	3.80	988.3	207.0	9492	2.65	36.8	26.3	951.5
7	A	9480	5.00	17371.3	206.1	9475	1.99	718.8	17.1	16652.6
8	A	9464	5.20	22843.4	265.6	9458	1.99	0.0	45.9	22843.4
9	C	9447	5.07	33312.0	307.0	9441	4.89	6037.1	69.7	27274.9
10	U	9432	4.85	12214.6	215.6	9426	4.43	3511.2	39.2	8703.4
11	U	9413	5.55	18.2	254.8	9408	3.45	343.1	48.8	-324.9
12	C	9395	5.05	29128.5	305.8	9391	5.00	6625.5	54.1	22503.0
13	U	9379	4.76	11281.8	183.0	9375	4.87	3986.2	60.9	7295.6
14	A	9362	5.09	19754.8	187.7	9358	3.95	2029.8	34.0	17724.9
15	G	9346	2.66	1378.2	102.7	9342	2.73	353.2	25.7	1025.0
16	U	9330	3.91	2101.6	63.0	9325	3.17	591.1	21.5	1510.5
17	A	9314	4.15	6954.1	55.9	9310	4.00	2595.5	24.2	4358.6
18	U	9298	2.56	191.3	47.4	9292	2.32	524.0	29.5	-332.7
19	A	9281	4.10	7274.3	65.0	9277	4.71	3376.4	49.4	3897.8
20	U	9264	3.25	2300.3	76.5	9260	4.75	1678.8	54.3	621.6
21	U	9248	3.76	4209.4	85.4	9244	2.89	1074.2	39.7	3135.2
22	C	9230	4.65	17683.6	119.0	9225	5.00	4029.7	50.1	13653.9
23	U	9214	4.71	9983.6	121.1	9209	4.99	5214.5	46.9	4769.1
24	G	9200	4.46	5667.4	73.3	9195	4.64	3357.2	22.5	2310.2
25	U	9183	3.06	2607.4	93.1	9180	4.24	1849.1	24.0	758.3
26	A	9167	2.56	844.0	32.4	9163	1.99	95.5	19.8	748.4
27	U	9152	3.03	1984.7	61.3	9148	2.99	1060.9	22.3	923.7
28	A	9135	3.41	5546.5	71.9	9132	2.76	1120.7	19.9	4425.9
29	C	9117	3.26	4487.9	123.3	9114	1.99	116.8	18.4	4371.0
30	C	9099	4.41	16389.0	82.5	9095	1.99	997.7	12.5	15391.3
31	U	9083	4.36	5848.5	70.7	9079	1.99	1.5	16.8	5847.0
32	A	9067	4.41	20567.3	106.4	9062	1.99	726.5	17.8	19840.8
33	A	9050	4.41	15407.6	132.1	9047	2.61	927.4	35.1	14480.3
34	U	9034	4.51	7083.1	206.3	9031	3.12	1667.3	51.1	5415.8
35	A	9016	4.31	26115.5	242.6	9013	4.24	5594.0	29.0	20521.5
36	U	9000	3.46	2993.4	134.4	8997	4.24	2582.7	24.9	410.7
37	U	8984	3.46	5718.0	170.7	8981	3.89	2895.9	25.8	2822.0
38	A	8967	4.31	23507.9	111.4	8965	3.94	2663.2	34.0	20844.7
39	U	8951	4.36	408.4	51.4	8949	1.99	88.8	30.8	319.6
40	A	8935	3.93	7168.8	71.3	8932	3.98	2173.1	33.3	4995.7
41	G	8920	3.46	2706.0	63.5	8918	4.08	1441.4	32.6	1264.6

42	C	8903	3.46	1516.4	70.8	8899	3.04	717.5	27.8	798.9
43	C	8885	3.46	4912.5	59.1	8882	4.24	2965.3	32.0	1947.2
44	U	8870	3.46	3282.4	47.5	8867	4.33	2651.2	28.0	631.2
45	U	8854	3.46	3127.9	47.4	8851	4.44	1902.7	24.4	1225.2
46	U	8838	4.46	6406.8	151.3	8835	4.44	2370.9	31.7	4036.0
47	A	8821	4.36	24383.8	158.6	8818	4.01	3322.2	43.4	21061.6
48	U	8805	3.46	802.9	100.3	8803	1.99	246.9	24.1	556.0
49	C	8788	4.35	17554.2	113.8	8785	3.88	2468.1	40.9	15086.0
50	A	8771	4.50	25584.5	116.3	8767	4.03	2902.4	42.3	22682.1
51	A	8754	4.50	26623.3	158.1	8751	3.79	3098.6	27.2	23524.8
52	C	8736	4.50	16397.9	163.1	8733	3.51	1885.7	26.5	14512.2
53	A	8719	4.50	21234.9	138.7	8716	2.81	994.0	38.6	20240.9
54	A	8702	4.65	31937.3	131.4	8698	4.29	2641.0	38.7	29296.3
55	U	8686	4.65	9589.9	107.4	8683	4.39	4440.7	28.6	5149.3
56	G	8673	3.94	5295.3	40.9	8670	4.25	2577.0	24.2	2718.3
57	G	8658	3.50	372.9	40.0	8655	2.49	126.3	18.5	246.7
58	A	8643	3.50	3093.2	35.6	8639	2.49	430.0	16.4	2663.2
59	A	8627	3.50	3059.0	29.6	8624	2.79	875.7	16.0	2183.3
60	U	8610	3.50	878.5	41.7	8608	2.49	213.9	15.4	664.6
61	C	8594	3.90	7231.2	77.4	8590	3.04	1321.4	19.9	5909.9
62	C	8576	3.50	1810.8	106.5	8575	2.49	186.3	24.3	1624.4
63	C	8559	4.45	22348.5	95.2	8557	3.91	2529.5	32.0	19818.9
64	A	8543	4.70	27499.8	226.7	8541	4.06	2965.8	32.5	24534.0
65	A	8526	4.75	24701.7	223.0	8525	4.39	2489.2	37.5	22212.5
66	C	8509	4.59	34316.2	146.9	8507	4.49	4353.9	33.9	29962.3
67	A	8493	4.70	28398.4	214.3	8491	4.64	3815.2	45.0	24583.2
68	A	8476	4.70	30365.0	211.0	8474	4.54	6338.2	52.5	24026.8
69	U	8461	3.59	4581.7	62.6	8459	4.20	1797.3	31.1	2784.5
70	U	8446	4.70	9473.3	197.6	8443	4.25	2875.0	31.2	6598.3
71	A	8429	4.50	28453.2	239.5	8427	4.06	3714.2	30.9	24739.0
72	U	8414	3.50	4427.4	59.3	8411	3.37	1212.1	29.0	3215.3
73	C	8397	3.98	7099.4	52.3	8394	3.61	2986.7	29.9	4112.7
74	U	8381	3.50	1035.0	49.5	8379	2.49	34.4	21.0	1000.7
75	C	8364	3.50	4048.3	41.1	8362	3.23	934.6	25.0	3113.7
76	A	8349	4.23	13650.3	154.3	8346	4.27	2752.9	30.7	10897.4
77	A	8333	4.53	10560.1	101.6	8330	4.01	2664.8	43.6	7895.3
78	C	8316	4.13	3655.6	51.9	8314	3.96	1082.4	50.8	2573.2
79	A	8299	4.18	9757.8	53.4	8297	4.57	5007.2	28.6	4750.6
80	U	8284	3.88	2637.4	35.0	8282	4.16	1748.1	14.2	889.3
81	U	8268	3.33	154.1	25.1	8268	2.72	87.3	13.7	66.8
82	C	8252	3.33	2659.3	41.0	8249	2.88	527.0	17.2	2132.3
83	A	8236	4.48	22992.5	159.5	8235	3.72	2054.8	40.3	20937.7
84	C	8218	4.53	19438.6	147.0	8216	3.23	1526.0	38.1	17912.6
85	C	8201	4.48	19020.9	168.6	8199	4.17	2538.0	41.7	16482.9
86	C	8184	4.53	34982.2	196.4	8182	4.07	4960.6	49.1	30021.7
87	A	8168	4.48	25345.7	204.6	8167	3.21	1209.6	34.6	24136.0
88	A	8152	4.33	37823.7	195.7	8150	3.72	3400.6	41.9	34423.1
89	U	8138	3.58	3954.8	115.6	8135	2.72	681.7	32.2	3273.0

90	U	8121	3.33	3151.0	73.5	8118	2.72	844.5	27.6	2306.5
91	C	8104	3.33	6415.2	65.7	8101	2.92	2202.6	27.8	4212.7
92	U	8087	3.33	1613.9	72.4	8085	2.72	206.7	20.0	1407.1
93	C	8070	4.01	8456.6	87.0	8068	2.76	1168.1	29.0	7288.5
94	A	8053	4.23	33807.0	99.1	8051	4.14	3596.4	44.6	30210.5
95	U	8037	4.23	8662.1	102.5	8034	4.14	3949.7	53.1	4712.4
96	G	8022	3.54	3179.0	72.9	8020	2.91	1267.3	23.9	1911.7
97	G	8006	3.33	1096.6	50.8	8004	2.72	116.2	14.4	980.4
98	U	7989	3.33	3198.3	62.8	7987	2.79	997.4	29.7	2200.9
99	A	7971	4.01	7260.4	188.9	7967	3.50	2956.8	33.1	4303.6
100	G	7956	4.56	0.0	134.9	7954	2.75	11.1	16.7	-11.1
101	C	7942	3.86	4349.8	36.7	7941	4.11	2026.8	24.4	2323.0
102	G	7929	3.56	3281.6	44.3	7927	3.24	741.9	38.5	2539.6
103	C	7912	4.09	13637.2	84.8	7910	3.67	1476.1	37.8	12161.0
104	C	7895	4.05	15928.2	126.4	7893	3.83	3662.8	40.2	12265.4
105	U	7879	2.86	2573.2	75.2	7877	2.75	1040.6	30.5	1532.6
106	G	7865	2.86	1394.3	75.5	7861	2.75	453.9	36.9	940.3
107	U	7849	3.81	13253.0	111.7	7847	3.87	4639.9	39.8	8613.2
108	G	7834	4.01	4737.5	52.8	7831	3.01	1143.3	51.7	3594.2
109	C	7818	3.86	9657.8	65.0	7817	3.70	2465.2	46.4	7192.6
110	U	7802	3.62	7216.2	68.1	7800	3.87	3700.9	37.7	3515.3
111	U	7787	3.86	8071.5	102.2	7784	3.77	2882.3	65.4	5189.2
112	C	7769	4.05	19293.8	154.6	7766	3.87	6180.6	68.9	13113.2
113	G	7754	4.14	4192.2	129.8	7752	3.01	1579.6	29.2	2612.6
114	G	7738	2.86	1972.2	57.2	7736	2.75	642.0	38.9	1330.2
115	U	7722	3.86	8083.7	58.8	7720	3.73	3214.6	35.2	4869.2
116	U	7707	3.96	10215.5	40.3	7705	3.68	4030.4	36.0	6185.1
117	A	7692	3.71	4041.3	68.3	7690	2.75	0.0	28.8	4041.3
118	C	7678	3.62	4087.9	53.8	7675	3.35	1630.9	18.1	2457.0
119	U	7663	3.66	5185.1	48.2	7661	3.30	2408.7	24.6	2776.4
120	U	7647	2.86	1710.9	43.1	7645	2.75	534.6	24.5	1176.3
121	C	7630	2.86	1778.8	38.6	7628	2.75	960.0	22.6	818.8
122	U	7614	3.06	3447.1	78.5	7612	2.75	1081.8	27.1	2365.4
123	A	7598	4.21	24690.8	133.4	7596	4.09	3700.8	47.3	20990.1
124	A	7581	4.19	26104.0	88.3	7579	4.15	3812.0	34.2	22292.0
125	G	7567	4.16	2289.3	42.0	7564	3.77	642.9	16.2	1646.4
126	G	7553	3.81	5572.4	49.8	7551	3.53	2292.7	23.1	3279.7
127	A	7538	3.59	3959.6	25.7	7536	3.06	1178.6	17.6	2781.0
128	A	7522	3.68	2896.7	39.5	7519	3.66	915.9	37.1	1980.8
129	G	7508	3.96	9667.7	42.2	7506	3.89	3942.5	33.3	5725.2
130	U	7493	3.63	1570.6	49.5	7492	2.36	206.5	19.9	1364.1
131	C	7477	2.96	1337.9	33.8	7474	2.36	547.8	20.1	790.1
132	C	7461	3.53	4066.1	80.6	7458	2.69	1021.4	23.8	3044.7
133	A	7445	3.91	18556.3	51.9	7443	2.77	918.7	25.1	17637.7
134	C	7428	3.56	7731.1	85.1	7426	2.89	1762.7	20.6	5968.4
135	A	7412	2.96	3920.7	64.4	7410	2.53	857.2	21.8	3063.6
136	C	7396	2.96	4337.7	80.4	7394	2.69	1644.9	21.6	2692.8
137	A	7380	3.38	7529.8	168.8	7378	2.51	802.6	34.0	6727.2

138	A	7364	3.96	20518.3	217.6	7362	2.96	2255.5	48.2	18262.8
139	A	7348	3.91	30440.2	244.7	7347	2.94	2888.4	41.3	27551.8
140	U	7333	3.00	3298.2	72.9	7331	2.36	0.0	24.5	3298.2
141	C	7317	4.05	20553.3	89.1	7314	2.94	1962.9	41.5	18590.3
142	A	7301	4.11	28654.2	90.2	7299	3.34	3904.5	40.0	24749.7
143	A	7285	3.96	19121.7	136.5	7283	3.06	2461.9	30.2	16659.8
144	G	7271	2.96	2301.6	45.8	7269	2.36	347.8	21.3	1953.8
145	A	7253	3.27	4112.6	51.1	7251	2.36	331.5	20.0	3781.2
146	U	7238	3.63	5513.1	51.3	7236	3.01	1776.0	25.4	3737.0
147	C	7223	3.77	8495.6	45.1	7221	3.34	2153.8	34.4	6341.9
148	C	7207	3.70	6033.8	60.3	7205	3.50	2656.1	23.1	3377.7
149	G	7193	3.35	3528.3	32.2	7191	3.44	1306.6	21.4	2221.8
150	U	7178	3.78	9379.1	51.0	7176	3.54	3459.2	24.3	5919.9
151	U	7163	3.89	4582.3	35.7	7161	3.16	1614.7	28.3	2967.7
152	A	7147	3.88	17401.5	47.6	7144	3.50	1558.3	27.6	15843.1
153	G	7133	3.89	9012.1	60.9	7131	3.59	2835.6	25.8	6176.5
154	A	7118	3.93	24449.7	38.0	7116	3.70	2014.6	22.2	22435.1
155	C	7101	3.89	10938.7	37.1	7099	3.78	4131.9	28.2	6806.8
156	G	7087	3.51	4538.8	61.7	7085	3.90	2470.6	19.8	2068.2
157	U	7073	3.43	4075.1	35.5	7070	3.59	2483.6	28.3	1591.5
158	U	7058	3.33	4503.1	40.8	7056	3.40	2457.8	17.9	2045.3
159	U	7043	3.08	403.3	41.9	7040	2.36	447.6	24.6	-44.3
160	C	7027	3.58	8356.8	51.9	7025	3.82	3317.9	30.3	5038.9
161	A	7012	3.83	16578.1	91.6	7010	3.90	3615.8	21.7	12962.3
162	G	6998	4.03	587.1	69.2	6995	2.36	30.6	13.4	556.4
163	C	6983	3.08	1892.2	43.0	6980	3.16	1097.3	21.7	794.8
164	U	6968	3.23	3025.8	32.9	6967	3.15	1674.3	31.5	1351.5
165	U	6954	3.08	2295.9	37.6	6952	2.52	917.1	31.1	1378.9
166	C	6937	3.32	4423.6	49.9	6935	2.97	2035.3	24.8	2388.3
167	C	6921	3.19	5184.3	67.1	6919	2.83	1901.6	34.6	3282.7
168	A	6907	3.61	6909.4	139.5	6905	2.36	733.9	18.4	6175.4
169	A	6891	3.70	13500.1	100.1	6889	2.21	995.4	15.6	12504.7
170	A	6876	3.74	17721.5	96.4	6874	1.53	148.8	18.8	17572.7
171	A	6860	3.83	26261.3	152.0	6858	2.21	1226.4	20.7	25034.8
172	C	6844	3.83	29057.8	214.1	6842	2.10	991.0	20.0	28066.8
173	A	6828	3.73	9007.0	152.0	6825	2.05	990.8	26.1	8016.2
174	G	6814	3.04	4559.1	69.2	6812	1.53	308.1	15.2	4251.0
175	A	6798	3.23	5839.7	73.6	6796	1.53	332.7	29.3	5507.0
176	A	6783	3.29	9585.9	55.0	6781	2.72	2418.4	33.0	7167.5
177	G	6769	3.04	1417.6	50.4	6767	1.53	60.2	15.1	1357.3
178	A	6754	3.35	5559.2	53.1	6751	2.79	1342.2	22.8	4217.0
179	A	6738	3.40	6520.3	44.8	6736	3.14	2410.1	26.0	4110.2
180	U	6723	3.16	3834.7	35.4	6721	3.14	1880.7	21.1	1953.9
181	G	6710	3.04	272.1	30.9	6706	2.39	221.1	29.6	51.0
182	U	6694	3.29	6219.9	33.6	6692	3.83	3603.3	23.0	2616.6
183	G	6681	3.35	4163.6	29.6	6678	3.73	2080.2	26.5	2083.4
184	A	6665	3.50	5170.2	36.3	6663	3.56	2441.9	24.2	2728.4
185	G	6651	3.38	4955.2	29.6	6650	3.77	2306.4	28.0	2648.7

186	A	6636	3.90	8675.8	110.4	6633	4.03	1221.2	24.5	7454.7
187	A	6620	3.80	23064.1	178.2	6618	3.83	3632.2	39.5	19431.9
188	G	6607	3.27	4484.0	57.6	6606	3.66	2173.8	25.9	2310.2
189	G	6594	3.55	1282.2	40.6	6593	3.88	418.6	18.3	863.6
190	C	6579	3.55	3436.4	43.3	6577	3.56	1035.1	15.7	2401.3
191	U	6565	3.04	4236.2	53.5	6564	3.73	2505.9	17.9	1730.3
192	U	6550	2.75	0.0	57.5	6549	2.56	41.3	14.2	-41.3
193	C	6539	3.56	2438.5	170.2	6534	3.51	1638.3	17.1	800.2
194	C	6521	3.85	11291.7	122.5	6519	3.83	3321.7	26.0	7970.0
195	A	6505	3.75	14900.2	86.4	6503	3.72	3011.5	31.3	11888.7
196	C	6490	3.60	8045.5	43.6	6488	3.68	2755.9	28.8	5289.6
197	U	6475	3.60	5458.7	34.8	6473	3.72	2708.5	27.1	2750.2
198	A	6460	3.42	4996.6	42.2	6457	3.38	1136.3	21.6	3860.3
199	A	6445	3.70	10234.0	95.7	6443	3.75	3945.3	39.4	6288.7
200	G	6431	3.75	4632.9	62.2	6430	3.96	2410.2	27.5	2222.7
201	G	6418	3.27	3788.8	37.8	6416	3.47	1634.8	33.4	2153.9
202	C	6404	3.56	227.7	47.5	6402	3.71	252.7	35.4	-25.0
203	U	6390	3.45	6209.8	49.6	6388	3.42	2930.0	45.2	3279.8
204	A	6377	3.60	7819.8	73.1	6374	3.15	1078.7	31.7	6741.1
205	A	6362	3.60	20405.5	60.3	6360	3.18	2095.5	25.3	18310.0
206	C	6347	3.51	8733.0	64.5	6345	3.01	1625.4	21.7	7107.6
207	U	6333	2.75	926.7	53.3	6331	2.56	164.2	19.7	762.4
208	C	6317	3.30	8635.7	65.9	6315	3.11	3033.7	40.0	5602.0
209	U	6303	2.75	1223.4	75.5	6302	2.56	282.4	19.0	941.0
210	C	6289	3.20	7862.3	100.6	6287	2.80	1716.6	26.1	6145.7
211	A	6274	3.45	13365.9	109.9	6272	2.56	1154.7	30.4	12211.2
212	A	6259	3.30	10914.8	123.4	6257	2.56	1402.3	25.1	9512.6
213	C	6244	3.15	6619.9	102.0	6242	2.71	1356.9	34.3	5263.0
214	A	6229	3.06	10984.9	127.9	6227	3.06	2211.5	24.1	8773.3
215	G	6217	3.19	6029.6	128.1	6215	2.84	1756.6	30.6	4273.0
216	A	6202	3.43	9768.1	112.2	6201	2.16	250.3	21.5	9517.8
217	C	6187	3.36	15924.3	141.3	6185	3.08	1674.5	30.3	14249.8
218	A	6173	3.57	24675.2	195.3	6171	3.26	3610.9	35.8	21064.3
219	A	6158	3.62	12013.8	121.3	6156	2.16	263.3	26.5	11750.5
220	C	6143	3.50	26378.8	80.0	6141	3.11	2862.6	26.4	23516.2
221	A	6129	3.43	13777.7	52.0	6127	2.84	1888.1	26.9	11889.6
222	A	6115	3.08	4517.7	78.6	6113	2.16	0.0	16.8	4517.7
223	C	6100	3.23	12440.5	88.3	6098	2.16	612.2	11.9	11828.4
224	A	6085	3.23	15299.0	99.3	6083	2.16	1052.0	14.4	14247.0
225	C	6069	3.08	7442.3	97.6	6067	2.16	235.6	19.6	7206.7
226	C	6053	3.28	8279.5	85.6	6051	3.04	2225.8	28.7	6053.7
227	U	6040	3.43	7603.7	58.3	6038	3.36	3244.3	22.5	4359.4
228	G	6028	3.08	198.0	114.0	6026	2.16	0.0	42.5	198.0
229	C	6016	3.29	13102.2	135.1	6014	2.90	1372.2	42.8	11730.0
230	U	6002	3.43	10370.4	103.8	6000	3.16	3330.6	40.7	7039.8
231	U	5989	3.08	4208.8	90.4	5987	2.37	1123.7	30.1	3085.2
232	C	5974	3.28	14127.6	123.1	5972	2.66	1717.3	34.1	12410.3
233	A	5959	3.38	15651.0	126.9	5956	2.76	2313.1	42.0	13337.9

234	U	5944	3.08	4150.8	98.3	5942	2.16	737.1	20.5	3413.7
235	C	5928	3.08	10290.3	89.0	5926	2.40	1216.9	18.7	9073.4
236	A	5912	3.08	9251.1	99.9	5910	2.80	1919.9	41.2	7331.2
237	G	5899	3.14	8750.4	90.1	5896	3.12	3071.0	35.5	5679.4
238	C	5886	3.23	8456.5	75.4	5884	2.87	2201.7	29.5	6254.9
239	U	5873	2.40	1445.0	76.5	5869	2.63	938.2	22.7	506.8
240	G	5862	2.57	1213.0	56.4	5860	2.49	856.0	15.3	357.0
241	U	5849	2.33	2332.4	29.3	5847	2.46	1069.0	17.0	1263.4
242	U	5834	2.33	982.5	72.0	5833	1.12	258.7	23.7	723.7
243	C	5820	3.09	10640.8	87.7	5818	2.68	1486.1	26.7	9154.7
244	C	5805	3.18	15816.5	121.5	5803	3.05	2310.5	34.6	13506.1
245	A	5791	3.33	16388.1	161.3	5788	3.46	3230.4	24.5	13157.8
246	G	5779	3.43	9054.8	153.0	5777	3.30	3655.8	38.1	5399.0
247	A	5766	2.70	3956.8	68.6	5764	3.06	1816.2	28.8	2140.6
248	G	5755	3.38	2092.4	77.4	5753	3.32	1131.1	20.1	961.3
249	A	5742	3.19	19099.8	165.7	5740	2.64	1139.7	26.8	17960.1
250	A	5728	2.42	3499.7	129.5	5726	1.12	82.8	13.0	3416.8
251	C	5714	2.89	7728.1	138.0	5711	1.12	113.6	8.7	7614.5
252	C	5699	3.10	10306.6	111.7	5697	1.38	284.0	16.4	10022.6
253	C	5684	3.29	17310.6	104.0	5682	2.16	755.2	21.8	16555.4
254	C	5669	2.99	4751.9	122.0	5667	1.72	557.1	22.5	4194.8
255	C	5655	2.75	4063.7	83.3	5652	2.42	987.2	27.7	3076.5
256	A	5640	2.94	6120.9	68.9	5638	2.72	1944.7	29.5	4176.2
257	U	5627	2.33	1308.3	39.9	5625	1.32	186.9	20.0	1121.5
258	C	5613	2.69	3483.2	46.8	5611	2.16	1233.6	14.0	2249.7
259	A	5599	2.71	1844.7	50.6	5598	3.12	58.9	32.6	1785.8
260	U	5587	3.14	5258.1	54.9	5585	3.06	2386.4	21.5	2871.7
261	G	5574	2.65	275.9	61.7	5572	2.17	40.0	16.5	236.0
262	C	5561	3.50	6374.7	87.1	5559	2.94	1419.7	28.9	4955.0
263	C	5547	3.36	13370.1	165.4	5545	3.17	2305.4	31.6	11064.7
264	U	5534	2.65	108.6	87.7	5532	2.25	133.0	28.0	-24.5
265	C	5520	3.04	5730.0	73.0	5518	3.12	2559.7	36.2	3170.3
266	U	5508	2.65	1773.3	46.8	5506	2.17	484.0	19.0	1289.2
267	C	5493	2.80	3405.4	59.3	5491	2.57	1190.8	18.1	2214.6
268	C	5479	3.12	8328.2	85.8	5477	2.87	2853.9	37.5	5474.3
269	U	5465	2.65	1696.7	72.0	5463	2.17	0.2	14.0	1696.5
270	C	5452	2.65	2281.1	81.4	5449	2.17	43.2	8.9	2237.9
271	A	5439	3.44	18694.8	63.7	5437	2.33	1091.1	19.6	17603.7
272	A	5425	3.44	23989.8	59.6	5423	2.41	1273.1	19.4	22716.6
273	C	5411	3.30	9548.9	66.3	5408	2.17	38.8	10.5	9510.1
274	C	5397	3.16	4349.8	45.9	5394	2.70	970.5	30.6	3379.3
275	U	5384	3.20	8608.4	42.2	5382	3.03	3728.0	36.5	4880.3
276	G	5372	2.65	2256.4	60.2	5370	2.18	690.3	40.8	1566.0
277	C	5358	2.65	2875.6	37.9	5356	2.53	1322.6	28.1	1553.0
278	U	5344	2.65	1829.4	70.6	5342	2.17	809.0	26.0	1020.5
279	U	5331	3.65	9182.4	147.3	5329	4.78	2471.2	75.4	6711.2
280	C	5317	3.50	49955.4	318.0	5315	4.32	3918.5	103.4	46036.9
281	A	5304	3.29	13582.4	136.2	5301	2.67	1447.3	26.9	12135.1

282	G	5293	3.40	2854.1	79.4	5291	2.79	858.6	15.5	1995.5
283	U	5282	2.72	2991.2	50.5	5280	2.88	1459.6	21.1	1531.6
284	A	5269	2.42	2000.4	51.4	5266	2.64	981.9	18.3	1018.5
285	C	5255	2.26	1552.0	68.8	5252	3.04	780.3	30.0	771.7
286	C	5241	3.40	17564.7	47.5	5239	3.48	3187.2	27.6	14377.5
287	A	5228	3.40	21285.9	54.5	5226	2.20	321.6	23.3	20964.3
288	C	5214	2.21	964.4	46.0	5210	2.20	197.0	14.6	767.4
289	C	5199	3.05	6567.3	49.1	5197	3.13	2166.2	17.8	4401.1
290	U	5186	2.55	1718.4	55.8	5184	3.18	873.3	17.5	845.1
291	C	5174	3.10	7977.9	120.2	5171	3.32	1865.0	20.6	6112.9
292	C	5160	3.24	12292.3	131.2	5157	3.42	2710.2	24.3	9582.1
293	A	5146	3.40	17277.1	146.7	5143	3.68	2671.8	33.2	14605.3
294	C	5131	3.45	22602.6	138.6	5128	3.65	3605.0	42.9	18997.5
295	A	5117	3.43	28724.1	123.9	5115	3.65	5247.1	51.4	23477.1
296	G	5104	3.60	6595.8	75.8	5102	3.68	2631.8	35.9	3964.0
297	A	5089	3.29	21883.3	216.0	5087	3.83	2450.3	29.2	19433.0
298	A	5075	3.37	24633.7	236.9	5072	3.70	3793.9	37.1	20839.8
299	U	5063	3.45	5811.4	93.5	5060	3.01	2735.4	34.5	3076.1
300	G	5052	3.03	2464.4	30.3	5052	2.90	900.7	34.8	1563.7
301	G	5041	2.71	2396.5	45.6	5044	4.75	398.1	55.4	1998.4
302	G	5030	3.64	12250.2	99.3	5028	4.10	4179.6	51.2	8070.6
303	C	5016	3.60	29113.3	129.4	5014	3.72	4135.5	37.3	24977.8
304	C	5005	2.89	2165.0	72.3	5001	2.95	780.0	14.6	1385.0
305	G	4993	3.50	690.7	45.6	4990	3.20	1.6	17.0	689.1
306	U	4981	2.21	726.5	27.9	4978	2.20	385.3	23.2	341.2
307	A	4966	2.82	5939.7	80.8	4963	2.95	2156.6	28.9	3783.2
308	C	4951	2.46	2360.5	87.8	4948	2.77	1172.7	19.9	1187.8
309	C	4938	3.09	2211.6	92.7	4934	2.79	1230.9	21.1	980.6
310	C	4926	3.21	14046.6	133.2	4924	3.53	2599.6	37.6	11447.0
311	A	4912	3.42	25955.7	165.1	4909	3.75	2530.9	32.8	23424.8
312	C	4898	3.42	26760.0	127.8	4896	3.39	3803.3	30.5	22956.6
313	A	4885	3.16	6274.1	92.2	4883	3.13	1494.1	17.1	4780.0
314	G	4874	2.41	784.8	85.3	4871	2.20	4.2	16.0	780.6
315	C	4862	2.81	4984.3	66.2	4859	2.76	2153.7	32.2	2830.6
316	A	4848	3.02	5967.1	55.5	4845	3.01	2122.2	40.5	3844.9
317	G	4837	3.07	6893.5	72.3	4834	3.05	2732.7	46.2	4160.9
318	U	4825	3.02	10216.8	83.7	4823	3.10	4016.2	41.4	6200.6
319	G	4814	2.23	1349.0	73.3	4812	3.15	406.8	20.3	942.2
320	C	4800	2.82	5120.7	82.0	4797	2.96	2495.5	24.2	2625.2
321	A	4787	3.02	6130.9	81.4	4784	3.20	2529.7	34.6	3601.1
322	U	4774	3.07	4536.9	61.8	4771	3.10	2318.7	50.3	2218.2
323	G	4764	2.90	6601.0	80.0	4762	2.92	2355.7	29.3	4245.3
324	A	4752	2.41	2073.6	41.0	4749	2.75	807.5	29.4	1266.2
325	U	4741	2.15	195.1	17.0	4739	2.20	364.7	12.7	-169.7
326	G	4731	2.15	43.8	86.0	4728	3.02	231.6	21.7	-187.8
327	A	4718	3.10	20036.8	174.5	4716	3.40	2251.8	28.3	17785.0
328	C	4704	3.15	21835.0	237.5	4702	3.40	2375.1	35.1	19459.8
329	C	4690	3.20	22019.7	265.2	4688	3.43	4002.2	45.7	18017.5

330	C	4677	3.05	20505.8	327.2	4675	3.38	4342.5	46.9	16163.3
331	A	4666	2.80	11227.5	201.8	4663	3.24	1326.2	30.5	9901.3
332	A	4654	3.20	20076.4	225.0	4650	3.28	1971.3	26.7	18105.1
333	A	4641	3.25	24477.2	126.8	4638	3.00	2242.2	36.2	22235.0
334	A	4628	3.05	16738.2	173.7	4625	2.31	1131.1	26.5	15607.1
335	C	4615	2.90	8527.2	165.3	4612	2.81	1065.3	45.8	7462.0
336	C	4602	3.05	18160.0	189.3	4600	3.05	2919.9	26.0	15240.1
337	A	4590	3.05	18096.8	189.3	4587	3.05	1949.5	26.8	16147.3
338	A	4577	3.05	18696.9	170.8	4574	2.86	1915.5	45.9	16781.4
339	G	4566	2.13	2364.0	103.2	4563	2.05	534.6	20.9	1829.4
340	C	4554	2.80	8534.5	140.3	4551	2.45	1526.5	22.3	7008.0
341	C	4541	2.62	5407.0	126.9	4538	2.36	1437.0	22.8	3970.0
342	A	4529	2.86	11498.9	125.9	4526	2.05	375.3	22.0	11123.6
343	A	4515	2.80	11367.7	192.3	4513	2.05	1308.5	29.0	10059.2
344	U	4504	2.54	6202.6	67.9	4501	2.05	789.3	26.6	5413.3
345	C	4492	2.05	2024.9	92.8	4489	2.05	471.2	22.6	1553.7
346	C	4479	3.00	16918.3	210.8	4476	2.75	2240.7	35.9	14677.6
347	A	4466	2.95	15513.1	133.7	4463	2.60	2143.2	33.3	13369.9
348	U	4453	2.05	1972.7	174.8	4449	3.15	501.8	44.6	1470.9
349	C	4439	3.05	23266.5	260.5	4436	3.05	4145.7	53.7	19120.8
350	U	4427	2.82	9418.4	118.6	4424	2.90	3947.6	47.6	5470.9
351	G	4416	2.55	2615.5	91.5	4414	2.85	1125.5	32.7	1490.0
352	G	4406	2.05	1056.9	59.5	4402	2.05	483.3	29.9	573.5
353	U	4394	2.45	5100.7	48.9	4391	2.80	2500.7	31.8	2600.0
354	U	4382	2.46	3896.7	52.6	4379	2.84	2069.7	31.3	1827.1
355	G	4372	2.87	4052.8	131.3	4369	3.15	2164.3	44.8	1888.5
356	G	4362	3.05	17188.8	119.7	4359	3.20	5483.4	33.3	11705.4
357	U	4351	2.90	7285.9	180.6	4348	3.30	3023.7	53.1	4262.1
358	C	4338	3.09	19393.6	242.1	4334	3.45	4525.3	47.3	14868.3
359	A	4326	3.20	23832.5	180.4	4322	3.07	4619.7	88.9	19212.8
360	U	4315	3.05	13856.8	116.1	4312	3.00	5661.1	71.0	8195.7
361	U	4303	2.23	2055.4	111.0	4300	3.07	1485.6	42.9	569.8
362	U	4292	2.05	2276.8	40.1	4289	2.45	1363.3	22.5	913.4
363	U	4280	2.05	1213.7	53.9	4277	2.45	477.6	20.7	736.1
364	A	4268	2.48	5807.5	112.0	4265	2.45	1839.3	44.3	3968.2
365	C	4257	3.05	2366.9	296.1	4253	3.03	639.1	46.5	1727.8
366	G	4247	3.62	5184.5	94.5	4249	2.45	295.1	52.4	4889.4
367	G	4237	2.51	3023.7	115.7	4243	4.50	2251.3	88.8	772.4
368	A	4226	2.05	1472.1	109.9	4227	2.45	0.0	84.9	1472.1
369	C	4214	3.00	13167.6	124.6	4211	2.65	1430.1	23.6	11737.5
370	A	4201	3.09	13086.1	157.4	4198	2.73	1150.5	26.4	11935.7
371	C	4187	3.05	20972.4	178.2	4184	2.80	1657.6	24.2	19314.8
372	C	4175	2.05	1258.8	133.7	4171	2.45	411.6	24.0	847.2
373	C	4162	3.00	13213.9	175.9	4158	2.80	2102.0	38.3	11111.9
374	A	4151	3.00	22803.5	198.0	4149	2.88	3212.0	47.1	19591.5
375	U	4140	2.99	8423.4	178.1	4138	3.10	1935.1	30.7	6488.3
376	C	4127	3.15	17305.1	157.7	4124	2.95	2517.2	37.5	14787.9
377	U	4115	2.90	9553.3	275.2	4113	3.15	3249.0	54.2	6304.3

378	A	4104	2.99	23764.7	344.7	4101	3.30	3610.7	39.7	20154.0
379	U	4093	3.35	12011.4	151.0	4090	3.35	4463.5	40.2	7548.0
380	G	4083	3.03	4892.2	204.2	4080	2.89	1440.0	82.1	3452.2
381	A	4072	2.65	7144.7	91.5	4069	2.04	1043.8	28.8	6100.9
382	U	4059	2.13	2406.1	96.6	4056	1.95	714.7	19.7	1691.3
383	U	4049	1.80	1442.1	51.3	4046	1.95	441.3	15.9	1000.8
384	C	4037	2.60	3775.4	181.4	4033	2.15	981.4	12.2	2794.0
385	C	4027	4.49	298.9	267.9	4027	3.99	1.6	63.9	297.3
386	G	4017	4.30	9075.2	221.9	4016	4.04	2855.5	45.5	6219.7
387	U	4006	1.80	570.7	126.3	4004	1.95	116.0	52.9	454.7
388	A	3995	2.60	6466.0	85.1	3992	2.97	2525.5	36.3	3940.5
389	U	3986	2.47	2319.2	125.5	3983	2.95	2041.5	33.7	277.7
390	A	3975	1.93	4170.3	219.5	3971	2.85	184.4	43.5	3985.9
391	C	3962	3.00	23136.4	241.5	3959	2.90	2231.3	32.1	20905.1
392	A	3951	3.03	23477.6	251.2	3949	2.95	2526.4	33.1	20951.2
393	C	3938	2.99	13006.1	338.2	3935	3.15	1778.1	23.1	11228.0
394	C	3926	2.75	11013.3	252.1	3922	3.00	2737.6	51.4	8275.8
395	U	3916	2.95	2804.4	180.6	3913	3.14	1722.6	57.5	1081.8
396	U	3906	2.46	6065.1	109.6	3902	3.10	3050.9	42.3	3014.2
397	A	3894	3.00	20117.9	253.0	3891	3.09	3523.1	44.7	16594.7
398	U	3883	3.18	6264.3	116.9	3879	3.29	2592.6	18.8	3671.6
399	C	3870	3.05	19192.0	176.3	3867	3.11	3976.7	28.4	15215.3
400	A	3859	3.00	17033.8	232.2	3855	2.86	2307.8	46.6	14726.0
401	A	3848	3.05	26841.2	218.5	3845	2.96	4070.0	57.6	22771.2
402	A	3837	3.13	15869.0	161.6	3833	3.29	1572.6	24.1	14296.4
403	U	3826	3.28	5044.3	133.9	3822	3.49	1645.2	35.8	3399.1
404	G	3816	3.28	2090.1	117.1	3812	2.92	609.0	73.2	1481.1
405	U	3805	2.43	682.1	153.2	3801	2.55	2084.8	45.2	-1402.7
406	C	3795	3.47	42353.7	303.1	3791	3.11	2067.5	52.7	40286.2
407	G	3784	3.81	3887.6	204.0	3781	3.16	695.7	58.2	3191.9
408	C	3773	2.43	2243.7	73.7	3770	1.54	348.9	15.1	1894.8
409	C	3760	2.63	2880.0	38.6	3756	1.60	490.6	14.9	2389.4
410	U	3750	2.43	4275.6	98.8	3746	2.54	906.4	36.9	3369.2
411	A	3740	3.45	9641.6	350.1	3735	2.49	2161.1	21.5	7480.5
412	U	3731	4.23	1634.5	154.4	3731	2.96	553.8	52.2	1080.7
413	G	3722	3.58	3217.7	37.0	3718	3.98	764.4	36.0	2453.3
414	U	3711	2.98	3838.8	100.8	3707	2.62	811.4	28.1	3027.4
415	A	3701	3.47	18351.9	118.4	3697	2.94	1589.9	27.2	16762.0
416	C	3689	3.53	8365.0	145.5	3685	2.92	1669.1	23.6	6695.9
417	U	3679	3.08	5119.3	103.0	3675	2.96	1808.8	27.8	3310.5
418	U	3669	3.23	13945.5	126.5	3665	2.96	2245.3	28.4	11700.2
419	U	3659	2.43	2374.6	80.9	3655	2.87	1079.7	19.1	1294.9
420	C	3648	2.43	0.7	85.2	3646	2.92	767.7	17.2	-767.0
421	C	3638	3.05	14517.8	144.4	3634	3.04	1943.5	19.4	12574.4
422	A	3627	3.13	2996.5	74.4	3622	2.94	611.4	22.5	2385.1
423	C	3613	2.50	1631.3	78.3	3609	2.48	430.3	21.0	1201.0

DMS_example_3_peaks.txt

seqnum	seq	RX.pos	RX.sigma	RX.area	RX.rms	BG.pos	BG.sigma	BG.area	BG.rms	(RX.area-BG.area)
7	A	9360	5.51	28794.4	392.9	9357	2.80	0.0	0.0	28794.4
8	A	9345	5.51	30913.3	389.0	9340	4.35	116.6	84.1	30796.7
9	C	9327	5.98	33737.4	435.6	9322	3.65	3075.8	103.6	30661.6
10	U	9310	4.80	7338.0	165.2	9310	5.39	4652.8	119.1	2685.2
11	U	9293	4.80	185.9	173.1	9291	2.85	0.0	106.0	185.9
12	C	9276	5.20	22721.2	152.0	9272	4.27	3845.1	89.4	18876.1
13	U	9259	4.87	14088.1	179.6	9257	5.29	4989.0	103.8	9099.1
14	A	9243	5.03	27061.7	200.2	9239	5.25	2636.8	87.4	24424.8
15	G	9228	4.80	1686.9	147.4	9221	5.00	854.2	78.9	832.7
16	U	9212	4.80	4147.6	85.1	9206	6.35	3041.6	201.8	1105.9
17	A	9196	5.10	7150.0	108.5	9191	5.55	23614.8	230.5	-16464.8
18	U	9178	5.15	654.5	99.5	9174	2.85	439.8	99.1	214.8
19	A	9162	4.09	5649.0	90.3	9157	3.25	1860.3	83.0	3788.7
20	U	9144	2.90	166.3	47.6	9145	2.85	574.2	83.7	-407.9
21	U	9129	2.95	1962.8	94.5	9126	3.37	2146.2	72.0	-183.4
22	C	9112	4.71	14226.9	138.8	9107	4.85	3686.1	89.5	10540.8
23	U	9096	4.90	8752.8	122.2	9091	4.00	4777.4	97.7	3975.5
24	G	9082	4.75	3873.3	53.3	9079	4.70	3392.1	92.5	481.2
25	U	9065	4.95	1299.0	60.0	9063	5.50	1182.9	68.6	116.1
26	A	9046	2.90	577.4	68.3	9046	1.80	1.6	18.5	575.8
27	U	9034	2.98	1156.4	83.7	9031	5.05	2713.4	76.8	-1557.0
28	A	9016	3.45	4461.4	171.8	9013	4.09	1479.0	69.8	2982.5
29	C	8999	2.90	1031.5	150.0	8996	5.15	1820.0	64.9	-788.5
30	C	8981	4.37	10656.4	111.0	8978	4.79	4146.8	86.9	6509.7
31	U	8965	4.90	5285.8	221.3	8963	4.21	3646.7	92.2	1639.2
32	A	8949	4.90	29036.3	320.0	8947	4.75	4608.8	113.8	24427.5
33	A	8933	4.66	17573.4	167.7	8927	5.65	2543.9	102.2	15029.6
34	U	8917	4.75	7895.7	184.6	8913	4.55	4508.6	109.4	3387.1
35	A	8900	4.95	30410.1	267.9	8897	4.65	5995.0	107.1	24415.1
36	U	8884	5.05	2613.4	202.3	8880	5.05	3016.0	90.9	-402.6
37	U	8868	4.60	7365.2	151.7	8864	4.70	4479.7	80.1	2885.5
38	A	8851	4.75	29184.6	210.4	8848	4.36	3523.2	70.2	25661.4
39	U	8835	4.69	55.9	162.6	8832	1.80	219.3	52.8	-163.3
40	A	8819	4.20	10402.9	108.7	8815	3.69	2802.5	76.5	7600.4
41	G	8803	3.45	2507.1	73.6	8804	3.31	1698.7	72.7	808.4
42	C	8787	3.45	1105.5	65.3	8785	2.59	331.2	53.5	774.2
43	C	8769	3.45	2777.7	60.0	8767	3.54	1032.6	39.9	1745.1
44	U	8755	3.45	1537.0	47.6	8750	2.83	411.8	41.8	1125.2
45	U	8739	3.45	1276.9	54.6	8735	4.01	440.7	56.0	836.3
46	U	8722	4.60	6033.2	139.8	8720	3.59	3204.6	83.1	2828.6
47	A	8706	4.50	30827.0	155.7	8703	3.59	2755.1	78.3	28072.0
48	U	8689	3.45	54.2	91.6	8684	4.20	714.7	84.0	-660.5
49	C	8673	4.83	15218.1	293.0	8669	4.49	4955.7	109.5	10262.4
50	A	8656	4.78	36126.6	303.0	8653	4.79	4587.0	103.7	31539.7

51	A	8640	4.80	27765.6	264.9	8636	4.69	2810.3	93.4	24955.2
52	C	8622	4.75	11445.3	242.6	8618	3.43	1842.9	99.7	9602.4
53	A	8605	4.83	26329.7	224.7	8601	3.14	1581.4	78.4	24748.3
54	A	8588	4.78	32647.4	230.2	8585	2.84	1633.3	77.6	31014.0
55	U	8573	4.70	8069.3	137.6	8571	4.74	4647.1	80.8	3422.1
56	G	8559	4.51	6395.6	78.1	8557	4.89	3912.3	71.4	2483.3
57	G	8544	4.35	156.6	69.7	8538	4.24	242.4	47.8	-85.8
58	A	8529	4.25	4803.2	69.7	8525	2.59	559.8	44.6	4243.4
59	A	8513	3.71	3994.0	55.4	8512	2.59	587.1	45.1	3406.9
60	U	8496	3.45	56.6	54.2	8495	3.09	512.8	42.8	-456.2
61	C	8481	3.80	8029.0	76.4	8478	3.59	2925.7	47.6	5103.3
62	C	8464	3.45	838.5	138.7	8461	2.66	803.8	71.8	34.7
63	C	8446	4.78	20839.2	196.4	8446	4.50	4707.5	130.4	16131.7
64	A	8430	4.80	37862.2	227.5	8428	2.72	3096.2	150.4	34766.0
65	A	8414	4.80	30759.3	247.8	8413	5.40	2338.6	183.1	28420.8
66	C	8397	4.83	42189.5	343.3	8394	4.65	8671.2	183.3	33518.4
67	A	8381	4.97	39851.7	411.4	8378	4.10	3760.8	118.8	36090.8
68	A	8364	4.85	30690.4	345.9	8362	4.13	6255.1	93.4	24435.3
69	U	8348	3.45	1751.3	78.9	8347	3.94	758.3	69.2	993.1
70	U	8334	4.75	8642.8	188.8	8332	3.75	4289.8	76.0	4353.1
71	A	8318	4.64	36599.3	266.2	8316	3.60	2843.6	68.0	33755.7
72	U	8302	3.69	3083.5	95.6	8301	3.85	1701.4	52.8	1382.1
73	C	8286	3.80	7743.0	74.7	8283	3.65	3381.7	49.6	4361.3
74	U	8271	3.45	370.9	77.4	8267	2.76	395.0	51.4	-24.1
75	C	8253	3.85	4933.9	87.2	8251	3.01	2297.9	67.1	2636.0
76	A	8238	4.40	20429.7	162.3	8237	3.89	2717.7	94.3	17712.0
77	A	8223	4.70	12901.9	160.8	8220	4.30	2505.7	80.8	10396.2
78	C	8205	4.80	5216.8	125.8	8204	4.51	2636.4	105.6	2580.3
79	A	8189	4.50	9240.3	76.0	8186	4.63	3259.4	93.1	5980.9
80	U	8173	4.34	1627.5	75.4	8174	3.55	1073.6	47.9	553.9
81	U	8158	3.45	425.6	44.7	8157	4.60	351.4	40.1	74.1
82	C	8142	3.45	0.0	93.3	8140	2.99	1328.8	66.9	-1328.8
83	A	8126	4.35	28392.0	159.0	8126	4.17	1677.1	90.0	26714.9
84	C	8108	4.21	16569.3	190.6	8107	3.92	1984.5	80.0	14584.8
85	C	8092	4.45	14904.3	269.5	8091	4.36	3651.5	115.0	11252.9
86	C	8075	4.46	40384.3	221.0	8073	4.27	5662.7	116.8	34721.6
87	A	8060	5.13	28717.2	830.6	8058	2.73	2374.1	78.3	26343.1
88	A	8041	5.52	26266.5	963.7	8041	2.98	1356.7	59.6	24909.8
89	U	8026	3.37	1193.1	127.7	8031	3.33	411.6	59.6	781.5
90	U	8016	4.62	1303.9	159.9	8011	2.50	936.2	56.0	367.7
91	C	7996	3.59	4146.0	106.2	7992	3.24	1543.7	45.1	2602.3
92	U	7979	3.37	882.8	55.0	7981	2.38	212.5	36.0	670.3
93	C	7963	4.75	4247.6	245.5	7960	2.63	1135.2	82.0	3112.5
94	A	7945	4.57	40542.6	320.1	7942	4.14	4470.3	100.6	36072.2
95	U	7929	3.89	6540.4	84.8	7929	5.07	4132.8	131.8	2407.6
96	G	7914	3.82	2210.5	71.1	7913	5.53	1137.3	75.4	1073.2
97	G	7899	3.37	662.0	69.3	7895	2.38	380.7	39.0	281.3
98	U	7882	4.27	3161.0	61.9	7883	4.40	1508.9	64.6	1652.1

99	A	7864	4.37	9300.5	113.7	7860	4.28	2333.0	64.1	6967.5
100	G	7849	4.32	2.0	141.3	7848	2.38	218.8	64.7	-216.8
101	C	7836	4.12	4318.1	59.3	7834	4.88	2239.7	56.9	2078.4
102	G	7821	3.37	468.5	72.9	7820	2.38	196.5	52.5	272.0
103	C	7806	3.68	6106.7	79.7	7806	2.86	804.7	64.9	5302.0
104	C	7790	3.99	9980.5	116.5	7789	4.13	1766.3	80.9	8214.1
105	U	7773	3.37	1467.1	95.3	7772	2.38	735.5	69.2	731.6
106	G	7760	3.37	342.4	67.7	7757	2.38	348.1	60.1	-5.7
107	U	7743	3.67	8569.7	78.0	7740	3.73	3191.7	78.3	5378.0
108	G	7729	3.57	2745.7	67.7	7727	3.57	1627.3	64.5	1118.4
109	C	7713	3.62	4311.5	50.9	7711	3.30	1768.8	62.6	2542.7
110	U	7697	3.37	3370.3	44.5	7696	3.08	1797.7	57.1	1572.6
111	U	7682	4.33	4939.9	111.0	7681	3.28	1637.9	64.2	3302.0
112	C	7664	4.43	14166.8	96.1	7662	3.63	4342.0	76.5	9824.8
113	G	7649	3.73	2675.0	88.7	7648	3.92	1461.6	51.0	1213.4
114	G	7634	3.13	1186.0	54.7	7631	1.54	207.7	52.1	978.4
115	U	7618	3.89	5446.1	68.1	7618	3.17	2453.0	95.2	2993.1
116	U	7604	4.22	8037.5	79.5	7601	3.68	4209.0	80.9	3828.6
117	A	7588	4.12	6257.4	91.0	7586	1.48	0.0	41.6	6257.4
118	C	7573	3.93	3405.4	61.9	7571	3.63	1695.7	49.1	1709.7
119	U	7558	3.65	4096.5	88.2	7557	3.68	2308.3	55.6	1788.3
120	U	7545	3.13	385.9	33.8	7540	1.78	624.2	52.7	-238.3
121	C	7529	3.13	33.1	25.4	7526	4.63	589.3	59.6	-556.3
122	U	7511	4.78	3347.4	179.5	7509	4.04	1834.4	92.2	1512.9
123	A	7494	4.41	33837.7	352.6	7493	4.38	4698.1	109.6	29139.6
124	A	7478	4.23	35568.6	233.7	7476	4.73	3601.7	80.1	31967.0
125	G	7465	3.32	2210.5	73.9	7463	3.65	1114.5	77.4	1096.0
126	G	7451	4.38	6896.1	57.1	7450	4.78	4543.2	86.5	2352.9
127	A	7436	4.41	4838.7	57.4	7434	4.63	2015.5	68.6	2823.2
128	A	7419	4.08	3332.6	82.4	7419	4.97	438.4	76.3	2894.2
129	G	7406	3.98	8156.1	76.1	7404	4.48	4837.5	87.7	3318.6
130	U	7392	4.23	1043.2	56.9	7389	2.04	608.3	43.0	435.0
131	C	7375	3.13	228.1	43.6	7375	1.48	220.9	21.3	7.2
132	C	7358	4.12	3890.9	62.2	7358	1.63	885.0	48.7	3005.9
133	A	7344	4.28	28470.2	117.2	7344	2.68	124.5	44.9	28345.7
134	C	7327	4.10	12630.2	151.9	7327	3.78	1874.5	64.1	10755.7
135	A	7312	3.87	6493.5	77.7	7307	1.82	178.8	67.1	6314.6
136	C	7295	4.09	6372.6	72.4	7294	2.96	1734.3	60.0	4638.3
137	A	7280	3.98	13891.8	104.3	7279	2.82	816.2	63.1	13075.7
138	A	7265	4.19	30728.4	191.1	7263	3.56	2993.9	81.0	27734.5
139	A	7249	4.24	39762.8	213.5	7246	3.41	3675.4	78.3	36087.5
140	U	7233	4.24	3208.2	190.5	7232	1.48	89.9	65.1	3118.3
141	C	7218	4.34	20346.5	122.0	7217	3.22	3494.8	141.1	16851.8
142	A	7202	4.19	42344.6	168.0	7200	4.01	7532.4	116.6	34812.2
143	A	7187	4.15	20411.5	154.0	7185	2.89	1069.4	57.8	19342.1
144	G	7171	4.34	1706.5	132.1	7170	1.48	100.4	28.3	1606.0
145	A	7155	3.34	4668.3	58.6	7155	1.48	0.0	7.8	4668.3
146	U	7140	3.62	3859.8	68.9	7139	1.70	850.1	37.9	3009.7

147	C	7125	3.74	5892.3	47.1	7125	1.48	675.7	53.1	5216.6
148	C	7109	3.34	3373.9	58.7	7108	3.16	1611.1	62.6	1762.8
149	G	7095	3.34	1465.6	63.6	7094	3.17	595.5	56.1	870.1
150	U	7080	3.58	4931.1	54.2	7079	2.96	2300.3	51.0	2630.8
151	U	7066	3.34	2066.3	52.2	7063	2.18	982.6	52.2	1083.7
152	A	7050	4.00	18008.4	155.9	7048	4.21	729.7	80.2	17278.7
153	G	7035	4.09	9747.1	112.5	7034	3.56	3864.8	98.1	5882.3
154	A	7021	4.09	30452.4	92.8	7018	3.12	1429.2	92.2	29023.2
155	C	7004	3.91	8993.3	87.0	7002	3.76	3505.6	68.7	5487.7
156	G	6991	3.44	2866.7	57.4	6988	3.34	2038.4	57.3	828.2
157	U	6976	3.11	2020.3	51.4	6977	2.71	1152.6	62.1	867.6
158	U	6963	3.36	2803.5	37.4	6960	3.07	2366.9	81.1	436.6
159	U	6946	4.54	19.1	75.6	6944	1.28	16.2	64.8	2.9
160	C	6930	3.89	9329.2	147.7	6929	4.07	3941.8	99.0	5387.4
161	A	6916	3.88	21853.8	182.4	6913	3.83	2697.4	94.4	19156.4
162	G	6901	4.11	634.8	88.5	6900	1.28	78.5	37.8	556.4
163	C	6886	2.88	498.1	43.3	6883	1.28	293.7	23.3	204.5
164	U	6873	3.45	1597.9	54.4	6873	1.28	403.2	42.3	1194.7
165	U	6859	2.88	959.3	49.7	6858	1.28	348.5	36.9	610.7
166	C	6842	2.88	302.4	35.8	6840	1.71	169.7	17.2	132.8
167	C	6826	2.88	3003.9	59.1	6823	1.28	371.1	14.5	2632.8
168	A	6811	3.38	6706.6	115.1	6808	1.28	0.0	10.3	6706.6
169	A	6796	3.73	16029.5	75.2	6792	1.28	66.1	9.6	15963.4
170	A	6781	3.88	23514.1	135.6	6779	1.28	72.9	16.8	23441.3
171	A	6765	3.88	32749.9	143.7	6763	1.52	1033.1	33.1	31716.7
172	C	6749	3.73	22056.4	98.9	6747	2.28	1423.1	57.6	20633.3
173	A	6734	3.68	9055.8	104.8	6731	2.55	1499.2	71.3	7556.6
174	G	6720	3.45	4838.3	90.4	6718	3.37	3224.3	71.9	1614.0
175	A	6704	3.58	7666.2	85.4	6703	3.43	3517.4	63.6	4148.9
176	A	6689	3.37	8469.0	88.4	6687	3.03	2135.7	58.7	6333.3
177	G	6676	2.88	391.8	92.7	6674	2.58	295.4	73.9	96.3
178	A	6660	3.58	9725.2	75.8	6659	3.23	2701.3	88.7	7024.0
179	A	6645	3.38	7215.6	66.9	6642	3.78	1428.6	51.0	5787.0
180	U	6630	3.38	2430.1	60.5	6629	2.58	1135.0	47.6	1295.1
181	G	6616	3.38	0.0	39.8	6614	3.33	646.1	61.2	-646.1
182	U	6601	3.86	4810.3	76.0	6601	3.58	2773.4	63.9	2036.9
183	G	6588	3.85	3428.9	79.2	6587	3.77	2922.4	89.7	506.4
184	A	6572	3.53	4537.0	52.1	6567	3.58	1592.2	96.1	2944.8
185	G	6559	3.55	5449.2	61.0	6558	3.16	3332.4	88.5	2116.8
186	A	6544	4.04	10880.2	113.7	6544	3.04	1455.7	85.8	9424.6
187	A	6528	3.93	26772.8	206.5	6525	3.14	1631.4	80.4	25141.5
188	G	6515	3.38	3439.9	47.1	6514	2.80	1635.6	57.3	1804.3
189	G	6502	3.38	622.2	41.0	6503	2.92	105.3	24.4	516.8
190	C	6488	3.38	939.9	35.2	6488	3.52	48.1	33.1	891.7
191	U	6474	3.73	2722.2	33.8	6473	2.95	1706.2	46.5	1016.0
192	U	6460	3.98	21.9	45.0	6459	2.58	177.0	36.6	-155.2
193	C	6445	3.38	1852.5	60.8	6444	3.90	1211.0	50.6	641.5
194	C	6430	4.04	17279.5	163.7	6428	3.67	4642.3	82.8	12637.2

195	A	6414	3.96	15503.6	191.7	6413	3.52	2258.5	84.2	13245.1
196	C	6399	3.38	3437.7	64.8	6396	3.52	1721.1	71.2	1716.7
197	U	6384	3.58	3664.4	54.2	6384	2.84	2097.5	54.1	1566.9
198	A	6370	3.62	7127.7	70.4	6371	2.75	456.1	75.0	6671.7
199	A	6355	3.78	11684.7	105.9	6352	3.47	3604.2	77.6	8080.6
200	G	6341	3.83	5429.8	85.6	6340	3.92	3164.0	112.2	2265.8
201	G	6329	3.64	12530.5	67.9	6327	3.80	5212.2	107.3	7318.4
202	C	6314	2.87	399.1	53.8	6313	2.15	143.6	49.5	255.5
203	U	6301	3.50	6736.1	58.1	6300	2.98	3581.9	58.0	3154.1
204	A	6288	3.83	10940.4	68.0	6285	2.15	699.3	56.0	10241.1
205	A	6273	3.83	25645.0	76.0	6271	2.67	1403.1	46.0	24241.8
206	C	6258	3.23	4978.9	53.4	6256	2.53	2072.1	41.7	2906.8
207	U	6244	3.63	740.2	48.4	6244	2.15	93.5	96.2	646.7
208	C	6229	3.50	6897.7	66.1	6227	3.31	8643.9	106.0	-1746.2
209	U	6215	2.87	365.6	52.3	6215	2.15	117.5	55.5	248.1
210	C	6201	3.22	4057.3	81.2	6199	2.41	2057.0	56.9	2000.2
211	A	6187	3.42	13778.7	118.4	6185	2.35	1095.7	65.5	12683.0
212	A	6172	3.23	8820.0	124.9	6169	2.85	2308.7	64.6	6511.2
213	C	6156	2.87	2693.9	99.2	6155	2.77	2760.2	71.1	-66.3
214	A	6142	2.87	8269.8	103.3	6140	2.15	1571.0	72.6	6698.8
215	G	6129	2.87	4578.0	84.8	6128	2.58	2343.4	57.9	2234.6
216	A	6116	3.06	7791.1	142.9	6114	2.15	0.0	51.6	7791.1
217	C	6100	3.55	11174.7	170.8	6100	2.15	1325.1	68.1	9849.5
218	A	6086	3.67	32885.6	165.8	6085	2.58	3369.2	63.1	29516.4
219	A	6072	3.43	11942.0	116.9	6070	2.15	0.0	74.7	11942.0
220	C	6057	3.62	32606.1	121.8	6055	2.77	3913.8	87.2	28692.3
221	A	6043	3.42	17955.0	114.9	6041	2.15	1715.2	50.2	16239.8
222	A	6029	2.87	5089.6	107.1	6027	2.15	0.0	55.9	5089.6
223	C	6015	3.34	17898.5	151.5	6013	2.65	1882.8	72.5	16015.6
224	A	6000	3.37	19766.7	143.5	5998	1.57	1096.2	58.2	18670.5
225	C	5984	2.65	5215.1	136.9	5982	1.82	870.9	75.3	4344.1
226	C	5969	3.52	23804.2	174.4	5967	3.55	6791.5	89.7	17012.8
227	U	5956	3.57	8663.0	127.6	5954	3.60	5433.7	83.3	3229.2
228	G	5944	2.43	0.0	61.0	5942	3.90	117.7	57.4	-117.7
229	C	5931	3.04	8468.2	71.4	5929	2.94	1816.3	52.4	6651.9
230	U	5918	2.90	4956.8	57.7	5916	3.10	3144.8	67.4	1812.0
231	U	5904	2.43	773.0	64.5	5904	1.52	106.5	45.7	666.5
232	C	5890	3.16	7801.1	107.3	5887	2.45	1595.9	58.4	6205.2
233	A	5875	3.32	13762.1	94.9	5872	2.02	979.1	41.9	12782.9
234	U	5860	2.43	1323.4	89.6	5857	1.52	125.5	38.6	1197.8
235	C	5845	3.15	10447.1	120.1	5843	2.35	1797.9	62.1	8649.2
236	A	5829	3.38	12602.1	137.0	5827	2.72	1694.5	74.6	10907.5
237	G	5816	3.24	9003.7	107.2	5814	2.75	2870.6	64.5	6133.1
238	C	5803	3.03	4760.2	74.1	5802	2.52	968.0	34.7	3792.3
239	U	5790	2.43	863.2	58.2	5786	1.52	45.1	23.2	818.1
240	G	5779	2.78	222.4	39.9	5777	2.56	453.1	33.8	-230.7
241	U	5766	2.43	1304.8	20.6	5765	2.75	36.5	25.2	1268.3
242	U	5752	2.43	0.0	26.2	5752	1.52	94.7	19.5	-94.7

243	C	5738	2.88	4803.1	75.3	5736	2.01	710.3	46.1	4092.8
244	C	5723	3.38	10325.7	74.9	5721	2.46	1834.1	75.7	8491.6
245	A	5709	3.38	18247.4	104.2	5706	3.27	4551.5	82.2	13695.9
246	G	5698	3.38	8494.6	117.0	5696	3.23	5740.9	78.9	2753.8
247	A	5684	3.46	4328.5	88.0	5683	2.43	1413.0	71.1	2915.5
248	G	5673	3.39	2740.5	83.4	5674	3.02	1138.3	52.2	1602.2
249	A	5661	3.43	28773.7	107.3	5659	3.17	1949.5	41.3	26824.2
250	A	5647	2.88	1365.6	105.5	5644	2.35	451.3	33.9	914.3
251	C	5633	2.88	4825.9	82.2	5630	3.42	814.1	42.5	4011.7
252	C	5618	3.02	6603.7	110.1	5619	2.22	519.4	54.6	6084.3
253	C	5604	3.36	18027.8	166.1	5602	2.22	1685.7	47.5	16342.1
254	C	5590	3.50	1965.1	89.2	5590	2.92	52.6	36.7	1912.5
255	C	5575	3.10	4873.7	52.8	5572	3.03	1610.3	41.8	3263.4
256	A	5561	3.23	6158.6	51.5	5559	2.83	1596.4	41.0	4562.1
257	U	5547	2.88	655.2	54.9	5544	2.22	27.6	33.8	627.6
258	C	5533	2.88	4221.5	49.2	5532	2.22	1702.1	28.4	2519.4
259	A	5520	2.88	2191.6	44.9	5519	3.08	27.7	52.7	2163.9
260	U	5508	3.08	3964.0	40.4	5507	3.08	2203.3	53.1	1760.8
261	G	5495	2.88	0.0	52.4	5495	3.14	1511.3	49.3	-1511.2
262	C	5483	3.35	5555.7	78.0	5481	3.60	5289.8	42.2	266.0
263	C	5469	3.30	8037.7	73.5	5467	3.27	6312.1	80.8	1725.6
264	U	5456	2.88	0.0	62.7	5454	2.93	687.6	120.6	-687.6
265	C	5442	3.23	8322.6	84.5	5441	3.80	23254.5	87.6	-14932.0
266	U	5430	2.88	2121.7	54.7	5428	3.80	12952.0	63.1	-10830.2
267	C	5416	3.19	4854.2	106.1	5414	3.75	24085.3	128.1	-19231.1
268	C	5401	3.45	12631.7	126.5	5400	3.75	41710.4	155.7	-29078.7
269	U	5389	2.88	985.9	67.1	5387	3.54	2804.7	93.4	-1818.8
270	C	5375	3.42	671.0	73.4	5374	2.73	1160.5	63.5	-489.5
271	A	5362	3.57	27024.2	108.0	5360	3.61	10926.7	66.4	16097.5
272	A	5348	3.47	30400.5	234.2	5346	3.60	15636.1	146.9	14764.4
273	C	5335	2.93	4747.0	115.2	5332	3.40	6225.5	108.5	-1478.5
274	C	5320	3.19	4230.5	71.0	5318	3.23	5809.7	73.4	-1579.2
275	U	5308	3.19	6265.7	63.4	5306	2.95	4099.3	69.4	2166.4
276	G	5295	2.90	1245.3	57.7	5294	2.02	286.3	47.4	959.0
277	C	5282	2.38	1610.0	25.8	5280	2.13	752.1	15.4	857.9
278	U	5268	2.38	853.4	75.2	5265	2.02	71.9	31.0	781.4
279	U	5254	3.48	9333.4	109.1	5254	2.90	829.5	44.9	8503.9
280	C	5242	3.48	55291.9	111.5	5240	2.66	2476.8	52.9	52815.1
281	A	5229	3.27	10109.4	82.3	5226	2.40	636.5	44.1	9473.0
282	G	5218	3.14	1782.0	40.9	5216	2.75	581.0	40.6	1201.0
283	U	5207	3.08	3447.8	41.2	5205	2.45	1163.4	29.9	2284.4
284	A	5194	2.64	1740.4	39.1	5191	2.02	162.5	19.1	1578.0
285	C	5180	2.38	513.5	55.4	5178	3.40	64.5	26.7	449.1
286	C	5167	3.52	19607.8	60.2	5165	2.77	1979.5	48.0	17628.3
287	A	5154	3.32	16611.5	145.8	5153	2.02	82.7	16.5	16528.8
288	C	5140	2.38	0.1	82.9	5143	2.02	310.6	23.2	-310.5
289	C	5126	2.94	3126.5	47.7	5124	2.72	734.1	28.8	2392.4
290	U	5113	2.38	511.8	56.6	5110	2.23	238.1	24.9	273.7

291	C	5100	2.62	2905.7	69.0	5097	2.39	744.5	28.6	2161.2
292	C	5087	3.32	9431.8	144.6	5085	2.87	1703.6	54.3	7728.3
293	A	5073	3.42	18661.3	116.4	5070	2.69	1569.2	56.6	17092.0
294	C	5058	3.48	17569.8	159.6	5057	2.78	2027.2	62.5	15542.6
295	A	5044	3.47	32802.3	354.3	5043	3.27	4821.2	64.7	27981.0
296	G	5032	2.45	4070.6	228.4	5030	2.44	1560.1	78.6	2510.5
297	A	5018	3.44	26798.1	162.7	5016	2.92	2241.0	59.5	24557.1
298	A	5003	3.44	30373.2	221.6	5001	2.88	2499.8	61.2	27873.3
299	U	4991	1.93	1870.6	154.1	4989	1.93	727.7	41.8	1142.9
300	G	4979	1.87	20.9	32.0	4979	2.33	459.7	22.1	-438.8
301	G	4969	2.35	1035.6	57.8	4968	3.16	859.5	31.7	176.2
302	G	4959	3.23	8484.4	84.6	4957	2.88	3245.8	60.9	5238.6
303	C	4946	3.42	15525.8	89.2	4943	2.60	1294.5	43.3	14231.2
304	C	4934	3.48	524.2	38.7	4929	1.30	298.4	25.2	225.8
305	G	4922	1.87	166.2	18.6	4920	1.12	89.7	14.6	76.5
306	U	4910	2.36	516.8	39.5	4909	1.12	114.4	18.1	402.4
307	A	4896	3.06	5432.0	43.8	4894	2.32	838.2	19.7	4593.8
308	C	4881	2.65	742.8	35.6	4882	1.12	24.9	10.0	717.8
309	C	4868	3.19	863.7	44.0	4867	1.12	0.0	5.9	863.7
310	C	4856	3.44	10440.4	90.0	4855	1.32	388.2	14.3	10052.2
311	A	4842	3.26	30152.3	206.4	4840	1.74	640.6	45.6	29511.7
312	C	4829	3.26	28316.9	177.7	4827	2.73	4887.4	86.6	23429.5
313	A	4816	3.43	5887.9	65.4	4814	1.12	143.5	44.8	5744.4
314	G	4805	1.87	692.0	64.8	4804	1.63	870.4	29.9	-178.4
315	C	4793	2.60	2417.8	57.1	4791	1.60	1501.9	42.2	915.9
316	A	4780	3.00	6195.6	92.8	4778	3.88	2932.7	152.0	3262.9
317	G	4769	3.16	7856.5	102.7	4766	3.14	11222.4	233.0	-3366.0
318	U	4757	3.25	9768.8	80.3	4755	3.10	15918.1	156.2	-6149.3
319	G	4746	2.73	1088.9	78.3	4744	3.29	5961.8	80.6	-4872.9
320	C	4732	2.94	4785.4	73.6	4729	3.05	5205.1	116.7	-419.7
321	A	4719	3.03	5684.1	54.5	4717	2.61	1962.3	65.0	3721.8
322	U	4707	3.13	3115.1	51.0	4706	3.68	1011.9	76.8	2103.2
323	G	4696	3.08	4747.2	66.8	4695	3.28	3328.0	43.2	1419.1
324	A	4684	2.73	1236.7	46.0	4682	2.30	531.3	31.2	705.4
325	U	4673	2.73	0.7	49.9	4673	1.53	0.0	15.9	0.7
326	G	4662	3.54	1351.7	92.3	4664	1.68	466.8	26.0	884.9
327	A	4651	3.20	28154.7	311.5	4649	2.57	1607.2	44.9	26547.5
328	C	4638	3.20	27748.2	324.7	4635	2.73	2335.8	51.9	25412.4
329	C	4624	3.35	17210.1	199.7	4622	2.95	2862.6	62.5	14347.5
330	C	4611	3.28	20039.4	117.9	4608	3.14	3274.3	76.9	16765.2
331	A	4599	2.85	14106.4	317.8	4598	1.65	642.3	53.2	13464.1
332	A	4588	3.20	27026.6	392.4	4585	2.73	1445.5	45.6	25581.1
333	A	4575	3.20	31751.0	318.9	4573	2.31	2040.3	53.6	29710.7
334	A	4563	2.84	17619.4	213.3	4560	1.73	632.2	29.6	16987.2
335	C	4549	2.73	2092.7	133.8	4548	2.34	92.9	38.7	1999.8
336	C	4537	3.08	13921.9	126.8	4535	2.47	2022.3	60.9	11899.6
337	A	4525	3.00	22178.6	253.7	4523	2.38	1748.7	63.5	20429.9
338	A	4513	2.99	20326.0	252.4	4510	2.87	2342.2	51.1	17983.8

339	G	4501	3.20	1076.0	136.9	4499	3.02	1282.4	44.1	-206.4
340	C	4489	2.53	5386.6	60.6	4487	2.97	3179.6	43.9	2207.0
341	C	4476	2.46	2397.6	96.2	4475	2.22	928.4	37.0	1469.1
342	A	4465	2.91	16773.2	179.7	4462	2.22	1063.2	35.8	15710.0
343	A	4452	2.91	12882.5	128.9	4449	2.65	1994.1	52.3	10888.4
344	U	4440	2.25	3470.8	95.0	4437	2.70	2089.5	47.8	1381.2
345	C	4428	2.39	0.1	105.4	4427	2.22	209.3	44.5	-209.2
346	C	4415	3.05	20409.8	201.6	4413	2.82	2972.4	50.4	17437.5
347	A	4403	2.91	17754.6	184.6	4400	2.22	1143.1	42.3	16611.6
348	U	4390	2.05	783.7	200.6	4386	2.65	2.0	30.2	781.7
349	C	4377	3.05	25104.0	304.0	4374	2.71	2150.0	32.7	22954.0
350	U	4365	3.20	9065.9	156.9	4362	2.82	4032.5	51.9	5033.4
351	G	4353	2.05	1384.1	89.9	4352	2.92	1036.9	28.5	347.2
352	G	4343	2.05	924.5	29.5	4340	2.22	522.3	33.0	402.3
353	U	4332	2.39	2556.1	55.2	4329	2.40	1345.0	41.5	1211.0
354	U	4319	2.48	2875.4	54.0	4317	3.08	1440.9	34.7	1434.5
355	G	4310	2.05	3043.4	145.9	4308	3.03	2484.4	77.2	559.0
356	G	4302	3.86	13782.5	583.9	4298	3.22	8895.2	72.7	4887.3
357	U	4289	3.55	9760.6	543.1	4287	3.32	2094.4	57.8	7666.2
358	C	4276	3.05	24546.6	258.5	4273	3.22	3829.8	57.6	20716.8
359	A	4265	3.05	24881.7	272.5	4262	3.22	5181.7	49.1	19699.9
360	U	4254	3.15	8249.9	150.5	4251	3.22	3852.7	40.7	4397.2
361	U	4243	2.65	0.0	54.0	4238	2.47	485.5	29.4	-485.5
362	U	4231	1.20	72.5	7.2	4226	2.61	782.1	21.3	-709.6
363	U	4219	1.20	0.0	54.9	4217	3.05	1162.4	39.5	-1162.3
364	A	4207	2.62	7547.3	134.3	4204	3.07	2334.1	53.4	5213.2
365	C	4196	2.77	1219.7	216.8	4194	3.20	988.7	97.8	231.0
366	G	4186	3.64	4292.0	98.4	4184	4.78	3815.6	50.8	476.4
367	G	4177	2.35	2137.7	82.1	4178	4.78	1635.3	63.4	502.5
368	A	4166	1.71	1111.1	85.1	4169	4.08	225.1	53.8	886.0
369	C	4154	2.81	9058.5	112.1	4153	2.86	1337.0	34.1	7721.5
370	A	4142	3.00	11515.5	121.1	4140	1.63	509.7	34.5	11005.8
371	C	4128	2.96	18580.9	106.8	4126	2.83	1379.2	36.9	17201.8
372	C	4115	1.20	0.2	96.4	4114	3.39	274.6	34.2	-274.4
373	C	4103	2.93	13129.6	188.9	4101	3.43	2588.9	65.6	10540.8
374	A	4092	3.20	35499.9	228.9	4090	3.05	3567.6	79.9	31932.4
375	U	4080	3.24	8657.8	150.4	4078	2.98	1914.0	59.4	6743.9
376	C	4068	2.91	15676.3	146.6	4066	3.01	2293.6	56.8	13382.6
377	U	4057	3.20	12341.4	192.5	4055	3.24	4421.5	70.8	7919.9
378	A	4045	3.00	35436.4	306.6	4043	3.39	4395.2	52.0	31041.2
379	U	4035	3.00	11632.4	268.3	4032	3.43	5190.4	55.2	6442.0
380	G	4025	3.20	4946.7	176.7	4022	3.21	2725.8	113.3	2220.9
381	A	4014	2.32	4271.4	58.6	4012	2.27	1494.0	40.3	2777.4
382	U	4001	1.20	431.0	49.8	3999	1.79	823.2	34.0	-392.2
383	U	3991	1.20	142.4	34.8	3989	1.63	537.2	14.8	-394.8
384	C	3978	1.65	1806.5	97.0	3976	1.63	213.9	17.0	1592.6
385	C	3968	4.50	170.7	229.7	3965	3.88	424.8	56.8	-254.2
386	G	3961	4.20	13021.7	171.3	3960	4.40	5328.5	90.0	7693.2
