# Supplementary material for: RNAthor – fast, accurate normalization, visualization and statistical analysis of RNA probing data resolved by capillary electrophoresis
Source: PLoS One. 2020 Oct 1;15(10):e0239287. doi: 10.1371/journal.pone.0239287 (PMC7529196; doi:10.1371/journal.pone.0239287)
Supplement: Attachment — Submitted filename: RNAthor-response-to-reviewers.pdf [file pone.0239287.s003.pdf]

We thank both Referees for reviewing the modified version of our manuscript "*RNAthor – fast, accurate normalization, visualization and statistical analysis of RNA probing data resolved by capillary electrophoresis*". All the comments allowed us to improve the quality of our work. We resubmit the revised version of the manuscript with changes highlighted in red. Below, we respond to all comments of the Referees.

Response to the comments of Referee #1:

I thank the authors for considering my suggestions. The revised manuscript presents a better case for the utility of RNAthor. The authors have explained why it is not necessary to have a standalone version of RNAthor at this time, added relevant references, as well as addressed my other minor comments. A good number of experimental biologists are indeed unskilled at even basic data analysis tasks. Such biologists could benefit from a web server where they can upload outputs of ShapeFinder or QuShape and save normalized data, relevant figures and results from statistical tests. Hence, as a software tool RNAthor seems to have utility.

The primary contribution of the manuscript is a web server that combines existing functionalities from other sources. The method for automatic identification of unreliable data is an implementation of the rules that they have utilized previously (e.g., in their articles in Nature Communications, RNA Biology). The statistical tests that they perform have been used by other researchers active in the field. So overall, there appears to be no novelty with regards to the methods.

In my previous review, my major concern was that the method to identify "unreliable probing data" has not been validated. In revision, the authors have shown that the results from RNAthor are comparable to those from their manual analysis. This serves as evidence that there are no bugs in the software and that the rules followed by the human analyst have been faithfully implemented. The differences that they observed can be explained by subjectivity of manual analysis. However, objective validation of the method is still lacking.

The authors claim that comparison of RNAthor with manual analysis is the only possible validation. I believe that at the very least, the authors could test a range of cutoffs to identify and exclude unreliable data. The results from different cutoffs could be objectively compared by examining the accuracy in reproducing well-studied RNA structures, or other biological results that are widely believed in the field to be true. In my assessment, stating that an implemented algorithm is based on experience is not enough to claim novelty of the method. To make such a claim, the authors must demonstrate that out of a set of other plausible and reasonable methods, the method implemented in the web server is the one that performs the best. The authors can take a look at the following paper from the Laedarach lab as an example. In this article, Woods and Laedarach automated some of the rules used by humans for manual analysis. They tested a range of algorithms to identify the one that performs the best.

Woods, C. T., & Laederach, A. (2017). Classification of RNA structure change by ‘gazing’at experimental data. Bioinformatics, 33(11), 1647-1655.

Response: We agree that presenting the results of extensive and sophisticated computational tests of the new program significantly increases its credibility. However, it is difficult to perform many tests in the absence of raw data that other laboratories do not provide. The idea of testing a range of cutoffs is very interesting. It is quite easy to implement for a single-criterion problem, but our procedure is a multi-criteria decision problem. RNAthor has several input parameters (which the user can turn on/off) and internal parameters (bgArea, areaDifference, effectiveMaximum) - they depend on one another and all of them impact the results. We do not see the possibility to test these dependencies in a reasonable time.

In summary,

1. I find the article acceptable if it is classified as reporting a software tool. I’d recommend that the authors tone down their claim of novelty with regards to “an algorithm for the automatic identification of unreliable probing data” as this claim requires objective validations that are not there. I understand that the authors may have added this claim in response to my earlier comment about lack of novelty. My comment was based on assessing the manuscript as a methods research article, which may not be the intention of the authors. So it is best to remove this claim. Also, the authors should make the raw data used for figures 3-4 available as supplementary data, or provide links if they are available online.

Response: From the very beginning, we intended to present a new software tool, not a new method. We agree that the paper should be classified as reporting a software tool. As recommended, we removed the claim of novelty regarding the unreliable data identification. We also prepared the file with supplementary materials that includes raw data used for Figures 3-4. These data are also available as example data in the RNAthor web application.

2. To be accepted as a methods research article, major revisions are still needed to demonstrate that the method is indeed optimal and applicable for general use. Lack of gold standards to objectively evaluate methods for analysis of RNA structure-probing data is a challenge faced by all methods researchers active in the field. However, the community has also found acceptable ways for such evaluation. If the authors did indeed mean to publish RNAthor as a methods research article, I hope that the authors will borrow ideas from other manuscripts and consider validating their method.

Response: We agree that our paper is not a methods research article. I suppose the misunderstanding was caused by the fact that sometimes we use the terms algorithm, method, procedure, and software tool interchangeably. To clear this issue, we have introduced several modifications in the manuscript.

Response to the comments of Referee #2:

The authors added several useful features to their web server, such as options to perform and visualize data-directed secondary structure prediction and to analyze DMS data. Hopefully, this would make the proposed tool more appealing to potential users, although overall, the main novelty in this work remains a fairly simple routine for automated detection of unreliable data points. A comparison between manual and automated data processing helps demonstrate that the automated detection is reliable/judicious, although I would expect to see more examples if I were a potential user, or at least manual analysis by additional experts (not the makers of the tool). Regarding the optional statistical tests, I think there are more statistically-sound and powerful differential reactivity analysis methods out there, and I don't feel what's offered by this tool is very powerful.

Finally, please note that the graphics is of poor quality and should be improved.

Response: Referring to the suggestion to include more example data, we would like to note that although we currently have one example for SHAPE and one example for DMS data on the RNAthor webserver, these are complex examples. Each of them consists of three raw data files obtained for a relatively large RNA. The user can download the example files and rework them - creating new examples (e.g. load only two data files instead of three, cut out the data for a selected particle, shorten the particle sequence). It seems to us that there is a large field here to test our program using the files we provided. We would be happy to give more examples if there are scientists from other labs who provide us with their data. Unfortunately, we have not managed to find raw CE data publicly available, which we could use. We still have sets of data from our laboratory, which we can add as running examples to the RNAthor webserver as soon as we publish the structures that have been determined based on these data.

As for the statistical tests, we agree that various statistical methods could be implemented apart from the ones we provided. So far, we included basic tests, which - to our knowledge - are used commonly in many labs (including Week's lab and our lab). But we are always open to suggestions of our users and we often add new options to our web servers if the users request them and explain why the new options are necessary. In the future, we will gladly enrich RNAthor with new features, including more powerful statistical tests.

Poor quality of the figures in the manuscript is probably due to very high compression on the PLOS server side. We can also see it in the pdf version generated by the submission server. All drawings in the original version meet PLOS requirements (tiff format, resolution 400dpi) and are of sufficient quality.
